# Transcriptome analysis reveals differentially expressed MYB transcription factors associated with silicon response in wheat

**DOI:** 10.1038/s41598-021-83912-8

**Published:** 2021-02-22

**Authors:** Lidong Hao, Shubing Shi, Haibin Guo, Jinshan Zhang, Peng Li, Yanfei Feng

**Affiliations:** 1 College of Agriculture, Xinjiang Agricultural University, 311 Nongda East Road, Urumqi, 830052 China; 2 College of Agriculture and Hydraulic Engineering, Sui Hua University, No.18, Huanghe Road, Suihua, 152061 China

**Keywords:** Agricultural genetics, Gene expression

## Abstract

Silicon plays a vital role in plant growth. However, molecular mechanisms in response to silicon have not previously been studied in wheat. In this study, we used RNA-seq technology to identify differentially expressed genes (DEGs) in wheat seedlings treated with silicon. Results showed that many wheat genes responded to silicon treatment, including 3057 DEGs, of which 6.25% (191/3057) were predicted transcription factors (TFs). Approximately 14.67% (28 out of 191) of the differentially expressed TFs belonged to the MYB TF family. Gene ontology (GO) enrichment showed that the highly enriched DEGs were responsible for secondary biosynthetic processes. According to KEGG pathway analysis, the DEGs were related to chaperones and folding catalysts, phenylpropanoid biosynthesis, and protein processing in the endoplasmic reticulum. Moreover, 411 R2R3-MYB TFs were identified in the wheat genome, all of which were classified into 15 groups and accordingly named S1–S15. Among them, 28 were down-regulated under silicon treatment. This study revealed the essential role of MYB TFs in the silicon response mechanism of plants, and provides important genetic resources for breeding silicon-tolerant wheat.

## Introduction

Silicon is the second most abundant element in the Earth’s crust^1,2^. In soil, the Si concentrations range from 14 to 20 mg Si/L, and therefore also found in plant tissues^3^. Silicon helps plant growth through its pivotal physic-mechanical role. It is considered a non-essential element for plant growth and development; however, it has been reported to increase plant grain yield in response to environmental stresses^4,5^. For example, Si-treated rice exhibited increased resistance to diseases such as leaf blast, stem rot, and sheath blight^6^. In a growth medium, silicon alleviated the stress effects of drought, salinity, and cadmium toxicity^2^. Several beneficial effects of Si have been reported, such as improving photosynthetic activity, reducing mineral toxicity, changing nutrient imbalance, and enhancing abiotic stress tolerance^3^. Studies have also shown that silicon protects crops such as wheat from pathogenic fungi, such as powdery mildew (*Blumeria graminis*). Si treatment reduces disease severity by increasing the activities of peroxidase (POD), polyphenol oxidase (PPO), and phenylalanine ammonia-lyase (PAL), especially when plants receive Si prior to aphid infestation^5^. In a wheat –*B. graminis* f. sp. tritici (Bgt) system, epidermal cells of plants reacted with Bgt attack with specific defense mechanisms^7^.

The Si concentration varies among plants. Monocotyledons tend to accumulate more Si in shoots than dicotyledons^3^. The Si shoot concentration of wetland grasses is between 5 and 7%, for dryland grasses is from 0.5 to 1.5%, while that of most dicotyledons is less than 0.5%^3^. Plants absorb Si from the soil solution in the form of silicic acid, and passively or actively transport it from roots to shoots^8^. The active mode of Si is transported in plants through specific transporters, which is characteristic of some monocotyledons, such as maize, rice, and wheat^9–12^. When plants encountered environmental stresses, the expression of some transcription factors, such as AP2/ERF and NAC, showed spatiotemporal change under Si induction^3^.

Wheat (*Triticum aestivum* L.) is one of the most important crops worldwide^13^ and previous studies have indicated that it has a high tendency to absorb and accumulate Si^2,13^. Many reports have demonstrated that Si decreases wheat leaf and root concentrations of Na^+^, K^+^, and Ca^2+^, leading to enhanced resistance to abiotic stresses^14–17^. In the current study, we performed RNA-seq analysis on total RNA extracted from wheat seedlings from control and silicon treatments to investigate the effects of Si on plant growth. Through this analysis, we identified 1164 and 1892 genes that were significantly up- and down-regulated, respectively, in response to silicon treatment. We carried out GO enrichment and KEGG pathway analyses, and identified TF families. Additionally, we showed that the RNA-seq profile was highly enriched, with MYB TFs playing a pivotal role in silicon treatment, and these MYB TFs were identified at a genome-wide level. This study also identified candidate genes for breeding silicon-tolerant wheat and provides new insights into the genetic basis of silicon tolerance in wheat. Our study may also be helpful in developing new and effective strategies for engineering wheat for silicon tolerance.

## Results

### Quality of RNA-seq data

RNA-seq of the six cDNA libraries resulted in 677.39 million raw reads, of which approximately 675.90 million clean reads were de novo assembled into contigs using an Illumina system. We performed transcriptomic analysis of the two samples (S0 and S1), with three biological replicates for each test, to profile the wheat response to silicon. Evaluation of the data quality showed that the length and the GC content of the reads were in accordance with the criteria (ReadsFilter > 90%, GC > 48%) (Table S1). The raw data were submitted to the NCBI database under the SRA accession number: PRJNA605071.

### Identification of differentially expressed genes (DEGs) and functional enrichment

**Figure 1:**
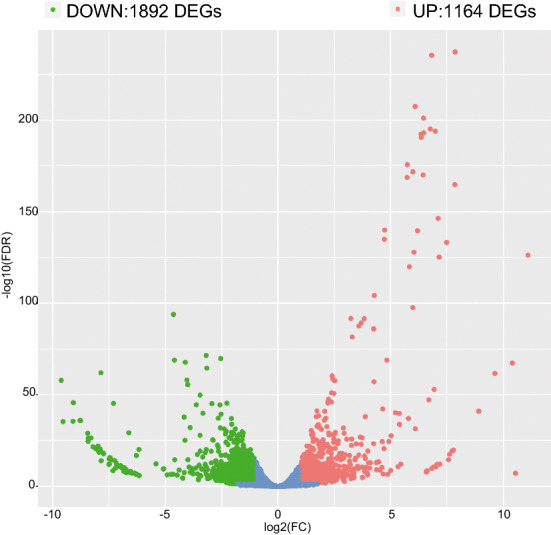
Heatmap illustration of differentially expressed genes (DEGs). Red and green circles indicate up-regulated and down-regulated DEGs, respectively.

As results, a total of 3056 genes were differentially expressed in wild wheat seedlings under silicon treatment compared with those in the control (Fig. 1 and Table S2), 1164 up-regulated and 1892 down-regulated genes were altered after silicon fertilizer treatment.

**Figure 2:**
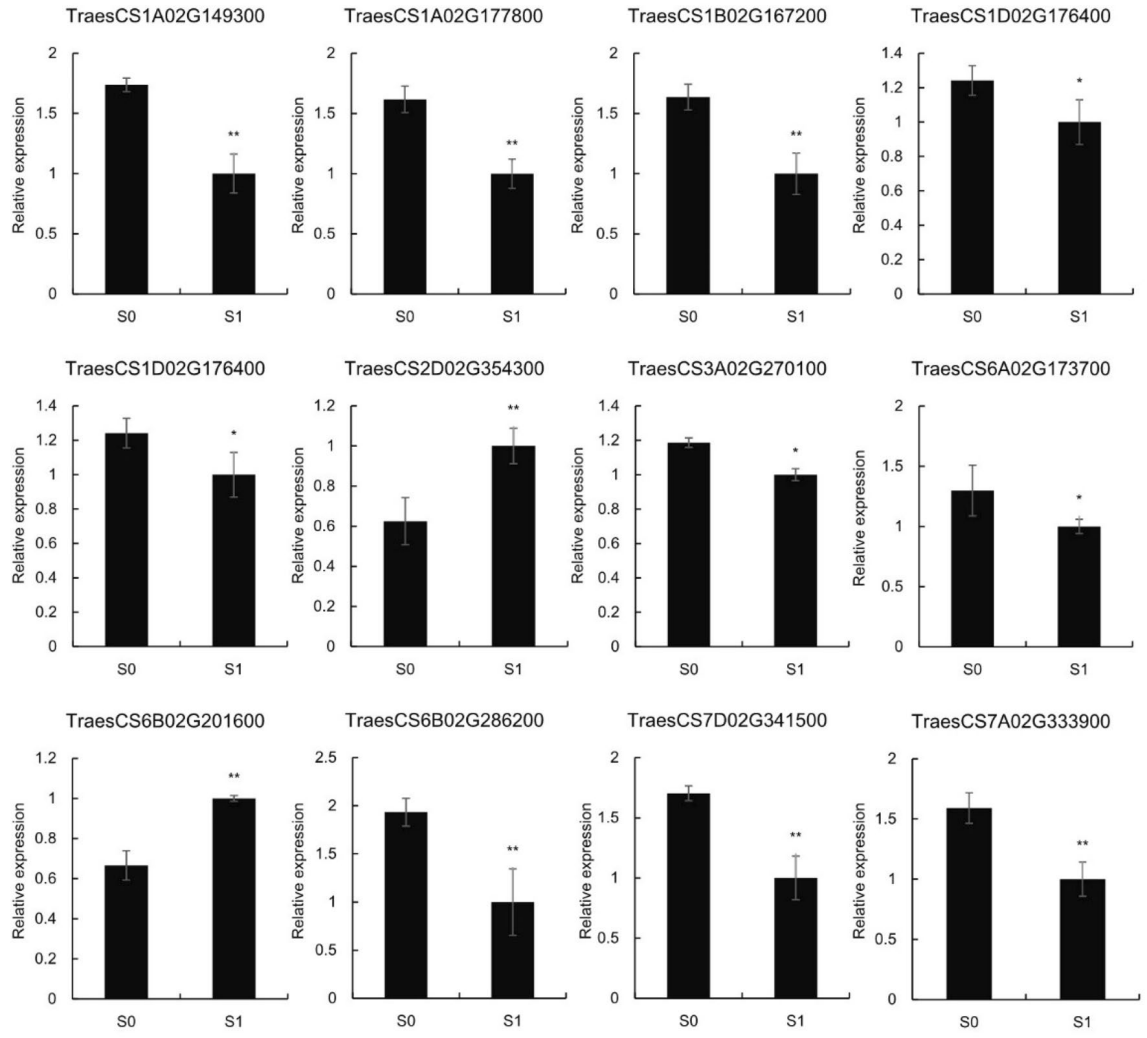
Expression profile of 12 randomly selected differentially expressed genes (DEGs) determined using qRT-PCR.

To further verify the expression level of the RNA-seq data, qRT-PCR was conducted, and the expression levels of 12 randomly selected genes were analyzed. The results showed that the expression trends were consistent with those of the RNA-seq data (Fig. 2 and Table S3).

**Figure 3:**
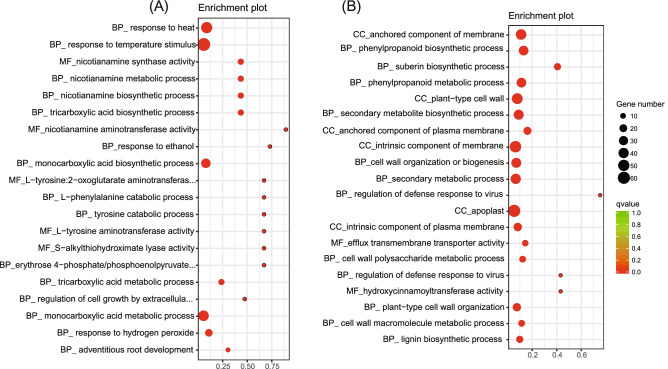
Analysis of GO enrichment for DEGs. (**A**) and (**B**) indicate GO enrichment for up- and down-regulated DEGs, respectively.

GO assignments were used to predict the functions of wheat unigenes and were classified based on various biological processes. Genes were classified into categories with three independent ontologies including biological process, molecular function, and cellular components. We performed GO enrichment analyses of DEGs using AriGO (qvalue < 0.05). After silicon treatment, genes with the molecular function of nicotianamine synthase activity (GO:0030410), and those involved in the biological process of nicotianamine metabolic process (GO:0030417) and nicotianamine biosynthetic process (GO:0030418) were significantly enriched in up-regulated genes (Fig. 3A). Whereas GO terms in cellular components of the anchored component of membrane (GO:0031225), biological process of phenylpropanoid biosynthetic process (GO:0009699) and suberin biosynthetic process (GO:0010345) were enriched in down-regulated genes (Fig. 3B).

All DEGs were BLAST searched the KEGG Ontology (KO) database. A total of 868 DEGs were classified into 48 biological pathways and found to be significantly enriched. As shown in Supplementary Figure S1, the most significantly enriched genes were involved in chaperones and folding catalysts (PATH:03110), phenylpropanoid biosynthesis (PATH:00940), and protein processing in the endoplasmic reticulum (PATH:04141).

### TF families are active during silicon treatment

**Figure 4:**
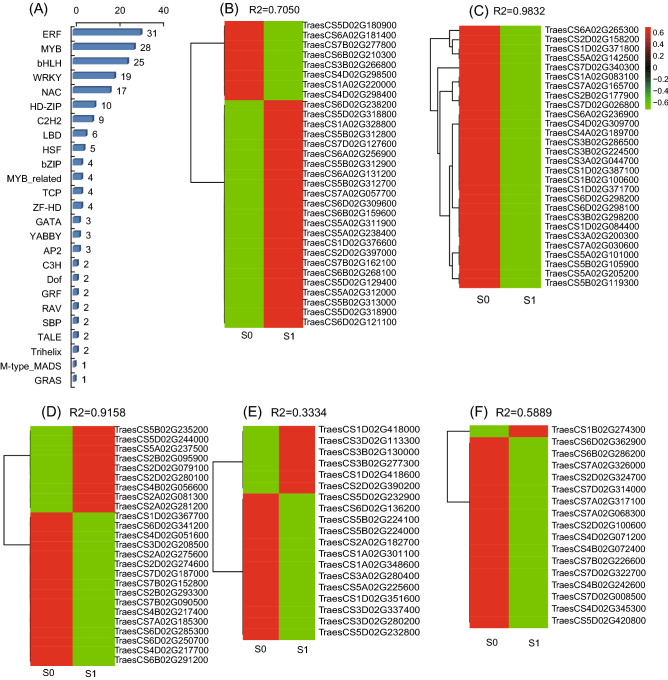
Functional classification and expression profiles of TFs. (**A**) Distribution of differentially expressed TFs in gene families. (**B**), (**C**), (**D**), (**E**), and (**F**) indicate expression profiles of ERF, MYB, bHLH, WRKY, and NAC TFs, respectively.

TFs are essential for the regulation of gene expression by binding to specific *cis*-elements in the genes that they regulate. A total of 191 TFs representing 25 different families were differentially expressed under silicon treatment compared to those with controls in wild wheat seedlings. Most of the identified DEGs encoded members of the ERF, MYB, bHLH, WRKY, and NAC families, and 20 TFs families included more than one differentially expressed TF (Table S4 and Fig. 4A). The ERF family, with 31 DEGs, was the largest TF family in response to silicon treatment. As shown in Fig. 4B–F, most of the differentially expressed TFs were down-regulated after silicon treatment. All MYB TF family members encoding R2R3-MYB TFs were down-regulated after silicon treatment; this had not been reported in previous genome-wide studies (Fig. 4C). Therefore, R2R3-MYB TFs were chosen and considered as candidate silicon-responsive genes in further experiments.

### Identification of wheat R2R3-MYB TFs

To identify wheat MYB TFs, a Hidden Markov Model (HMM) search and self-BLASTP were conducted, and HMM profile of the MYB domain were used as queries against the latest genome data of wheat from the Ensembl Plants database. A total of 584 sequences were identified as potentially encoding MYB domain(s)-containing proteins in the wheat genome. Subsequently, all putative genes were examined to determine the number of MYB domains. We found that the protein sequences of 158 MYB TFs contained one MYB repeat, 411 contained two MYB repeats, and 12 contained three MYB repeats, while the remaining 3 members contained four MYB repeats (Table 1). Transcriptome analysis showed that the MYB TFs that encode R2R3-MYB members were down-regulated after silicon treatment (Fig. 4C); thus, we selected the R2R3-MYB TFs for further analysis. To evaluate the existence of R2R3-MYB genes, the CDS were extracted from wheat and used to search against the wheat expressed sequence tag (EST) database using the BLASTN tool. Results showed that most *TaMYB* genes had one or more representative ESTs, and 40 genes showed no EST hits. *TaMYB* genes were distributed unevenly among the 21 chromosomes of the wheat genome (Table S5).

**Table 1:** Classes of MYB TFs in *A. thaliana*, *O. sativa*, and *T. aestivum*.

	Eudicot	Monocot	Monocot
MYB protein classes	*A. thaliana*	*O*. *sativa*	*T. aestivum*
R2R3-MYB	126	109	411
1R-MYB, MYB-related	64	70	158
3R-MYB	5	5	12
4R-MYB	1	1	3
References	^18^	^18^	

### Phylogenetic relationship and homoeologous analysis of R2R3-MYB TFs in wheat

To understand the evolutionary relationship of R2R3-MYB TFs in wheat, a Neighbor-Joining (NJ) tree was generated using the multiple sequence alignment of wheat MYB proteins. According to the clade support values, all wheat R2R3-MYB TFs were classified into 15 groups and named S1–S15 (Supplementary Figure S2). Among these 15 groups, group S9 was the largest group with 70 members, while group S3 had the smallest group with 5 members.

Among the 411 TaMYB genes, there were 140, 131, and 139 members distributed on wheat sub-genomes A, B, and D, respectively (Table S5), except for TraesCSU02G069000, were not classified. Based on the phylogenetic relationship, we also analyzed homoeologous groups in detail. As a result, approximately 78.10% (321/411) TaMYB TFs are present in homoeologous groups of three, 16.55% (68/411) TaMYB members containing two copies, and 5.35% (22/411) containing only one copy (Table 2).

**Table 2:** Groups of homoeologous R2R3-MYB TFs in wheat.

Homoelogous group	All wheat genes^1^ (%)		Wheat R2R3-MYB genes		
			Number of groups	Number of genes	% of genes
1:1:1	35.80		107	321	78.10
n:1:1/1:n:1/1:1:n^2^	5.70		0	0	0
1:1:0/1:0:1/0:1:1	13.20	A:B:0	5	10	16.55
		A:0:D	15	30	
		0:B:D	14	28	
Other ratios^3^	8.00		0	0	0
Orphans/singletons	37.10		22	22	5.35
	99.80		–	411	100

^1^According to IWGSC.

^2^For n > 1.

^3^E.g. n : 1 : n or 0 : 1 : n, n > 1.

### Phylogenetic and expression profiles of wheat R2R3-MYBs under silicon treatment

As shown in Supplementary Figure S2, the 28 differentially expressed wheat R2R3-MYBs were clustered into five groups according to phylogenetic analysis. Among them, group S7 was the largest with 14 members, while group S11 was the smallest with only one member.

**Figure 5:**
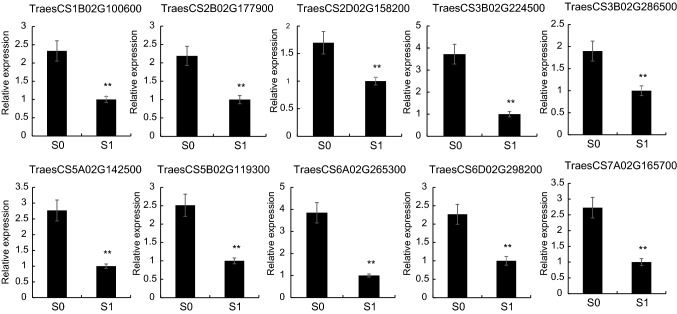
Expression profiles of 10 randomly selected differentially expressed MYB TFs by qRT-PCR.

A heat map was built to visualize the expression changes of all 28 R2R3-MYBs under silicon fertilizer treatment. As shown in Fig. 4C, the 28 differently expressed R2R3-MYB genes showed down-regulated expression levels. To further validate the expression changes of wheat R2R3-MYBs under silicon treatment, we performed qRT-PCR to analyze the transcripts of 10 randomly selected R2R3-MYBs. Results showed that the qRT-PCR results were consistent with the RNA-seq results (Fig. 5 and Table S3). These results further validated the reliability of the RNA-seq data.

## Discussion

Although Si is a major component of most soils^2^, its role in plant nutrition remains controversial because of differences in the abilities of plants to absorb it. In rice, the silicon concentration is about 10–15%, more than 90% of this being in the form of phytoliths^19^ which usually take the shape of the plant cells where it is deposited^20–22^. Until now, few studies have been reported on the role of Si in wheat, an important crop worldwide. In the current study, we performed RNA-seq analysis to investigate the effects of silicon treatment on the growth of wheat.

At the molecular level, the expression levels of 3057 DEGs in the wheat genome were altered after undergoing silicon fertilizer treatment (Fig. 1). These genes are mainly involved in secondary metabolism processes. GO enrichment analysis indicated that in response to silicon, most of the up-regulated genes were involved in nicotianamine metabolic and biosynthetic processes. The GO terms related to membrane, phenylpropanoid, and suberin biosynthetic processes were down-regulated. These results are similar to what has been shown in rice and tomato, where the expression levels of the majority of genes changed under silicon treatment^1,23^. In addition, our results indicated that several molecular mechanisms that were activated by silicon might be used to fight plant pathogens and for other production practices.

Of the 3057 DEGs found, 191 genes encoded 25 different types of TFs that regulate plant development and growth in response to silicon treatment. These results suggest that silicon treatment affects many biological processes and pathways by altering the expression of genes that encode TFs.

Among the high-frequency TF families, R2R3-MYBs were identified as the most abundant in our transcriptome data, constituting 14.66% of the total differentially expressed TFs. However, genome-wide identification of MYB TFs in wheat has not yet been undertaken. In this study, 411 R2R3-MYB TFs were identified in wheat, accounting for approximatly 0.54% of all annotated wheat genes, which is more than that of rice (0.3934%)^18^ and *Brachypodium distachyon* (0.39%)^24^, but less than that of *Arabidopsis* (0.60%)^18^.

The MYB group of TFs acts as regulators to regulators of plant growth and development^25,26^. For example, wheat R2R3-MYB protein TaPL1 and TaMYB1 function as positive regulators of anthocyanin biosynthesis and morphology^27,28^; in rice, R2R3-MYB gene *OsMYB103L* influences leaf rolling and mechanical strength^29^; and in *Arabidopsis*, *MYB21*, *MYB24*, *MYB33*, *MYB57*, *MYB65*, and *MYB103* are regulators of several pathways that affect anther development^30–33^. In addition, R2R3-*MYB* genes are involved in responses to biotic and abiotic stresses. For example, overexpression of wheat R2R3-MYB genes *TaMYB30*-B, *TaMYB33*, *TaMYB56*-B, and *TaSIM* confers tolerance to salt and drought stresses in transgenic *Arabidopsis*^34–37^. Silencing *TaMYB4* increased susceptibility to the biotrophic fungal pathogen *Puccinia striiformis* f. sp. *tritici* in wheat^38^.

Pathogen-induced MYB protein 1 (TaPIMP1) positively contributes host resistance to infection of the fungal pathogen *Bipolaris sorokiniana*^39^. However, research is limited to show the regulation of MYBs under silicon treatment when attack from pathogenic fungi in plants. In this study, the expression profiles of all of the identified R2R3-*MYB* genes were down-regulated after silicon treatment. Our results provide a reference for further study of biotic stress under silicon treatment.

Most of the identified R2R3-*MYB* genes were clustered in the same group, indicating broad conservation of function of R2R3-*MYB* genes during wheat evolution. During evolution, gene duplication is frequently observed in plant genomes^40^. Among the 411 identified TaMYB TFs, 140, 131, and 139 were found in the A, B, and D sub-genomes, respectively. This result indicated that gene loss might have occurred in the wheat *MYB* gene family, resulting in the loss of some homoeologous copies. Furthermore, the fact that the number of TaMYB TFs was the least on sub-genome B suggested that initial gene loss may have reduced functional redundancy in the B sub-genome following tetraploidy.

## Materials and methods

### Plant materials and treatments

The “Kehua” cultivar of *T. aestivum* was grown in an artificial climate chamber at 26/22℃ (day/night) with a photoperiod of 16/8 h (day/night). The 2-week-old seedlings of wheat were subjected to two different treatments for 15 days: (1) non-Si-treated (S0): seedlings watered with Hoagland nutrient solution without soluble Si; (2) Si-treated (S1): seedlings watered with Hoagland nutrient solution containing 1 mM potassium silicate. For non-Si-treated cells, potassium chloride (1 mM) was used to replenish potassium. The wheat plants were irrigated with 30 mL of the corresponding solution (pH 6.5), and the Hoagland nutrient solution was changed every seven days. After treatment, whole seedlings under non-Si-treated and Si-treated plants were collected for RNA isolation and sequencing respectively. Three replicates were performed per treatment, and each replicate included 20 plants.

### RNA extraction, cDNA library construction, and Illumina sequencing

RNA isolation and cDNA synthesis were performed according to the manufacturer’ instructions (TIANGEN Biotech, Beijing). Six cDNA libraries were constructed from the mRNA of three independent samples of two treatments and sequenced on the Illumina Hiseq X platform. For RNA-seq, the OD260/280 of RNA range from 1.8 to 2.2, OD260/230 ≥ 2.0, RIN ≥ 6.5, and 28S:18S ≥ 1.0, and 5 μg RNA were used for RNA-seq. The detailed information of RNA is listed in Additional file 1. To ensure consistent gene transcription efficiency, the RNA concentration in each reverse transcription system was kept consistent (the 20 μl cDNA contained 1 μg total RNA) (Supplementary Figure S3 and Fig. S4).

### De novo assembly and function annotation

After removing primer and adaptor sequences from raw reads (raw reads with ambiguous bases ‘N’ (N > 10%), and low quality reads (more than 50% Q ≤ 5)), the clean reads were filtered. Then, the clean reads were assembled into contigs using Trinity de novo software and mapped to the reference genome of *T. aestivum* (IWGSC, http://plants.ensembl.org/Triticum_aestivum/Info/Index) using mapsplice software.

### Identification of differentially expressed genes (DEGs) and TFs

The expression level of each gene was calculated using the reads per kilobase per million mapped reads (RPKM method. All read counts were normalized to the RPKM value, representing the gene expression level. With a log-fold expression change of |log2FoldChange|> 2, and a threshold of false discovery rates (FDR ≤ 0.01), DEGs were filtered using a DEGseq algorithm. To obtain the TFs in DEGs, all DEGs were matched using BLAST against the Plant Transcription Factor Database (PlantTFDB v3.0)^41^ with an E-value cut-off of 10^–5^.

### Functional annotation and pathway analysis

WEGO software package^42^ was used to describe GO functional classification of biological processes, molecular functions, and cellular components. GO enrichment of DEGs was performed using the Singular Enrichment Analysis (SEA) method with P < 0.01 and a false discovery rate (FDR) < 0.05 by AgriGO^43^. We used the KEGG Orthology Based Annotation System software^44^ to determine the statistical enrichment of DEGs in the KEGG pathways with a threshold of significance of *P* value < 0.01 and FDR < 0.05.

### Real-time quantitative PCR validation

To determine the most stable reference genes, we used the Normfinder^45^, Bestkeeper^46^, and Delt Ct^47^ methods to determine the best one from eight candidates (Table S6). The qRT-PCR system and reaction condition followed by manufacturer’s instructions (TIANGEN Biotech, Beijing). Data collection and analysis were done using QuantStudio Real-Time PCR Software (ThermoFisher Scientific). The relative expression level was calculated using the 2^−ΔΔCt^ analysis method^47^. The primers used in this study were designed using OLIGO 7 software and are listed in Table S6.

### Identification of MYB TFs in wheat

To identify the MYB TFs in wheat, the HMM profile of MYB_DNA-binding domain PF00249 was downloaded from the Pfam database (http://pfam.xfam.org/) and searched against the protein sequences of wheat with a threshold of *E* < 1e^−5^ and amino acids > 200aa. The MYB protein sequences of 197 *Arabidopsis* and 155 rice MYB TFs were retrieved from the Ensembl Plant database (http://plants.ensembl.org/index.html), and the protein sequences of wheat were searched using a threshold of *E* < 1e^−5^ and an identify of 50%. Subsequently, a manual correction was performed to remove the alternative splicing events. The putative wheat MYB protein sequences were checked by SMART^48^ and NCBI CDD^49^ to confirm the presence of the MYB domain. Finally, to verify the existence of MYBs in wheat, we performed BLASTN to search for ESTs using the CDS of *TaMYB* in the NCBI database (https://www.ncbi.nlm.nih.gov/). The protein sequences, CDS, and cDNA sequences were downloaded from the Ensembl Plants database.

### Phylogenetic analysis and homoeologous identification

Multiple sequence alignments were performed using the T-Coffee method^50^, and the Neighbor Joining (NJ) tree was constructed using MEGA 7 software^51^ with the default parameters. The tree was visualized using Evolview^52^. The homoeologous genes were identified by phylogenetic analysis.

## Supplementary Information

Supplementary Information
