# Supplementary material for: Transcriptome analysis reveals differentially expressed MYB transcription factors associated with silicon response in wheat
Source: Sci Rep. 2021 Feb 22;11:4330. doi: 10.1038/s41598-021-83912-8 (PMC7900239; doi:10.1038/s41598-021-83912-8)
Supplement: Supplementary file 1 — Supplementary Information [file 41598_2021_83912_MOESM1_ESM.pdf]

Transcriptome analysis reveals differentially expressed MYB transcription factors associated with silicon response in wheat

Lidong Hao [1, 2], Shubing Shi [1*], Haibin Guo[2], Jinshan Zhang[1], Peng Li[1], Yanfei Feng[2]

1 College of Agriculture, Xinjiang Agricultural University, 311 Nongda East Road, Urumqi, 830052, China.;

2 College of Agriculture and Hydraulic Engineering, Sui Hua University, No.18, Huanghe Road, Suihua, 152061, China

***** Correspondence: Shubing Shi, haolidong1987@163.com, shubshi@126.com (S.S.), +86-991-8763824

Supplementary file 1 The quality and integrity of RNA

The concentration and purity of total RNA were checked by Qubit Fluorometer and NanoDrop 2000, and the RNA integrity were checked by Q-sep1 and agarose gel electrophoresis. As showed in Additional Table 1, all samples were met the standard of RNA-seq. And the checked results of RNA integrity were met the standard of RNA-seq.

Results of RNA quality and concentration

Sample name	concentration (ng/ μL)	volume (μL)	total RNA(μg)	A260/ 280	A260/ 230	RIN
A0-1	338	40	13.52	1.87	1.91	9.9
A0-2	366	40	14.64	1.89	1.98	9.89
A0-3	366	40	14.64	1.88	1.98	9.9
A1-1	272	40	10.88	1.87	1.92	9.0
A1-2	278	40	11.12	1.89	1.94	8.96
A1-3	368	40	14.72	1.87	1.92	8.9

Supplementary Figure S1 KEGG enrichment of DEGs

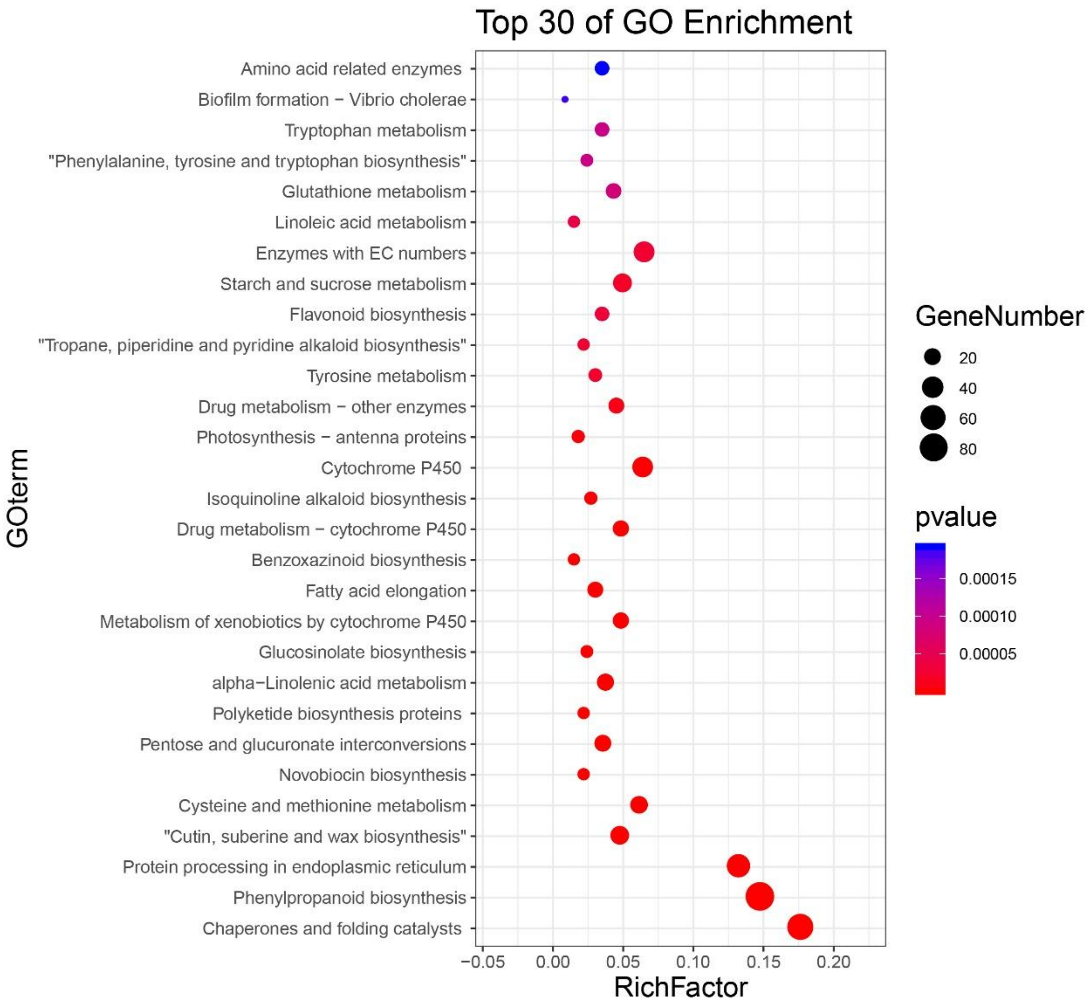

Supplementary Figure S2 Phylogenetic analysis of wheat R2R3-MYB TFs. A total of 15 groups (named S1 to S15) were classified based on the bootstrap values

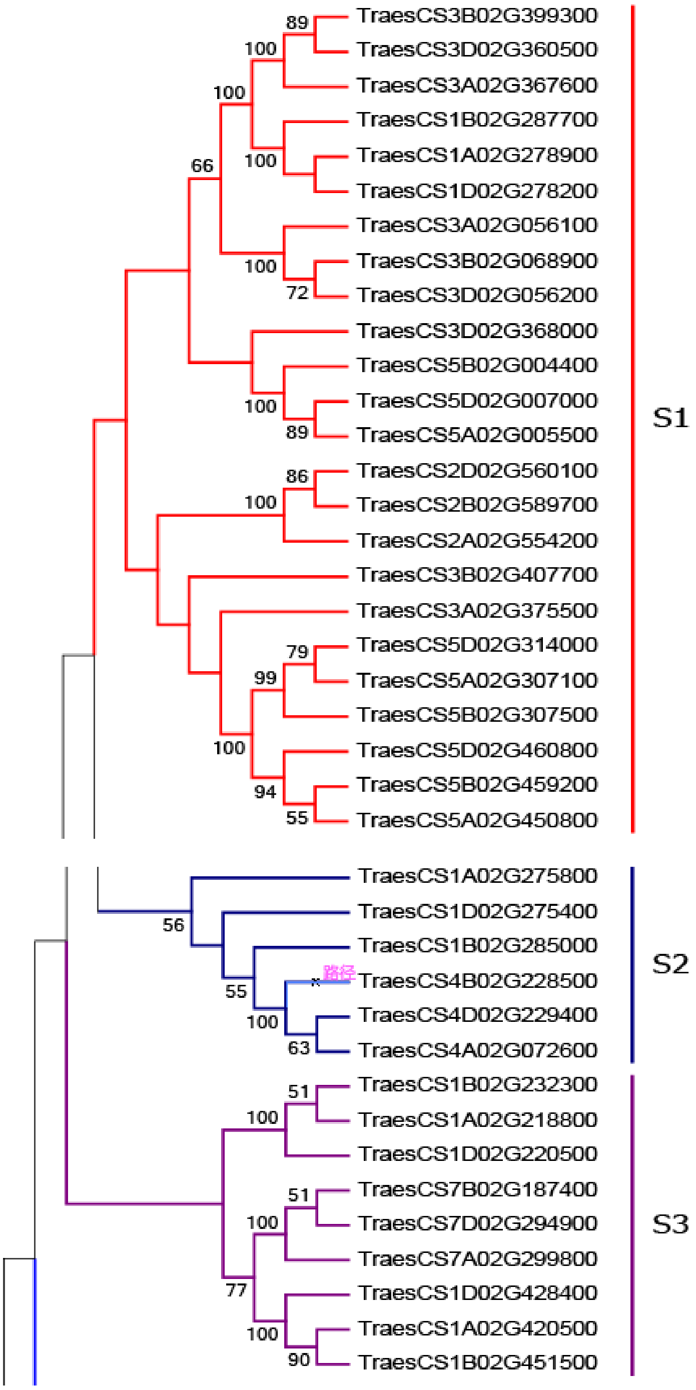

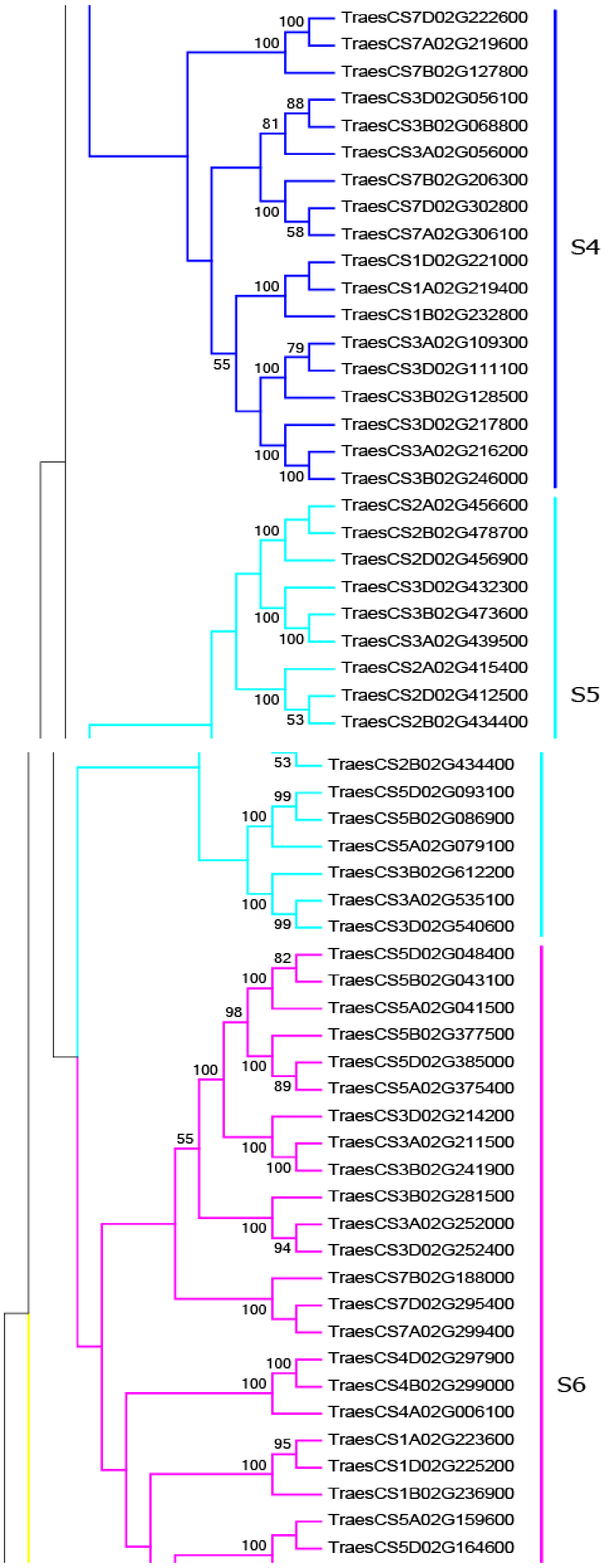
TraesCS7D02G222600
TraesCS7A02G219600
TraesCS7B02G127800
TraesCS3D02G056100
TraesCS3B02G068800
TraesCS3A02G056000
TraesCS7B02G206300
TraesCS7D02G302800
TraesCS7A02G306100
TraesCS1D02G221000
TraesCS1A02G219400
TraesCS1B02G232800
TraesCS3A02G109300
TraesCS3D02G111100
TraesCS3B02G128500
TraesCS3D02G217800
TraesCS3A02G216200
TraesCS3B02G246000
S4
TraesCS2A02G456600
TraesCS2B02G478700
TraesCS2D02G456900
TraesCS3D02G432300
TraesCS3B02G473600
TraesCS3A02G439500
TraesCS2A02G415400
TraesCS2D02G412500
TraesCS2B02G434400
S5
TraesCS2B02G434400
TraesCS5D02G093100
TraesCS5B02G086900
TraesCS5A02G079100
TraesCS3B02G612200
TraesCS3A02G535100
TraesCS3D02G540600
TraesCS5D02G048400
TraesCS5B02G043100
TraesCS5A02G041500
TraesCS5B02G377500
TraesCS5D02G385000
TraesCS5A02G375400
TraesCS3D02G214200
TraesCS3A02G211500
TraesCS3B02G241900
TraesCS3B02G281500
TraesCS3A02G252000
TraesCS3D02G252400
TraesCS7B02G188000
TraesCS7D02G295400
TraesCS7A02G299400
TraesCS4D02G297900
TraesCS4B02G299000
TraesCS4A02G006100
TraesCS1A02G223600
TraesCS1D02G225200
TraesCS1B02G236900
TraesCS5A02G159600
TraesCS5D02G164600
S6

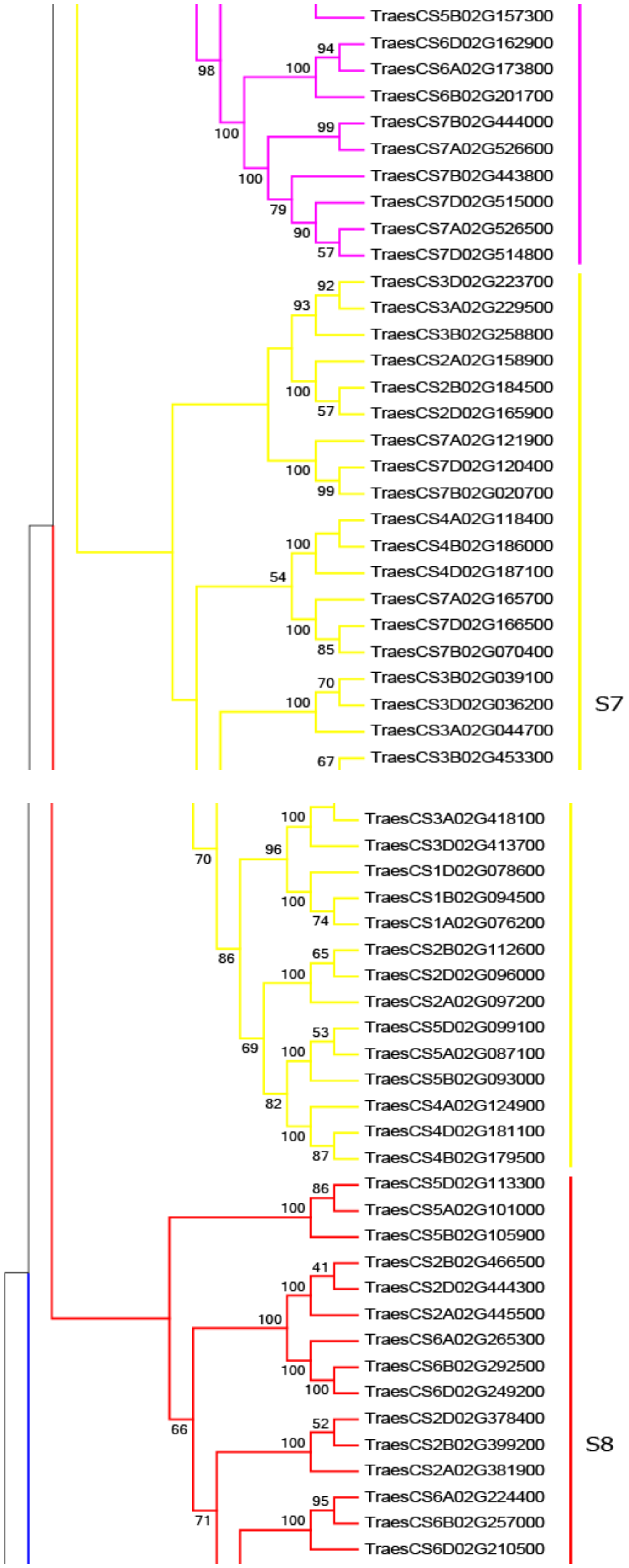

TraesCS5B02G157300
TraesCS6D02G162900
TraesCS6A02G173800
TraesCS6B02G201700
TraesCS7B02G444000
TraesCS7A02G526600
TraesCS7B02G443800
TraesCS7D02G515000
TraesCS7A02G526500
TraesCS7D02G514800
TraesCS3D02G223700
TraesCS3A02G229500
TraesCS3B02G258800
TraesCS2A02G158900
TraesCS2B02G184500
TraesCS2D02G165900
TraesCS7A02G121900
TraesCS7D02G120400
TraesCS7B02G020700
TraesCS4A02G118400
TraesCS4B02G186000
TraesCS4D02G187100
TraesCS7A02G165700
TraesCS7D02G166500
TraesCS7B02G070400
TraesCS3B02G039100
TraesCS3D02G036200
S7
TraesCS3A02G044700
TraesCS3B02G453300
TraesCS3A02G418100
TraesCS3D02G413700
TraesCS1D02G078600
TraesCS1B02G094500
TraesCS1A02G076200
TraesCS2B02G112600
TraesCS2D02G096000
TraesCS2A02G097200
TraesCS5D02G099100
TraesCS5A02G087100
TraesCS5B02G093000
TraesCS4A02G124900
TraesCS4D02G181100
TraesCS4B02G179500
TraesCS5D02G113300
TraesCS5A02G101000
TraesCS5B02G105900
TraesCS2B02G466500
TraesCS2D02G444300
TraesCS2A02G445500
TraesCS6A02G265300
TraesCS6B02G292500
TraesCS6D02G249200
TraesCS2D02G378400
TraesCS2B02G399200
S8
TraesCS2A02G381900
TraesCS6A02G224400
TraesCS6B02G257000
TraesCS6D02G210500

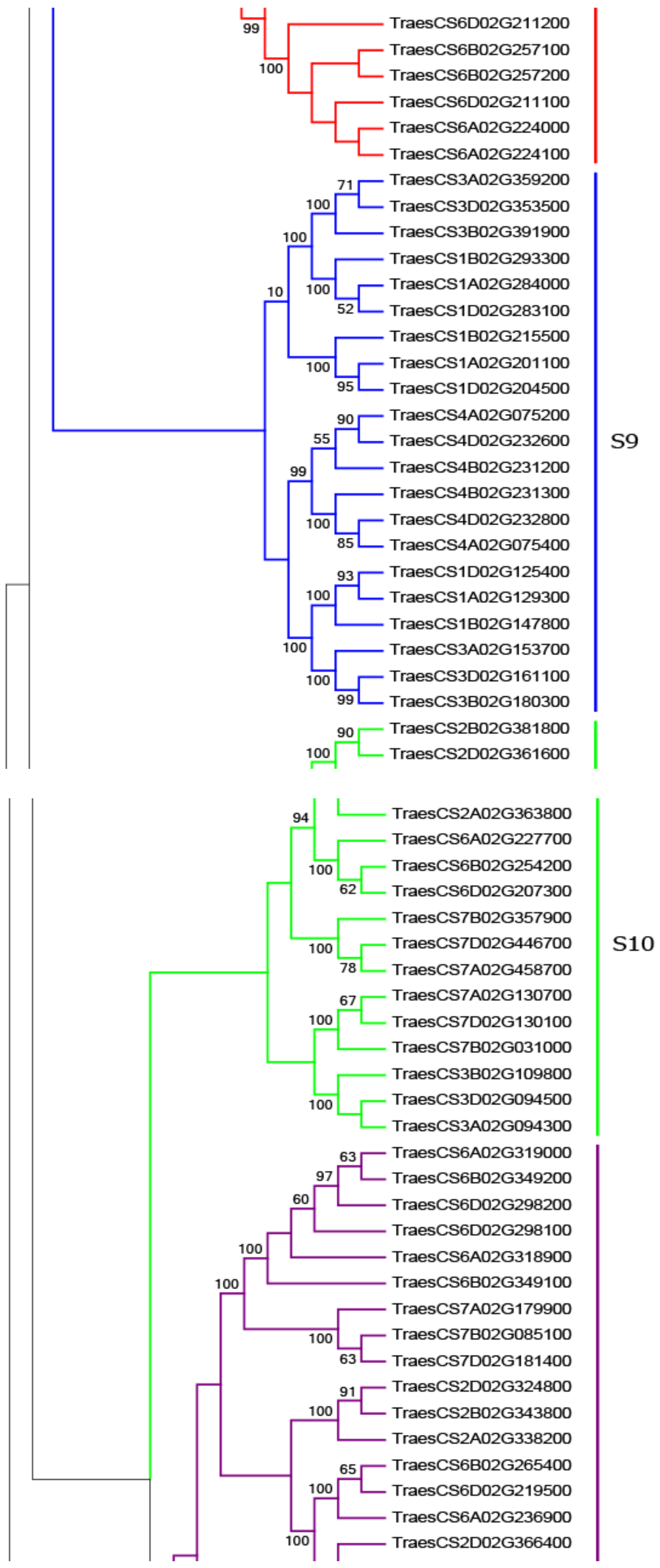
TraesCS6D02G211200
TraesCS6B02G257100
TraesCS6B02G257200
TraesCS6D02G211100
TraesCS6A02G224000
TraesCS6A02G224100
TraesCS3A02G359200
TraesCS3D02G353500
TraesCS3B02G391900
TraesCS1B02G293300
TraesCS1A02G284000
TraesCS1D02G283100
TraesCS1B02G215500
TraesCS1A02G201100
TraesCS1D02G204500
TraesCS4A02G075200
TraesCS4D02G232600
TraesCS4B02G231200
TraesCS4B02G231300
TraesCS4D02G232800
TraesCS4A02G075400
TraesCS1D02G125400
TraesCS1A02G129300
TraesCS1B02G147800
TraesCS3A02G153700
TraesCS3D02G161100
TraesCS3B02G180300
S9
TraesCS2B02G381800
TraesCS2D02G361600
TraesCS2A02G363800
TraesCS6A02G227700
TraesCS6B02G254200
TraesCS6D02G207300
TraesCS7B02G357900
TraesCS7D02G446700
TraesCS7A02G458700
TraesCS7A02G130700
TraesCS7D02G130100
TraesCS7B02G031000
TraesCS3B02G109800
TraesCS3D02G094500
TraesCS3A02G094300
S10
TraesCS6A02G319000
TraesCS6B02G349200
TraesCS6D02G298200
TraesCS6D02G298100
TraesCS6A02G318900
TraesCS6B02G349100
TraesCS7A02G179900
TraesCS7B02G085100
TraesCS7D02G181400
TraesCS2D02G324800
TraesCS2B02G343800
TraesCS2A02G338200
TraesCS6B02G265400
TraesCS6D02G219500
TraesCS6A02G236900
TraesCS2D02G366400

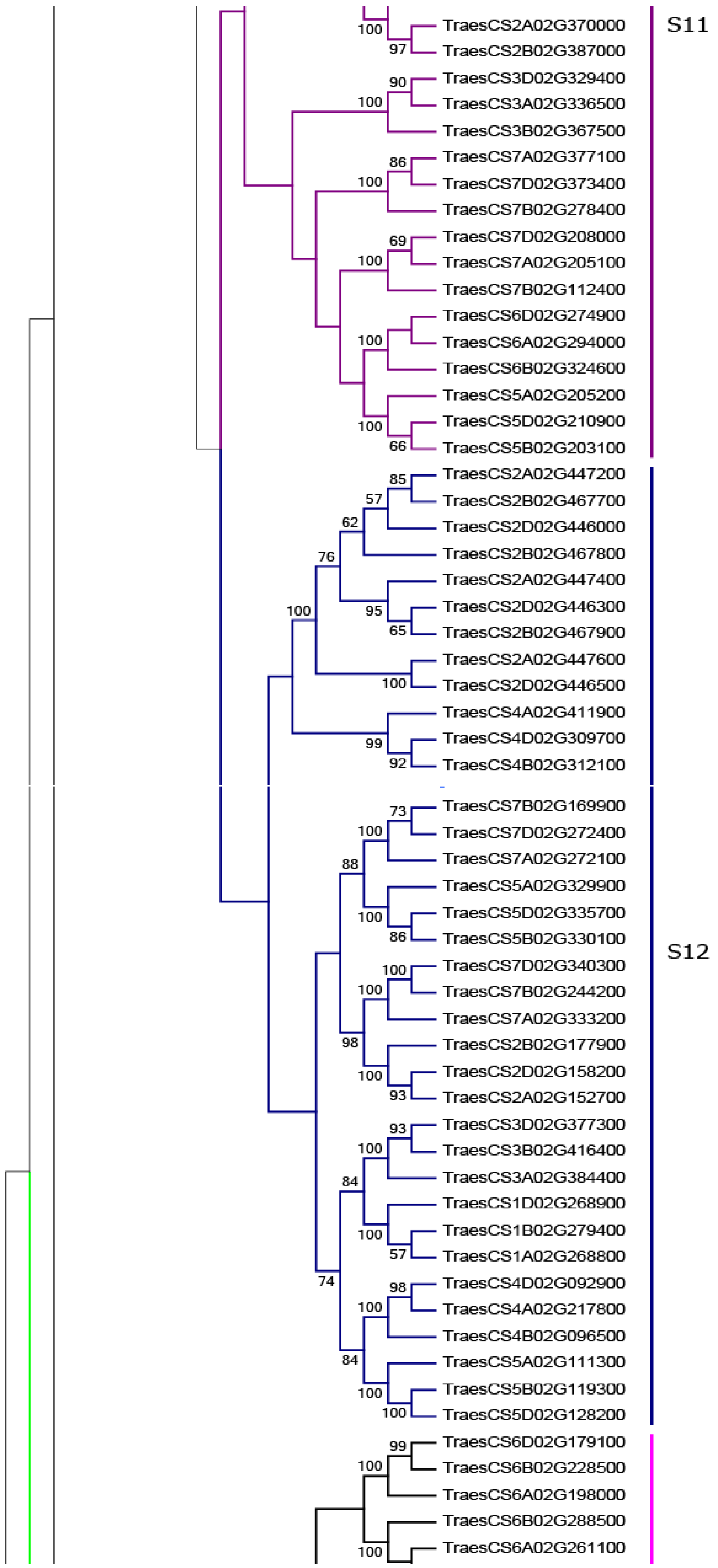
TraesCS2A02G370000
TraesCS2B02G387000
TraesCS3D02G329400
TraesCS3A02G336500
TraesCS3B02G367500
TraesCS7A02G377100
TraesCS7D02G373400
TraesCS7B02G278400
TraesCS7D02G208000
TraesCS7A02G205100
TraesCS7B02G112400
TraesCS6D02G274900
TraesCS6A02G294000
TraesCS6B02G324600
TraesCS5A02G205200
TraesCS5D02G210900
TraesCS5B02G203100
S11
TraesCS2A02G447200
TraesCS2B02G467700
TraesCS2D02G446000
TraesCS2B02G467800
TraesCS2A02G447400
TraesCS2D02G446300
TraesCS2B02G467900
TraesCS2A02G447600
TraesCS2D02G446500
TraesCS4A02G411900
TraesCS4D02G309700
TraesCS4B02G312100
TraesCS7B02G169900
TraesCS7D02G272400
TraesCS7A02G272100
TraesCS5A02G329900
TraesCS5D02G335700
TraesCS5B02G330100
TraesCS7D02G340300
TraesCS7B02G244200
TraesCS7A02G333200
TraesCS2B02G177900
TraesCS2D02G158200
TraesCS2A02G152700
TraesCS3D02G377300
TraesCS3B02G416400
TraesCS3A02G384400
TraesCS1D02G268900
TraesCS1B02G279400
TraesCS1A02G268800
TraesCS4D02G092900
TraesCS4A02G217800
TraesCS4B02G096500
TraesCS5A02G111300
TraesCS5B02G119300
TraesCS5D02G128200
S12
TraesCS6D02G179100
TraesCS6B02G228500
TraesCS6A02G198000
TraesCS6B02G288500
TraesCS6A02G261100

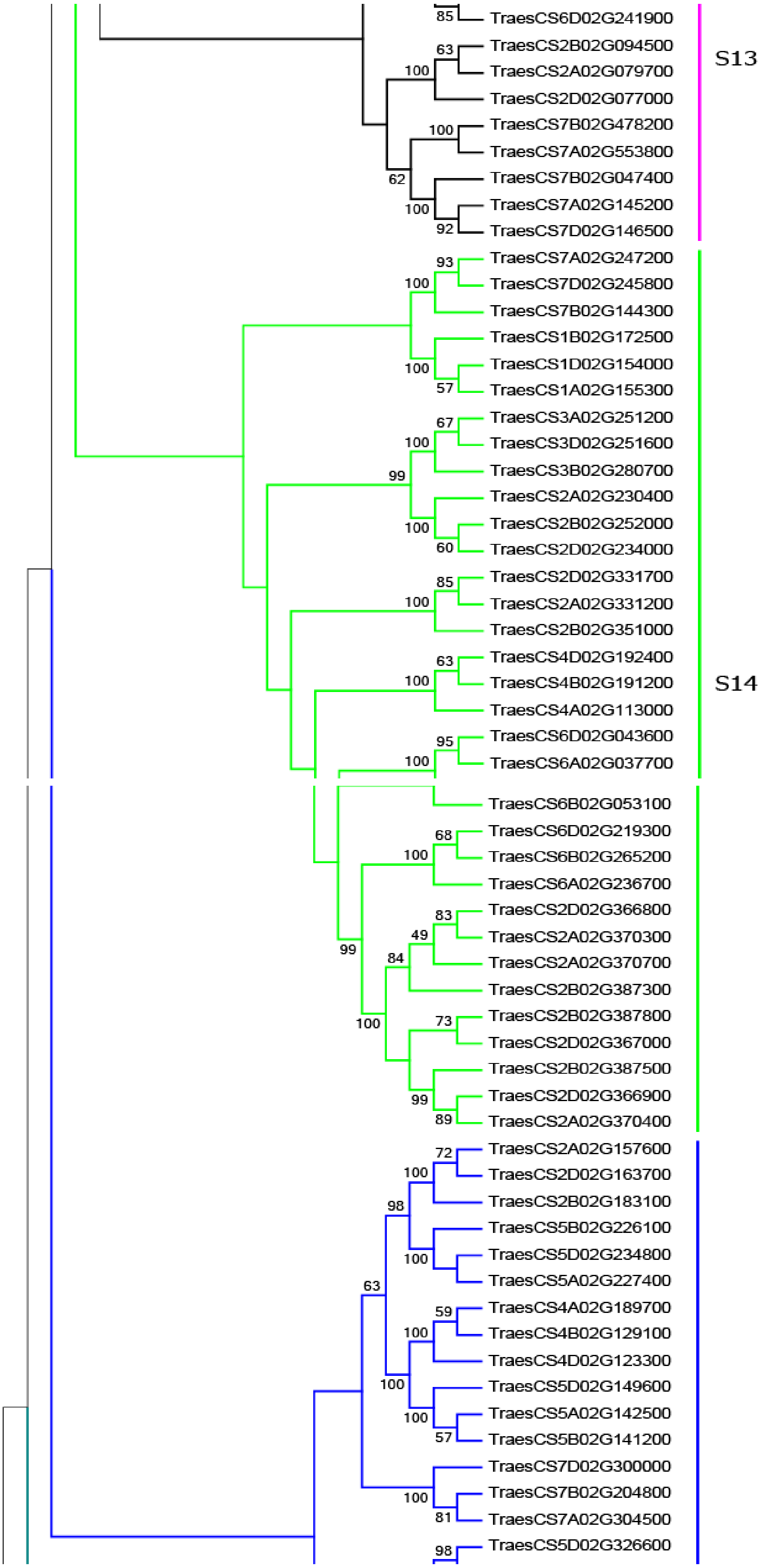

TraesCS6D02G241900
TraesCS2B02G094500
TraesCS2A02G079700
TraesCS2D02G077000
TraesCS7B02G478200
TraesCS7A02G553800
TraesCS7B02G047400
TraesCS7A02G145200
TraesCS7D02G146500
S13
TraesCS7A02G247200
TraesCS7D02G245800
TraesCS7B02G144300
TraesCS1B02G172500
TraesCS1D02G154000
TraesCS1A02G155300
TraesCS3A02G251200
TraesCS3D02G251600
TraesCS3B02G280700
TraesCS2A02G230400
TraesCS2B02G252000
TraesCS2D02G234000
TraesCS2D02G331700
TraesCS2A02G331200
TraesCS2B02G351000
TraesCS4D02G192400
TraesCS4B02G191200
TraesCS4A02G113000
TraesCS6D02G043600
TraesCS6A02G037700
S14
TraesCS6B02G053100
TraesCS6D02G219300
TraesCS6B02G265200
TraesCS6A02G236700
TraesCS2D02G366800
TraesCS2A02G370300
TraesCS2A02G370700
TraesCS2B02G387300
TraesCS2B02G387800
TraesCS2D02G367000
TraesCS2B02G387500
TraesCS2D02G366900
TraesCS2A02G370400
TraesCS2A02G157600
TraesCS2D02G163700
TraesCS2B02G183100
TraesCS5B02G226100
TraesCS5D02G234800
TraesCS5A02G227400
TraesCS4A02G189700
TraesCS4B02G129100
TraesCS4D02G123300
TraesCS5D02G149600
TraesCS5A02G142500
TraesCS5B02G141200
TraesCS7D02G300000
TraesCS7B02G204800
TraesCS7A02G304500
TraesCS5D02G326600

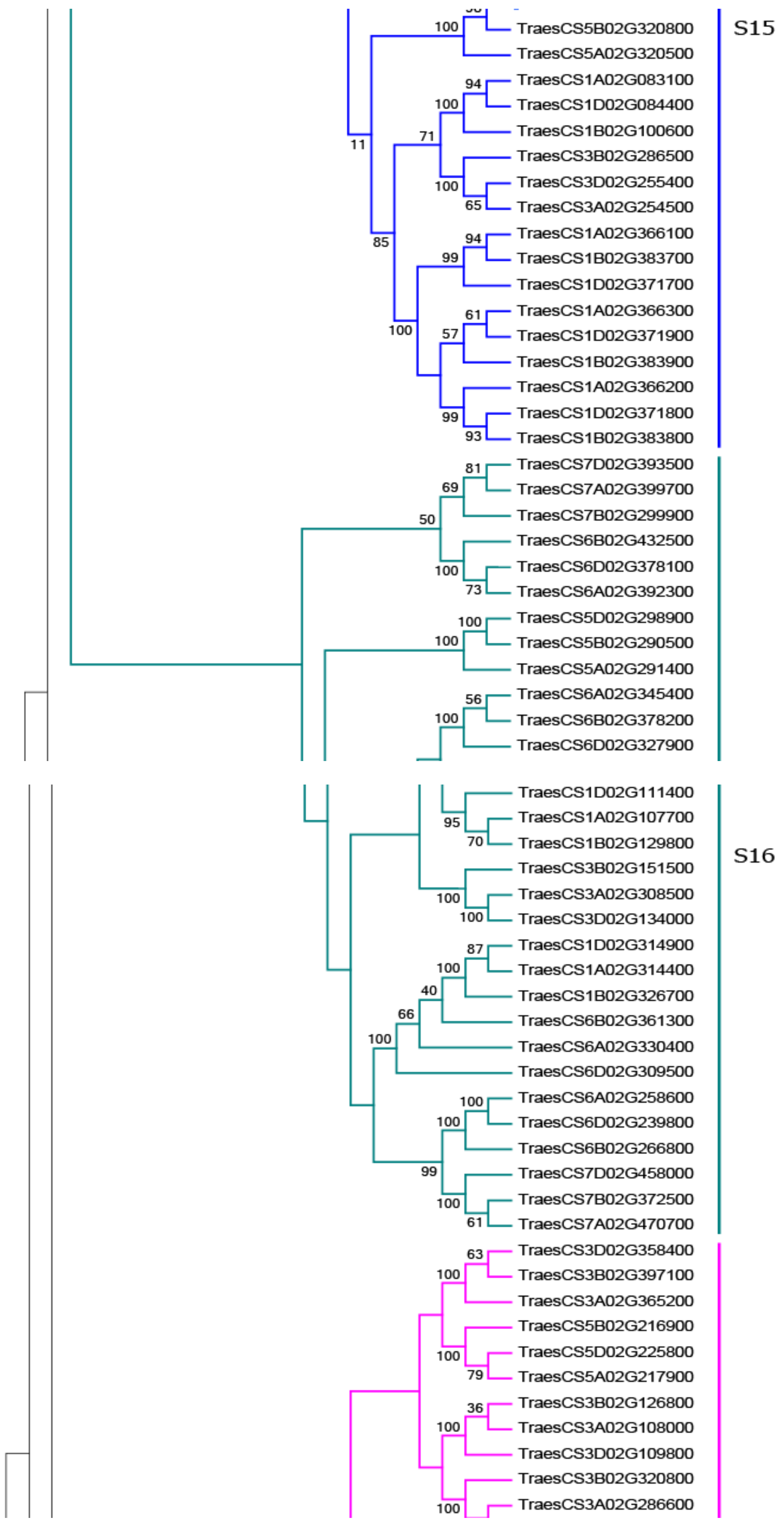

S15
TraesCS5B02G320800
TraesCS5A02G320500
TraesCS1A02G083100
TraesCS1D02G084400
TraesCS1B02G100600
TraesCS3B02G286500
TraesCS3D02G255400
TraesCS3A02G254500
TraesCS1A02G366100
TraesCS1B02G383700
TraesCS1D02G371700
TraesCS1A02G366300
TraesCS1D02G371900
TraesCS1B02G383900
TraesCS1A02G366200
TraesCS1D02G371800
TraesCS1B02G383800
TraesCS7D02G393500
TraesCS7A02G399700
TraesCS7B02G299900
TraesCS6B02G432500
TraesCS6D02G378100
TraesCS6A02G392300
TraesCS5D02G298900
TraesCS5B02G290500
TraesCS5A02G291400
TraesCS6A02G345400
TraesCS6B02G378200
TraesCS6D02G327900
TraesCS1D02G111400
TraesCS1A02G107700
TraesCS1B02G129800
S16
TraesCS3B02G151500
TraesCS3A02G308500
TraesCS3D02G134000
TraesCS1D02G314900
TraesCS1A02G314400
TraesCS1B02G326700
TraesCS6B02G361300
TraesCS6A02G330400
TraesCS6D02G309500
TraesCS6A02G258600
TraesCS6D02G239800
TraesCS6B02G266800
TraesCS7D02G458000
TraesCS7B02G372500
TraesCS7A02G470700
TraesCS3D02G358400
TraesCS3B02G397100
TraesCS3A02G365200
TraesCS5B02G216900
TraesCS5D02G225800
TraesCS5A02G217900
TraesCS3B02G126800
TraesCS3A02G108000
TraesCS3D02G109800
TraesCS3B02G320800
TraesCS3A02G286600

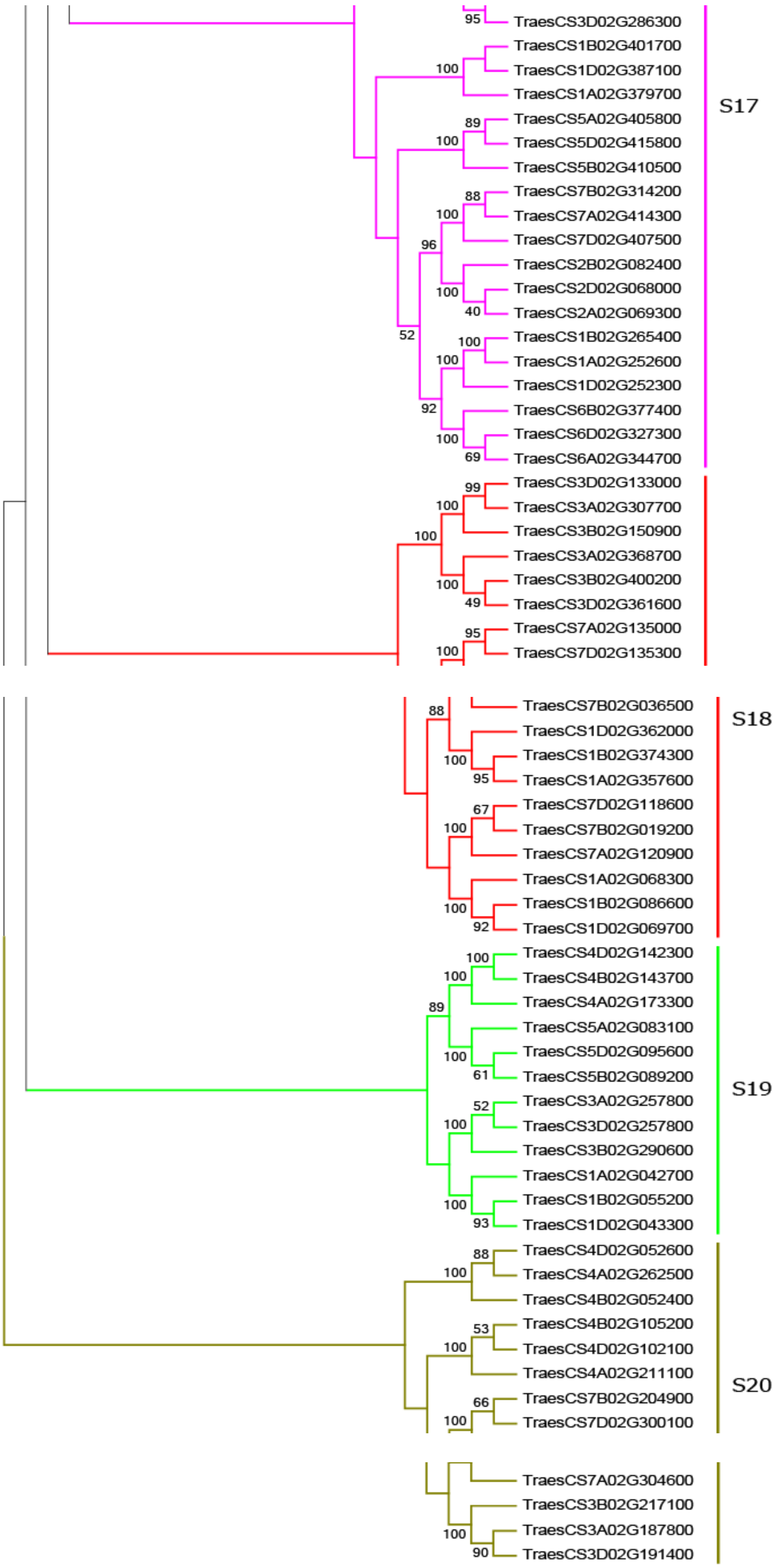
TraesCS3D02G286300
TraesCS1B02G401700
TraesCS1D02G387100
TraesCS1A02G379700
S17
TraesCS5A02G405800
TraesCS5D02G415800
TraesCS5B02G410500
TraesCS7B02G314200
TraesCS7A02G414300
TraesCS7D02G407500
TraesCS2B02G082400
TraesCS2D02G068000
TraesCS2A02G069300
TraesCS1B02G265400
TraesCS1A02G252600
TraesCS1D02G252300
TraesCS6B02G377400
TraesCS6D02G327300
TraesCS6A02G344700
TraesCS3D02G133000
TraesCS3A02G307700
TraesCS3B02G150900
TraesCS3A02G368700
TraesCS3B02G400200
TraesCS3D02G361600
TraesCS7A02G135000
TraesCS7D02G135300
TraesCS7B02G036500
S18
TraesCS1D02G362000
TraesCS1B02G374300
TraesCS1A02G357600
TraesCS7D02G118600
TraesCS7B02G019200
TraesCS7A02G120900
TraesCS1A02G068300
TraesCS1B02G086600
TraesCS1D02G069700
TraesCS4D02G142300
TraesCS4B02G143700
TraesCS4A02G173300
TraesCS5A02G083100
TraesCS5D02G095600
TraesCS5B02G089200
S19
TraesCS3A02G257800
TraesCS3D02G257800
TraesCS3B02G290600
TraesCS1A02G042700
TraesCS1B02G055200
TraesCS1D02G043300
TraesCS4D02G052600
TraesCS4A02G262500
TraesCS4B02G052400
TraesCS4B02G105200
TraesCS4D02G102100
TraesCS4A02G211100
S20
TraesCS7B02G204900
TraesCS7D02G300100
TraesCS7A02G304600
TraesCS3B02G217100
TraesCS3A02G187800
TraesCS3D02G191400

Supplementary Figure S3 Agarose gel electrophoresis results

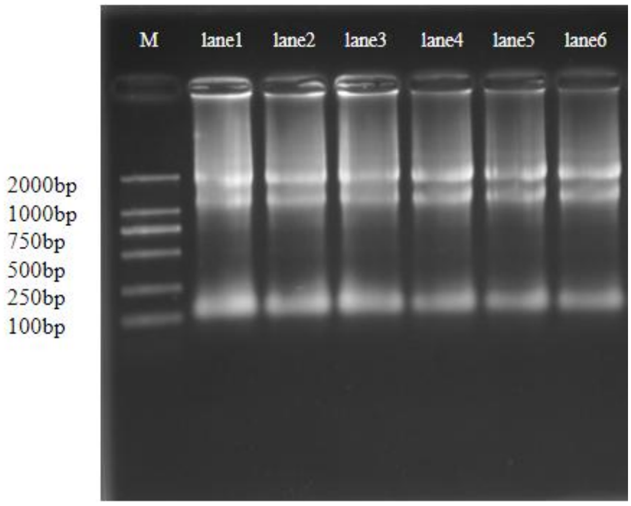

（M:DL2000,lane1:S0-1;lane2:S0-2;lane3:S0-3;lane4:S1-1;lane5:S1-2;lane6:S1-3）

Supplementary Figure S4 RNA integrity were checked by Q-sep1

1）S0-1

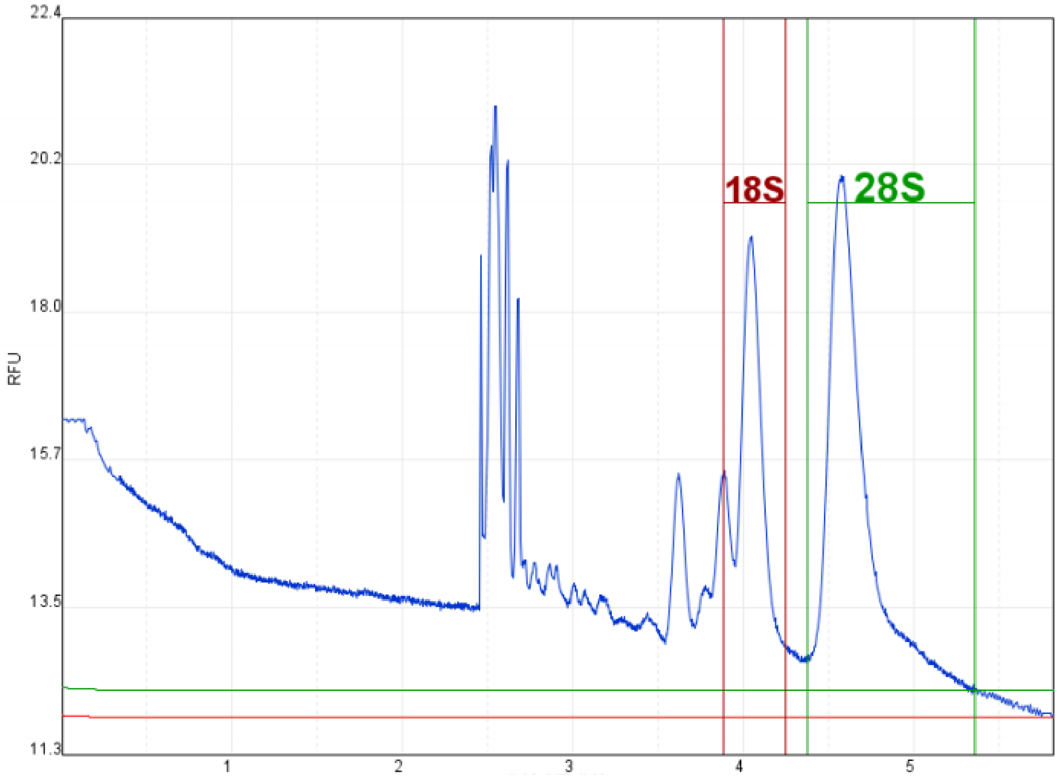

2）S0-2

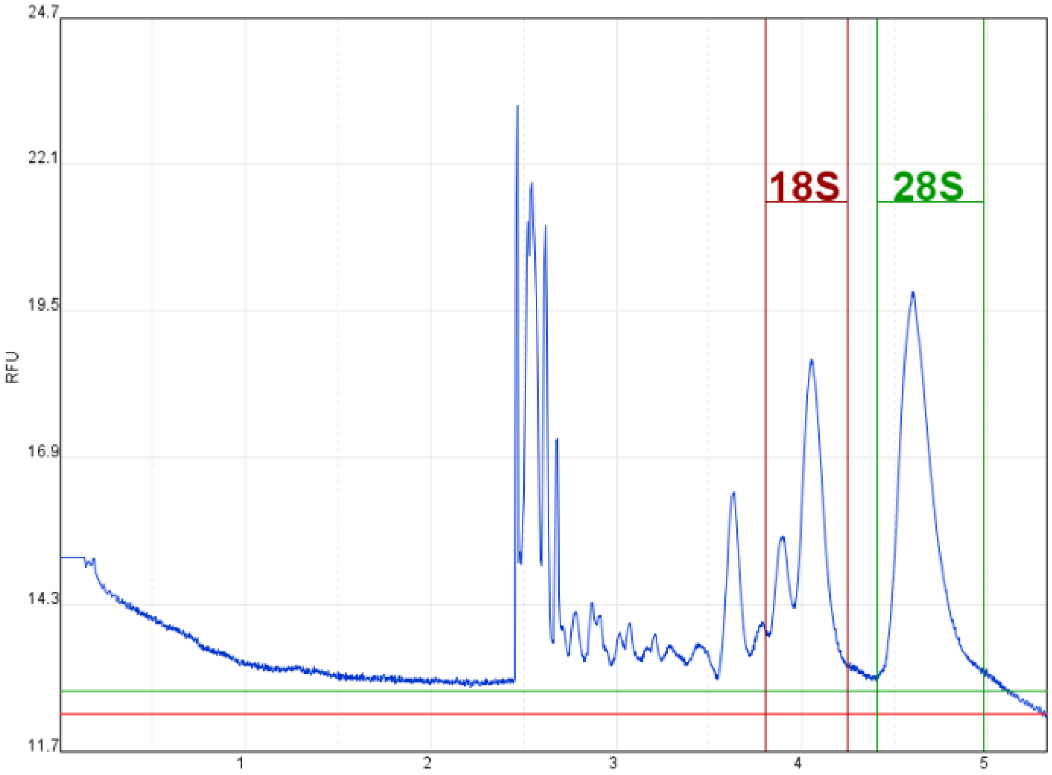

3）S0-3

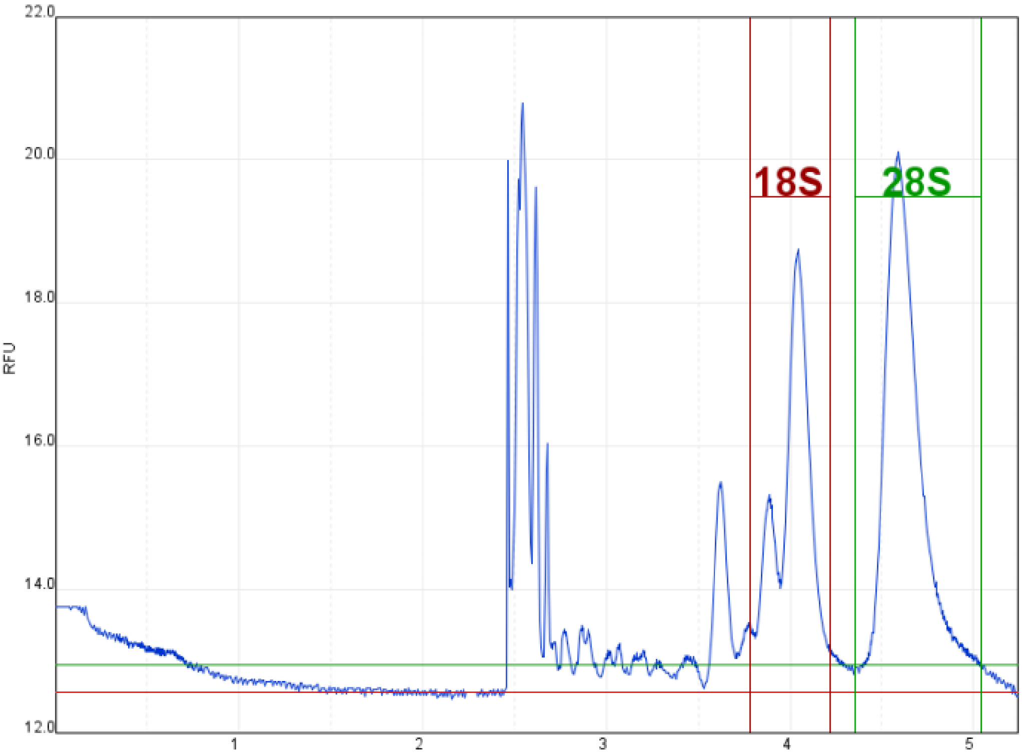

4）S1-1

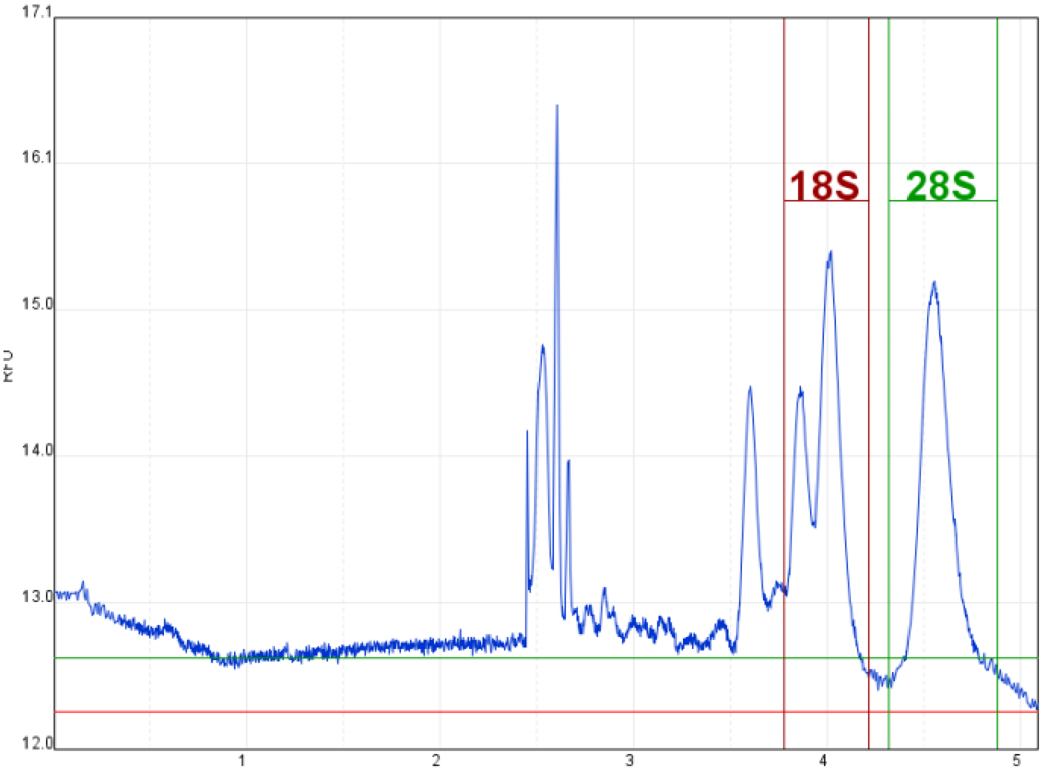

5）S1-2

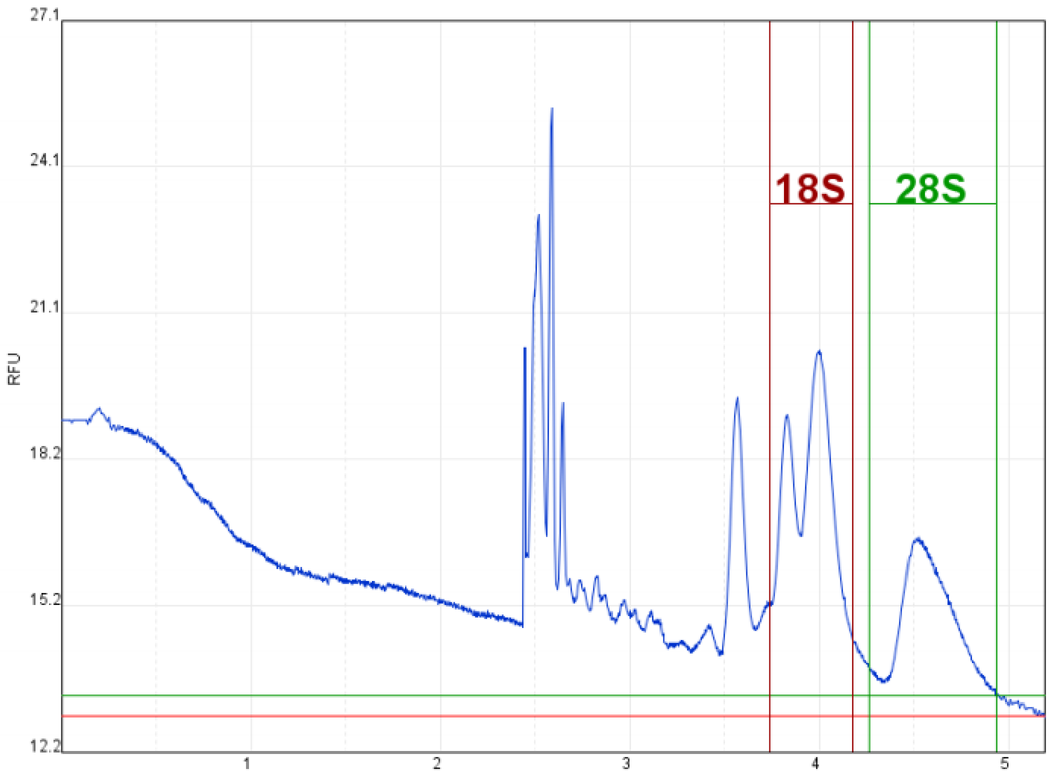
27.1
24.1
21.1
18.2
15.2
12.2
RFU
18S
28S
1
2
3
4
5

6）S1-3

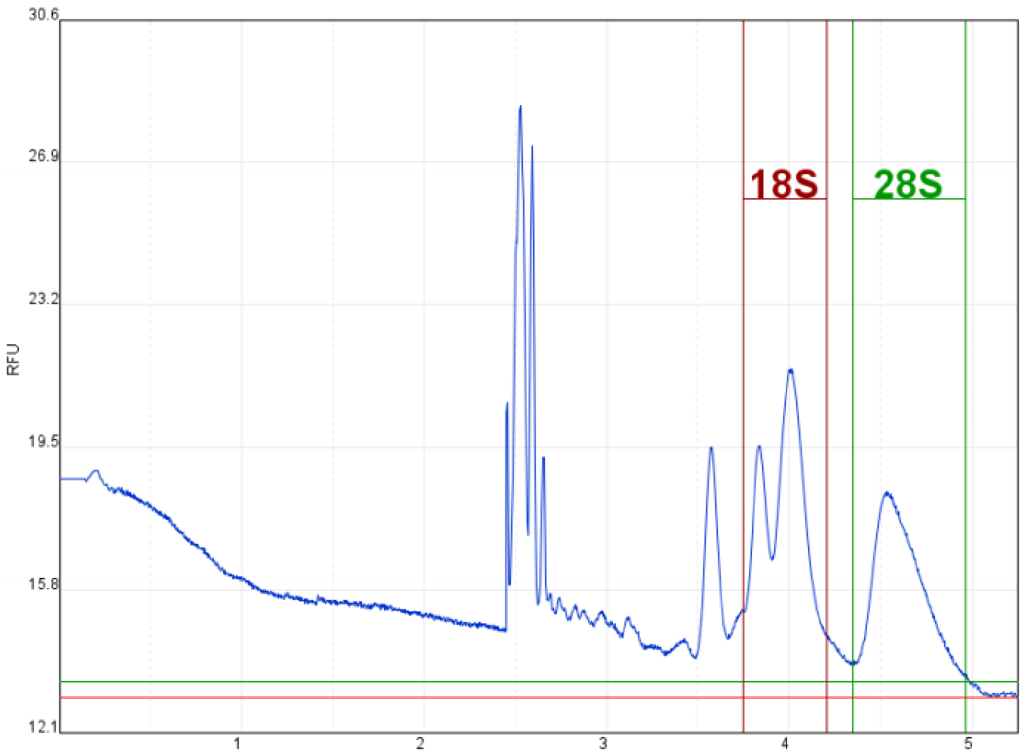
30.6
26.9
23.2
19.5
15.8
12.1
RFU
18S
28S
1
2
3
4
5

Supplementary Figure S5 Primer efficiency was measured using four- to eightfold dilutions of the cDNA stock.

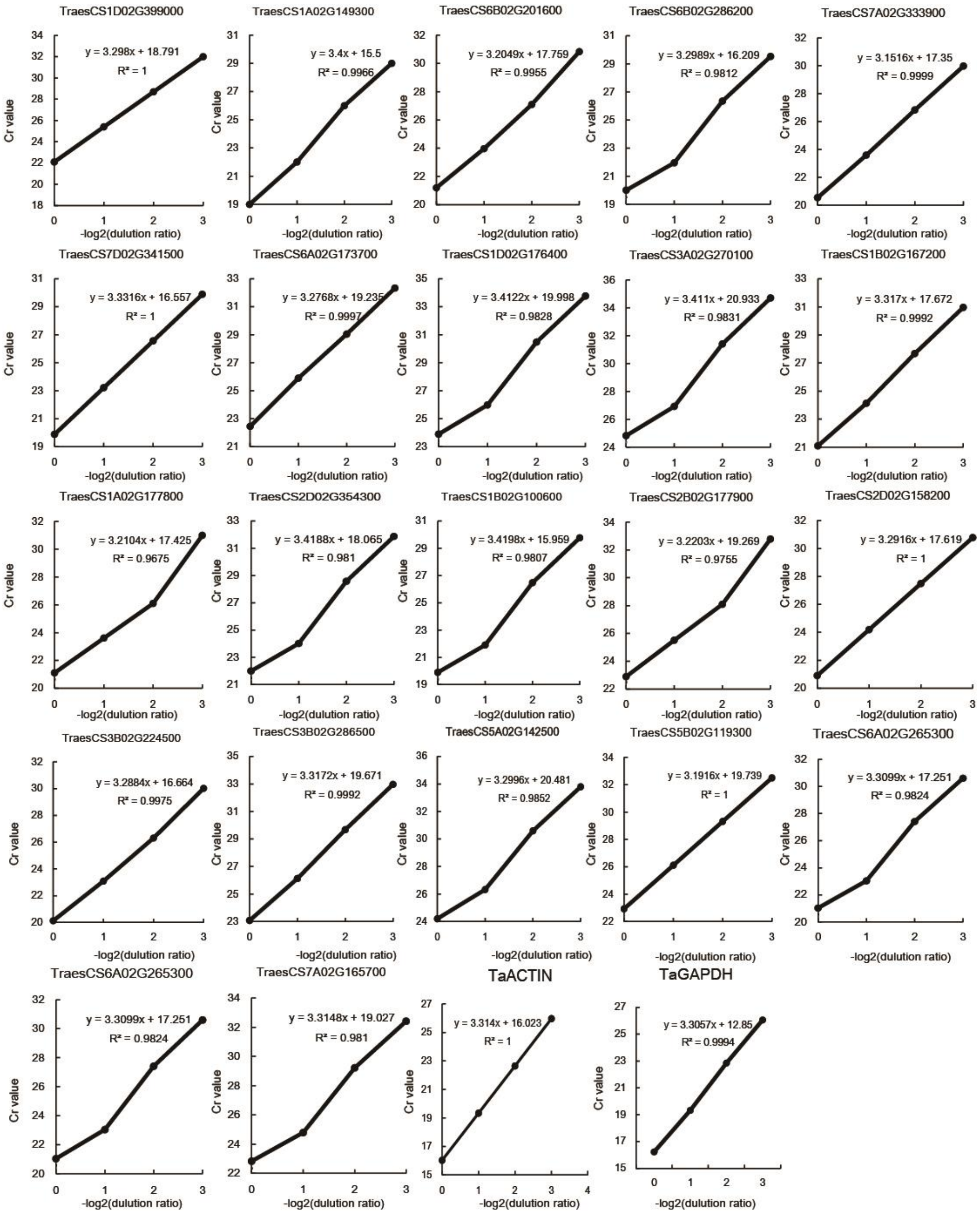

Curves show log2 values for the dilution ratio plotted against Ct values from qRT-PCR amplification using the different primer pairs.

Supplementary Figure S6 Comparative expression analysis of six genes during silicon treatments.

Expression patterns were constructed by RT-qPCR with *ACTIN* (**a**) or *GAPDH* (**c**) as an internal control, and basing on RNA-Seq data (**b**)

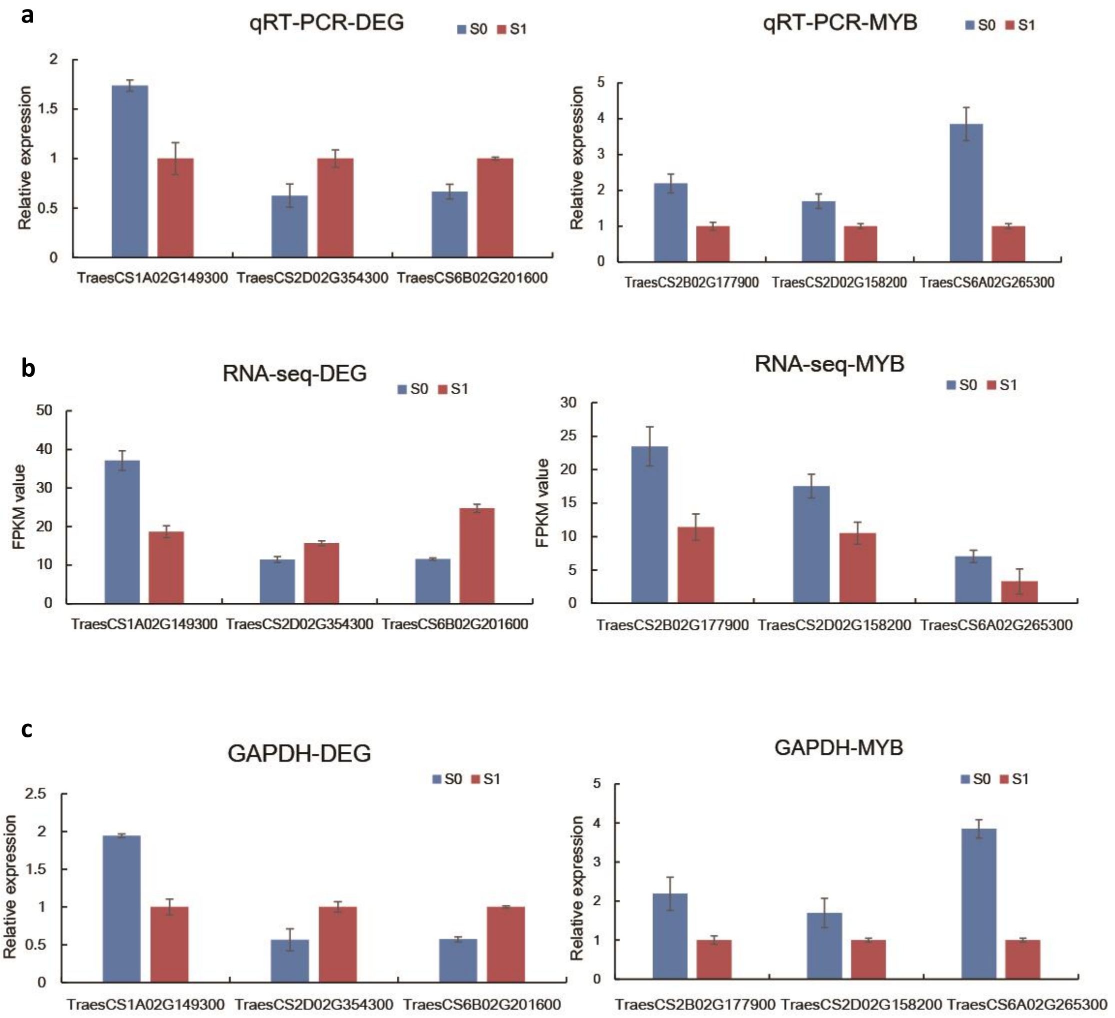

Supplementary Table S1_Quality evaluation of RNA-seq data

Sample	RawReads	CleanReads	CleanBases	CleanRatio	Q20	Q30	GC
S0_1	54141484	54109138	8083587115	99.94%	98.99%	97.02%	57.77%
S0_2	57029986	56982420	8507492358	99.92%	98.95%	96.98%	57.54%
S0_3	65470048	65419430	9765062141	99.92%	98.94%	96.91%	56.61%
S1_1	63416816	63341970	9448250580	99.88%	98.68%	96.42%	58.26%
S1_2	61421932	61360966	9151509510	99.90%	98.80%	96.71%	58.35%
S1_3	46264840	46223432	6892776915	99.91%	98.78%	96.62%	56.80%
Total	347745106	3.47E+08	51848678619	99.78%			

Supplementary Table S2 Differentially expressed genes under silicon treatment

Gene	logFC	logCPM	PValue	FDR
TraesCS1D02G289700	7.8699	5.9032	1.03E-242	4.37E-238
TraesCS1B02G300500	6.8251	5.9832	1.43E-240	3.04E-236
TraesCS4B02G183900	6.0921	6.1615	2.36E-212	3.34E-208
TraesCS6D02G370800	6.4678	6.0737	7.15E-206	7.60E-202
TraesCS6D02G148600	6.7677	6.0517	8.63E-200	7.33E-196
TraesCS6A02G093000	6.9865	5.5681	1.54E-198	1.09E-194
TraesCS6A02G163100	6.4758	5.9132	1.58E-197	9.60E-194
TraesCS6B02G186000	6.3613	6.2578	8.44E-197	4.48E-193
TraesCS6B02G186100	6.3671	6.237	6.11E-195	2.89E-191
TraesCS6D02G148200	5.7469	6.1534	4.78E-180	2.03E-176
TraesCS4B02G131400	5.9895	5.3608	5.66E-176	2.19E-172
TraesCS4D02G125900	6.4462	4.9647	3.24E-174	1.15E-170
TraesCS1A02G291100	5.7357	5.1869	7.48E-173	2.45E-169
TraesCS6D02G148000	7.857	5.8944	4.39E-169	1.33E-165
TraesCS2D02G049200	7.1165	4.7266	1.45E-150	4.12E-147
TraesCS6A02G165200	4.7359	6.5318	4.02E-144	1.07E-140
TraesCS2A02G049900	6.1969	4.7238	9.53E-144	2.38E-140
TraesCS6A02G165100	4.7224	6.4973	4.91E-139	1.16E-135
TraesCS4D02G325600	7.4909	4.2623	2.81E-137	6.27E-134
TraesCS6B02G187400	6.042	5.8406	6.79E-132	1.44E-128
TraesCS5D02G000100	11.0908	3.7888	2.98E-130	6.03E-127
TraesCS4A02G187500	7.1631	3.9398	3.38E-129	6.53E-126
TraesCSU02G127700	5.8358	4.4927	6.80E-124	1.26E-120
TraesCS1A02G165400	4.2771	4.5571	4.04E-108	7.16E-105
TraesCS6A02G163200	5.9883	4.0452	1.92E-101	3.26E-98
TraesCS3A02G110100	-4.6478	4.8376	1.04E-97	1.70E-94
TraesCS4A02G074800	3.2354	5.6065	1.81E-95	2.85E-92
TraesCS5B02G015500	3.8306	5.2141	2.26E-95	3.43E-92
TraesCS7A02G259100	3.6946	5.1832	4.82E-93	7.07E-90
TraesCS3B02G068500	3.5926	6.9428	2.36E-91	3.34E-88
TraesCS4D02G184900	4.2489	6.4395	7.57E-90	1.04E-86
TraesCS5A02G017900	3.2972	5.6774	1.80E-85	2.39E-82
TraesCS1A02G094700	-3.1934	4.9358	2.67E-75	3.44E-72
TraesCS1A02G268400	-2.5472	6.4353	1.28E-73	1.60E-70
TraesCSU02G125500	4.8333	4.0037	9.20E-73	1.12E-69
TraesCS3B02G129200	-4.6054	3.5404	9.76E-73	1.15E-69
TraesCSU02G047700	-4.124	3.4935	1.52E-71	1.74E-68
TraesCS2B02G060800	10.3964	3.1122	3.94E-71	4.40E-68
TraesCS3D02G112000	-3.1643	5.317	2.74E-68	2.99E-65
TraesCS6D02G136200	-7.8583	2.4988	8.94E-66	9.49E-63
TraesCS7B02G184800	9.6214	2.3507	2.26E-65	2.34E-62
TraesCS7B02G130600	2.4022	6.1279	5.38E-64	5.44E-61
TraesCS7D02G232300	2.4377	5.4115	3.42E-62	3.38E-59
TraesCS1D02G103200	-4.0506	3.387	9.93E-62	9.59E-59
TraesCS3A02G102500	-9.6226	2.3736	1.37E-61	1.29E-58
TraesCS7B02G253700	2.5221	5.9342	2.24E-61	2.07E-58

TraesCS5D02G484900	4.2658	3.2706	8.52E-61	7.71E-58
TraesCS3B02G004600	-4.0137	3.5841	3.84E-59	3.40E-56
TraesCS3B02G068400	6.9433	2.3306	1.72E-56	1.49E-53
TraesCS1A02G280700	2.3794	6.785	1.10E-54	9.34E-52
TraesCS4D02G232200	2.4785	5.5106	2.26E-54	1.88E-51
TraesCS5D02G464900	-3.4648	3.4816	2.07E-53	1.69E-50
TraesCS3D02G260500	2.244	6.4293	3.38E-51	2.71E-48
TraesCS4D02G212500	6.7003	2.0997	8.95E-51	7.04E-48
TraesCS5D02G446800	2.2153	5.8247	8.11E-50	6.26E-47
TraesCS3A02G260100	2.3757	5.8641	1.15E-49	8.75E-47
TraesCS4D02G001600	-9.0869	1.8642	3.20E-49	2.39E-46
TraesCS6A02G279400	2.2114	5.6288	4.62E-49	3.38E-46
TraesCS5B02G006500	-2.2744	6.0302	6.49E-49	4.67E-46
TraesCSU02G158800	-7.3157	1.98	8.64E-49	6.12E-46
TraesCS1D02G452300	-2.9389	4.3599	1.12E-48	7.83E-46
TraesCS2D02G018600	-3.6326	3.005	4.69E-48	3.21E-45
TraesCSU02G047600	-2.584	3.9934	5.55E-48	3.74E-45
TraesCS4B02G225400	4.6499	2.5704	1.04E-45	6.89E-43
TraesCS3B02G266000	1.7284	6.8324	1.05E-44	6.83E-42
TraesCS4A02G092700	8.9061	1.6685	1.75E-44	1.12E-41
TraesCS3A02G260200	2.0752	6.3331	1.97E-44	1.25E-41
TraesCS3A02G034500	5.2167	2.0646	1.23E-43	7.70E-41
TraesCS1D02G411600	-3.3413	2.8957	2.37E-43	1.46E-40
TraesCS4D02G226000	5.3873	1.9901	3.06E-43	1.86E-40
TraesCS3D02G047700	-2.5332	3.6251	7.49E-43	4.48E-40
TraesCS7B02G253600	1.8034	6.673	6.41E-42	3.78E-39
TraesCS2D02G275900	3.8761	2.4447	1.70E-41	9.88E-39
TraesCS1D02G341700	1.6956	6.1782	1.87E-41	1.07E-38
TraesCS1A02G040900	-4.1745	2.6235	3.38E-41	1.91E-38
TraesCS6D02G259800	2.117	4.9647	1.37E-40	7.67E-38
TraesCS6D02G121900	-2.6591	5.4782	1.45E-40	7.97E-38
TraesCS6A02G249300	-2.0733	4.5656	2.03E-40	1.09E-37
TraesCS3D02G045800	5.796	1.7318	2.03E-40	1.09E-37
TraesCS4D02G056700	2.0595	7.062	1.74E-39	9.24E-37
TraesCSU02G073000	-8.7845	1.5803	2.64E-39	1.38E-36
TraesCS6D02G013600	-8.7612	1.5579	2.77E-39	1.44E-36
TraesCS5B02G260600	-9.1004	1.8741	7.04E-39	3.61E-36
TraesCS3B02G012100	-9.5401	2.2972	9.96E-39	5.04E-36
TraesCS1B02G170200	-2.061	5.7354	2.73E-37	1.36E-34
TraesCS3A02G033900	5.3969	1.7181	3.46E-37	1.71E-34
TraesCS4D02G314400	1.7907	5.4469	5.02E-37	2.45E-34
TraesCS4D02G122600	1.7235	5.2606	4.47E-36	2.16E-33
TraesCS1D02G289800	2.1698	5.4125	5.97E-36	2.85E-33
TraesCS1D02G042300	-3.9099	2.0453	2.28E-35	1.08E-32
TraesCS6D02G026300	-2.5679	3.5185	3.13E-35	1.46E-32
TraesCS7D02G324000	2.9031	2.6412	3.62E-35	1.67E-32
TraesCS3B02G293200	1.8756	6.8903	4.23E-35	1.93E-32
TraesCS4A02G092600	6.1025	1.5402	1.04E-34	4.72E-32

TraesCS1A02G306600	-1.8961	5.634	1.24E-34	5.53E-32
TraesCS2D02G272400	-2.3938	3.6074	1.30E-34	5.74E-32
TraesCS7B02G227900	1.9284	5.0521	2.53E-34	1.11E-31
TraesCS5A02G236100	1.4747	6.9498	9.29E-34	4.03E-31
TraesCS2D02G217200	-2.3018	3.5917	3.03E-33	1.30E-30
TraesCS1B02G115900	-1.5941	6.9575	5.32E-33	2.26E-30
TraesCS6D02G122000	-2.0708	4.6766	1.03E-32	4.33E-30
TraesCS3D02G045500	2.5478	3.4419	1.05E-32	4.36E-30
TraesCS2D02G195800	-2.0521	4.0311	1.10E-32	4.53E-30
TraesCS3D02G237800	1.5118	8.1546	1.21E-32	4.94E-30
TraesCS3B02G010200	-6.6348	1.3463	1.88E-32	7.60E-30
TraesCS6B02G252900	-1.5537	7.1796	2.10E-32	8.43E-30
TraesCS6D02G171100	2.2517	3.2398	2.15E-32	8.53E-30
TraesCS2B02G170400	-8.4463	1.2673	3.37E-32	1.33E-29
TraesCS5A02G007900	-1.9804	7.4551	3.40E-32	1.33E-29
TraesCS1A02G186600	2.9378	3.0239	3.73E-32	1.44E-29
TraesCS1A02G212300	-1.9881	4.1069	4.49E-32	1.72E-29
TraesCS7B02G103300	-2.3414	3.638	7.48E-32	2.84E-29
TraesCS5A02G257700	2.5128	2.8864	1.00E-31	3.76E-29
TraesCS1D02G149800	1.5395	7.8918	1.03E-31	3.83E-29
TraesCS2A02G176000	-1.6216	5.2066	1.21E-31	4.49E-29
TraesCS2A02G045800	-1.732	4.5948	1.67E-31	6.12E-29
TraesCS3A02G237400	1.4878	8.2042	2.01E-31	7.28E-29
TraesCS7D02G269400	2.3422	3.3149	2.09E-31	7.52E-29
TraesCS5D02G446900	2.2579	5.4912	2.43E-31	8.67E-29
TraesCS5D02G309900	-3.4676	1.9923	4.57E-31	1.62E-28
TraesCS3A02G113100	5.0271	1.6572	8.55E-31	3.00E-28
TraesCS2D02G183400	-1.5736	5.4706	1.18E-30	4.09E-28
TraesCS2A02G292400	-1.8229	5.4945	1.27E-30	4.38E-28
TraesCS2A02G191700	-2.0929	3.4043	2.19E-30	7.50E-28
TraesCS5D02G013400	-1.7938	7.8552	3.59E-30	1.22E-27
TraesCS5B02G241900	3.6509	1.8535	6.29E-30	2.12E-27
TraesCS2B02G167500	1.5494	5.8849	1.17E-29	3.90E-27
TraesCS6A02G249200	-2.1717	4.0585	1.21E-29	4.03E-27
TraesCS3D02G300600	3.7388	1.7413	1.24E-29	4.10E-27
TraesCS1D02G352600	1.5451	5.8486	1.41E-29	4.62E-27
TraesCS6D02G013400	-8.2972	1.1311	1.49E-29	4.84E-27
TraesCS2B02G236600	-2.1194	3.7816	2.32E-29	7.46E-27
TraesCSU02G050000	-8.4199	1.2402	2.49E-29	7.95E-27
TraesCS3A02G199200	-8.4209	1.2406	4.29E-29	1.36E-26
TraesCS2D02G280100	2.476	3.0405	4.94E-29	1.56E-26
TraesCS7D02G348500	3.2759	2.1058	6.18E-29	1.93E-26
TraesCS3D02G045700	3.0499	2.2268	7.23E-29	2.24E-26
TraesCS2B02G212400	1.9981	3.8293	1.70E-28	5.22E-26
TraesCS3B02G094700	-2.9006	2.6007	1.72E-28	5.22E-26
TraesCS2B02G202300	-1.5974	5.219	1.72E-28	5.22E-26
TraesCS3D02G375800	1.6654	6.7633	2.15E-28	6.49E-26
TraesCS4D02G104900	1.7183	5.3292	2.64E-28	7.90E-26

TraesCSU02G033600	-1.9172	3.9766	2.71E-28	8.07E-26
TraesCS4A02G061800	-2.8869	2.728	2.88E-28	8.49E-26
TraesCS1B02G053700	-4.1373	1.6618	3.31E-28	9.71E-26
TraesCS7B02G243000	-1.6347	5.895	4.80E-28	1.40E-25
TraesCS6B02G160400	-1.8292	7.1549	4.87E-28	1.41E-25
TraesCS1D02G044300	2.6782	3.2966	6.02E-28	1.73E-25
TraesCS1A02G228700	-8.4282	1.254	1.18E-27	3.38E-25
TraesCS2D02G271700	1.5604	5.249	1.51E-27	4.28E-25
TraesCS3B02G130300	4.9256	1.2896	1.64E-27	4.62E-25
TraesCS4A02G068200	4.6308	1.2991	1.67E-27	4.66E-25
TraesCS1D02G215300	-1.8891	4.0787	2.22E-27	6.18E-25
TraesCS6A02G138000	-1.9616	4.8798	3.51E-27	9.70E-25
TraesCS7D02G199400	-2.3142	3.1763	4.31E-27	1.18E-24
TraesCS4D02G187500	2.4684	2.7896	5.41E-27	1.47E-24
TraesCS2A02G161200	1.8453	3.6955	6.10E-27	1.65E-24
TraesCS3D02G185100	-1.8247	4.9818	1.21E-26	3.24E-24
TraesCS2D02G414900	2.0265	3.4735	1.99E-26	5.33E-24
TraesCS5D02G338400	4.2468	1.3821	2.59E-26	6.88E-24
TraesCS2A02G034000	2.6575	2.8326	2.66E-26	7.01E-24
TraesCS2B02G047400	3.4748	1.8712	3.08E-26	8.09E-24
TraesCS2B02G097400	3.694	1.6949	5.32E-26	1.39E-23
TraesCS2D02G193400	1.8016	4.1342	5.48E-26	1.42E-23
TraesCS1B02G300600	2.1918	4.821	6.23E-26	1.60E-23
TraesCS5D02G322600	-2.2249	2.8483	8.72E-26	2.23E-23
TraesCS3B02G272700	-1.5411	5.1119	1.46E-25	3.72E-23
TraesCS2D02G039700	-2.6928	2.5259	1.78E-25	4.51E-23
TraesCS2D02G033900	3.259	1.7582	2.44E-25	6.12E-23
TraesCS5B02G098300	-7.9854	0.8511	4.58E-25	1.14E-22
TraesCS5B02G234400	1.2859	6.9874	5.78E-25	1.44E-22
TraesCS1A02G349900	1.4059	6.0705	6.99E-25	1.73E-22
TraesCS6A02G023100	-1.5318	4.3778	7.81E-25	1.92E-22
TraesCS1D02G149700	1.3787	7.5471	1.29E-24	3.16E-22
TraesCS4B02G242600	-8.2129	1.0594	1.33E-24	3.23E-22
TraesCS2D02G168300	2.0164	3.2694	1.36E-24	3.28E-22
TraesCS4D02G338000	-2.656	2.4809	3.08E-24	7.39E-22
TraesCS4D02G201700	-1.8314	3.5983	3.12E-24	7.45E-22
TraesCS7B02G167100	2.2713	2.7514	4.07E-24	9.66E-22
TraesCS7A02G327300	2.3146	2.5907	5.63E-24	1.33E-21
TraesCS4D02G240400	2.0445	3.0903	6.07E-24	1.43E-21
TraesCS1A02G152800	1.8573	3.3642	6.57E-24	1.53E-21
TraesCS5D02G057000	-7.9689	0.8372	7.73E-24	1.80E-21
TraesCS7D02G071200	1.5515	4.1762	1.02E-23	2.35E-21
TraesCS6D02G295500	2.5372	2.5958	1.12E-23	2.56E-21
TraesCS2A02G081300	1.4651	4.8681	1.27E-23	2.91E-21
TraesCS1A02G043900	2.3425	3.3638	1.51E-23	3.42E-21
TraesCS3D02G407000	4.7076	1.0948	1.58E-23	3.58E-21
TraesCS3B02G220000	-8.0799	0.9326	1.93E-23	4.33E-21
TraesCS3D02G112100	-1.7751	3.9614	2.30E-23	5.15E-21

TraesCS7D02G339600	-1.5205	6.4539	2.50E-23	5.57E-21
TraesCS2B02G183600	-7.8811	0.7573	3.06E-23	6.77E-21
TraesCS2A02G245500	-6.1831	0.9425	4.72E-23	1.04E-20
TraesCS4B02G107800	1.43	5.3003	5.14E-23	1.13E-20
TraesCS4B02G237500	-3.1912	2.3637	5.49E-23	1.20E-20
TraesCS4D02G066400	1.5891	6.0025	5.57E-23	1.21E-20
TraesCS1A02G077700	-1.2291	6.5528	6.28E-23	1.36E-20
TraesCS1B02G096200	-1.9614	3.0743	6.95E-23	1.49E-20
TraesCS6D02G127100	-1.6642	4.9737	7.73E-23	1.65E-20
TraesCS5B02G257000	2.3362	2.7695	8.87E-23	1.88E-20
TraesCS5A02G234200	1.453	6.2924	1.00E-22	2.12E-20
TraesCS5A02G119800	7.7971	0.6569	1.15E-22	2.41E-20
TraesCS2A02G256200	1.896	3.1225	1.34E-22	2.80E-20
TraesCS6D02G135300	-1.8223	3.6523	1.65E-22	3.43E-20
TraesCS2D02G147900	-7.9847	0.8514	1.69E-22	3.51E-20
TraesCS6D02G392100	-1.7519	3.7436	2.14E-22	4.42E-20
TraesCS3D02G047800	-2.0604	3.2789	2.39E-22	4.91E-20
TraesCS4B02G066300	7.7444	0.6107	2.58E-22	5.27E-20
TraesCS2A02G062700	-1.9601	3.2666	2.83E-22	5.75E-20
TraesCS2B02G075800	-1.3838	7.6845	3.06E-22	6.19E-20
TraesCS3B02G327100	-1.4891	4.4775	3.61E-22	7.28E-20
TraesCS4A02G061700	3.9901	1.4738	3.91E-22	7.84E-20
TraesCS4D02G170300	-1.841	4.9393	4.38E-22	8.73E-20
TraesCS6A02G024100	-3.4647	1.4463	4.41E-22	8.76E-20
TraesCS7A02G176800	-1.4399	5.4321	4.73E-22	9.35E-20
TraesCS6D02G339100	-2.6404	2.519	4.89E-22	9.62E-20
TraesCS3B02G114400	-2.9673	1.8287	5.25E-22	1.03E-19
TraesCS7D02G178100	-1.468	5.6648	5.96E-22	1.16E-19
TraesCS2A02G065700	-1.4907	6.3986	6.47E-22	1.25E-19
TraesCS6A02G002700	-1.8407	3.1156	6.48E-22	1.25E-19
TraesCS2D02G391500	1.9377	3.4357	6.79E-22	1.31E-19
TraesCS1A02G181100	-1.6967	4.767	7.11E-22	1.36E-19
TraesCS2D02G061400	-1.4019	7.0355	7.69E-22	1.47E-19
TraesCS1B02G056900	2.2365	3.9849	7.86E-22	1.49E-19
TraesCS3A02G178600	-1.4296	4.4898	1.06E-21	2.00E-19
TraesCS4D02G102600	-1.3637	6.1753	1.27E-21	2.38E-19
TraesCS7B02G081800	-1.5217	5.5887	1.34E-21	2.51E-19
TraesCS6A02G048200	-1.7543	3.5607	1.35E-21	2.52E-19
TraesCS1B02G000100	-7.9391	0.8108	1.38E-21	2.55E-19
TraesCS6A02G158900	1.3156	4.8877	1.39E-21	2.56E-19
TraesCS2D02G061300	-1.5201	7.3293	1.42E-21	2.61E-19
TraesCS2D02G195900	-1.7029	3.3196	1.55E-21	2.85E-19
TraesCSU02G156200	-7.9412	0.8117	1.60E-21	2.92E-19
TraesCS4D02G343000	-1.4284	6.5728	1.85E-21	3.36E-19
TraesCS6D02G231200	-1.6952	3.4956	1.86E-21	3.36E-19
TraesCS6A02G023300	-1.4219	5.8916	2.57E-21	4.63E-19
TraesCS6B02G166200	-1.413	4.4169	2.92E-21	5.24E-19
TraesCS2B02G319000	-3.2387	1.9251	2.99E-21	5.34E-19

TraesCS3A02G184900	-1.6048	4.3202	3.62E-21	6.44E-19
TraesCS2B02G077700	-1.2516	7.7175	4.10E-21	7.25E-19
TraesCS5B02G169100	1.9589	3.312	4.32E-21	7.62E-19
TraesCS2D02G358200	-1.4678	4.9886	4.34E-21	7.63E-19
TraesCS5B02G140300	-7.7622	0.6531	4.40E-21	7.69E-19
TraesCS5A02G183300	1.2853	8.0085	4.56E-21	7.95E-19
TraesCS2A02G281200	2.371	2.1352	6.92E-21	1.20E-18
TraesCS7B02G256000	1.824	3.7855	7.43E-21	1.28E-18
TraesCS5B02G059800	2.8443	2.0947	7.90E-21	1.36E-18
TraesCS1A02G291200	1.9953	4.1716	1.11E-20	1.91E-18
TraesCS5D02G266000	2.7291	2.4623	1.19E-20	2.03E-18
TraesCS3D02G342100	-1.723	4.4479	1.25E-20	2.13E-18
TraesCS1B02G064800	-7.7389	0.6349	1.26E-20	2.13E-18
TraesCS5D02G418400	1.2418	5.5009	1.37E-20	2.30E-18
TraesCS2A02G215100	1.3233	5.4232	1.37E-20	2.30E-18
TraesCS6D02G153600	1.2834	4.7152	1.48E-20	2.47E-18
TraesCS2A02G061900	-1.4057	6.2839	1.83E-20	3.05E-18
TraesCS6A02G022100	-1.8458	3.0414	2.07E-20	3.44E-18
TraesCS4B02G067500	1.4563	6.6278	2.26E-20	3.73E-18
TraesCS2D02G415900	-2.0026	2.9995	2.28E-20	3.75E-18
TraesCS4D02G086200	7.6269	0.5093	2.56E-20	4.21E-18
TraesCS1A02G078100	-1.9907	2.6982	2.68E-20	4.37E-18
TraesCS5D02G464800	-1.3922	7.2059	2.68E-20	4.37E-18
TraesCS2A02G034100	2.7256	1.9985	2.89E-20	4.68E-18
TraesCS4A02G178800	1.2716	6.2298	3.23E-20	5.21E-18
TraesCS3D02G260300	1.1536	7.2189	3.42E-20	5.50E-18
TraesCS6D02G054400	-3.5877	1.5467	3.50E-20	5.62E-18
TraesCS4B02G237300	2.9869	1.7311	3.75E-20	5.99E-18
TraesCS4A02G027700	1.4506	4.1006	4.13E-20	6.57E-18
TraesCS2B02G215100	-1.7012	3.8247	4.19E-20	6.65E-18
TraesCS1D02G079800	-1.1847	6.3704	5.13E-20	8.10E-18
TraesCS2D02G082100	-2.3262	2.2063	5.72E-20	8.99E-18
TraesCS7A02G348600	-1.2577	6.9268	6.27E-20	9.82E-18
TraesCS4D02G042300	3.5922	1.2719	7.26E-20	1.13E-17
TraesCS1B02G169500	-1.3419	5.4591	7.36E-20	1.15E-17
TraesCS6B02G050700	-1.3165	4.8494	9.16E-20	1.42E-17
TraesCS6A02G094100	-2.5334	2.2928	9.58E-20	1.48E-17
TraesCS3B02G209900	-2.348	3.2453	9.65E-20	1.49E-17
TraesCS4B02G200600	-1.7466	3.7936	1.12E-19	1.72E-17
TraesCS5A02G164200	-1.4792	4.7691	1.13E-19	1.73E-17
TraesCS6D02G293900	1.5077	5.6559	1.22E-19	1.85E-17
TraesCSU02G134900	-6.2951	1.0322	1.36E-19	2.06E-17
TraesCS7B02G165900	3.1774	1.449	1.47E-19	2.23E-17
TraesCS3A02G292500	-1.463	4.5544	1.57E-19	2.36E-17
TraesCS4A02G201700	-1.2311	6.0426	1.80E-19	2.70E-17
TraesCS2B02G334600	-1.8589	3.0159	1.88E-19	2.82E-17
TraesCS4A02G103400	-1.722	3.7327	2.01E-19	3.00E-17
TraesCS1D02G080300	-2.079	3.0201	2.19E-19	3.26E-17

TraesCS2D02G081200	3.8433	1.0328	2.42E-19	3.58E-17
TraesCS1B02G225900	-1.7348	3.6162	2.67E-19	3.94E-17
TraesCS6B02G260100	1.6232	3.4078	2.75E-19	4.05E-17
TraesCS3D02G341600	-1.653	4.5956	3.05E-19	4.46E-17
TraesCS6A02G093700	-1.2275	5.7197	3.10E-19	4.52E-17
TraesCS5D02G392000	-1.1657	5.5674	3.48E-19	5.06E-17
TraesCS6D02G250000	-1.4989	4.297	3.70E-19	5.37E-17
TraesCS4D02G206600	1.7996	3.344	3.73E-19	5.39E-17
TraesCS1B02G199500	-1.4314	4.4647	4.66E-19	6.71E-17
TraesCS3B02G019900	-1.8674	2.8845	4.73E-19	6.79E-17
TraesCS2D02G116600	-2.1873	3.2228	5.02E-19	7.18E-17
TraesCS3A02G137200	2.6802	2.579	5.76E-19	8.21E-17
TraesCS4D02G263500	1.2706	5.2706	6.08E-19	8.64E-17
TraesCS1B02G177900	-1.6146	3.3503	6.24E-19	8.84E-17
TraesCS6A02G110400	-1.5661	4.4718	6.76E-19	9.54E-17
TraesCS2D02G044400	-2.0903	2.8431	6.90E-19	9.71E-17
TraesCS4B02G105800	-1.2864	4.9234	6.92E-19	9.71E-17
TraesCS2B02G075700	-1.3919	6.2544	7.44E-19	1.04E-16
TraesCS6D02G099100	-1.5521	4.4274	8.52E-19	1.19E-16
TraesCS6B02G095100	1.9263	3.167	8.58E-19	1.19E-16
TraesCS4D02G337500	-1.7732	3.9663	9.29E-19	1.29E-16
TraesCS2A02G270100	-3.3499	1.1205	9.77E-19	1.35E-16
TraesCS6A02G097400	2.6869	1.6627	9.78E-19	1.35E-16
TraesCS7A02G266300	-1.8419	2.9694	1.00E-18	1.37E-16
TraesCS6D02G088800	-1.2184	5.8191	1.03E-18	1.41E-16
TraesCS6D02G206200	-1.2369	8.5141	1.10E-18	1.50E-16
TraesCS4D02G254100	1.0808	6.5196	1.23E-18	1.67E-16
TraesCS1B02G279100	-1.5638	6.8134	1.25E-18	1.69E-16
TraesCS1A02G161400	-1.6652	3.5376	1.39E-18	1.87E-16
TraesCS5D02G427000	-1.7588	3.6845	1.45E-18	1.95E-16
TraesCS6A02G242500	-1.8352	3.8067	1.47E-18	1.98E-16
TraesCS5D02G427200	-1.7392	3.1423	1.50E-18	2.01E-16
TraesCS5D02G169100	-1.3314	4.1327	1.82E-18	2.42E-16
TraesCS2A02G191600	-1.4667	3.5656	1.86E-18	2.46E-16
TraesCS7A02G331300	-1.1839	6.4013	1.91E-18	2.53E-16
TraesCS4D02G032600	-2.0129	3.2605	2.08E-18	2.75E-16
TraesCS5A02G312500	-1.1984	5.4412	2.17E-18	2.85E-16
TraesCS2A02G063000	-1.4988	4.6339	2.27E-18	2.98E-16
TraesCS5B02G263700	-1.8866	2.6411	2.43E-18	3.18E-16
TraesCS1B02G203100	2.9157	1.3907	2.63E-18	3.42E-16
TraesCS2D02G018000	-1.2725	4.7948	2.68E-18	3.48E-16
TraesCS7D02G022600	-1.2681	4.6122	2.72E-18	3.53E-16
TraesCS5A02G008700	-7.4697	0.4012	2.85E-18	3.68E-16
TraesCS6B02G031600	-1.1936	6.527	3.01E-18	3.88E-16
TraesCS3B02G289200	-7.4476	0.3825	3.35E-18	4.29E-16
TraesCS4B02G240700	2.7419	2.6404	3.47E-18	4.44E-16
TraesCS6B02G291900	-1.3417	4.7621	3.64E-18	4.65E-16
TraesCS5A02G152100	-1.3262	4.3696	3.70E-18	4.70E-16

TraesCS2D02G041600	-2.156	2.2906	3.74E-18	4.75E-16
TraesCS6A02G213900	-1.3033	4.9977	3.84E-18	4.85E-16
TraesCS5B02G048000	-1.3121	5.9671	3.94E-18	4.96E-16
TraesCS5B02G071800	3.444	1.6609	4.06E-18	5.10E-16
TraesCS4D02G238800	-1.3526	6.2544	4.27E-18	5.34E-16
TraesCSU02G125200	2.3721	3.582	4.27E-18	5.34E-16
TraesCS2B02G308900	-1.477	5.2305	4.40E-18	5.48E-16
TraesCS6D02G090200	-1.0684	6.0969	4.42E-18	5.49E-16
TraesCS4B02G050800	3.8746	0.8704	5.84E-18	7.23E-16
TraesCS1B02G203000	2.9047	1.3814	5.88E-18	7.26E-16
TraesCS6D02G231300	-1.676	3.9659	6.05E-18	7.45E-16
TraesCS7D02G089300	-1.2211	6.4729	6.68E-18	8.20E-16
TraesCS1A02G314900	2.5887	2.78	6.96E-18	8.53E-16
TraesCS4D02G234000	-1.9519	2.8031	7.46E-18	9.11E-16
TraesCS5B02G058800	-1.2585	4.6442	8.32E-18	1.01E-15
TraesCS4A02G042700	1.4531	4.3352	9.14E-18	1.11E-15
TraesCS4A02G068900	-1.37	4.1911	9.80E-18	1.19E-15
TraesCS3D02G369700	-7.4889	0.4187	1.04E-17	1.26E-15
TraesCS7B02G113200	-1.9354	2.579	1.09E-17	1.31E-15
TraesCS5A02G201200	2.2466	2.3618	1.19E-17	1.43E-15
TraesCS6B02G282000	-1.8605	3.9727	1.41E-17	1.68E-15
TraesCS4A02G063600	1.1885	5.5385	1.42E-17	1.69E-15
TraesCS1D02G148900	-1.2437	5.2739	1.53E-17	1.82E-15
TraesCS7A02G097100	-1.572	5.0273	1.66E-17	1.98E-15
TraesCS6A02G047500	-1.7358	4.3175	1.83E-17	2.17E-15
TraesCS7D02G212900	-1.5321	4.3411	2.08E-17	2.46E-15
TraesCS2B02G215000	-1.5956	3.3442	2.09E-17	2.46E-15
TraesCS1B02G308900	1.4648	4.0627	2.31E-17	2.72E-15
TraesCS4D02G357200	-1.8015	2.5712	2.39E-17	2.80E-15
TraesCS2B02G313900	-1.5126	5.7429	2.71E-17	3.16E-15
TraesCS2D02G376800	-1.244	4.7174	2.96E-17	3.45E-15
TraesCS1A02G317900	-1.7748	4.9931	3.11E-17	3.61E-15
TraesCS5B02G023500	7.5752	0.4614	3.37E-17	3.90E-15
TraesCS6A02G012700	-4.0061	0.814	3.44E-17	3.97E-15
TraesCS1A02G028700	-4.5931	0.7154	3.48E-17	4.01E-15
TraesCS5D02G364100	-2.06	2.3603	3.91E-17	4.50E-15
TraesCS4A02G140700	-7.4108	0.3487	4.16E-17	4.76E-15
TraesCS4B02G225300	3.4228	1.1301	4.42E-17	5.05E-15
TraesCS1D02G092400	2.7823	1.4985	4.50E-17	5.13E-15
TraesCS2A02G063100	-1.26	6.5548	4.86E-17	5.52E-15
TraesCS3B02G034100	-1.4258	3.5868	5.40E-17	6.12E-15
TraesCS7A02G087700	-1.8464	2.4891	6.20E-17	7.00E-15
TraesCS1D02G317700	-1.7268	5.0637	6.58E-17	7.42E-15
TraesCS4A02G027800	1.317	7.2788	7.22E-17	8.12E-15
TraesCS4D02G286400	-1.3975	4.5461	7.31E-17	8.19E-15
TraesCS1A02G152100	-1.2714	4.6923	7.60E-17	8.50E-15
TraesCS6A02G228800	-1.099	6.6652	9.30E-17	1.04E-14
TraesCS1B02G136800	1.5751	4.6064	9.81E-17	1.09E-14

TraesCS5D02G360600	2.2541	2.756	1.01E-16	1.12E-14
TraesCSU02G010400	-7.2645	0.2279	1.14E-16	1.26E-14
TraesCS2A02G053200	1.4027	3.9153	1.18E-16	1.30E-14
TraesCS3D02G283300	-1.1757	5.8512	1.21E-16	1.33E-14
TraesCS3A02G235900	-1.4777	3.8153	1.27E-16	1.39E-14
TraesCS5D02G069200	-1.5109	4.5486	1.27E-16	1.39E-14
TraesCS2D02G079100	1.1159	5.5588	1.30E-16	1.42E-14
TraesCS3D02G035900	-2.7672	1.3274	1.37E-16	1.49E-14
TraesCS6A02G035200	1.6738	3.8437	1.38E-16	1.50E-14
TraesCS4B02G018100	-1.8832	3.1531	1.44E-16	1.57E-14
TraesCS5B02G338100	-7.8473	0.7375	1.47E-16	1.59E-14
TraesCS2D02G186000	-2.0963	2.4457	1.83E-16	1.97E-14
TraesCS2D02G063900	-1.1077	8.1117	1.89E-16	2.03E-14
TraesCSU02G057500	-1.3122	4.1988	2.05E-16	2.20E-14
TraesCS3A02G048000	-1.3347	3.8181	2.06E-16	2.21E-14
TraesCS2D02G210700	-1.5033	4.3711	2.09E-16	2.24E-14
TraesCS4B02G097200	1.1069	5.8966	2.12E-16	2.26E-14
TraesCS1D02G404700	-1.1377	6.5335	2.35E-16	2.50E-14
TraesCS5B02G091200	-1.352	3.7576	2.43E-16	2.57E-14
TraesCS7D02G032500	-1.0943	5.9168	2.46E-16	2.60E-14
TraesCS2D02G033500	3.1758	1.0465	2.49E-16	2.62E-14
TraesCS7D02G417400	-1.5687	4.7278	2.49E-16	2.62E-14
TraesCS1B02G134600	-1.5516	3.7998	2.74E-16	2.88E-14
TraesCS3D02G323400	1.3445	5.8544	2.78E-16	2.91E-14
TraesCS6A02G132500	-2.7847	1.7795	2.84E-16	2.97E-14
TraesCS6D02G224500	-1.7242	3.6859	2.95E-16	3.08E-14
TraesCS7B02G003000	-1.0258	7.023	3.18E-16	3.30E-14
TraesCS1B02G183500	1.2779	4.6544	3.86E-16	4.00E-14
TraesCS3D02G182100	-1.1904	4.2353	3.97E-16	4.10E-14
TraesCS4B02G034100	-1.8737	3.1491	3.99E-16	4.12E-14
TraesCS4D02G358700	-1.3125	4.6365	4.09E-16	4.21E-14
TraesCS7A02G160300	1.6661	6.6345	4.22E-16	4.33E-14
TraesCS1D02G118000	1.4814	3.854	4.83E-16	4.94E-14
TraesCS4B02G019800	-2.5324	1.5293	4.94E-16	5.04E-14
TraesCS2D02G061100	-1.5327	4.8933	5.08E-16	5.18E-14
TraesCS2B02G232800	-1.2601	4.0891	5.47E-16	5.56E-14
TraesCS7D02G190200	-1.7123	3.0139	5.80E-16	5.88E-14
TraesCS2A02G198000	-1.1772	5.6294	5.94E-16	6.01E-14
TraesCS7D02G079000	-1.6222	2.9052	6.00E-16	6.06E-14
TraesCS2D02G042300	4.4749	0.5621	6.10E-16	6.14E-14
TraesCS1D02G030500	1.0483	7.4322	6.21E-16	6.24E-14
TraesCS5A02G219700	1.4884	4.3734	6.38E-16	6.38E-14
TraesCS1D02G382000	1.2754	6.203	6.61E-16	6.59E-14
TraesCS3A02G180200	-2.0724	2.8316	6.66E-16	6.63E-14
TraesCS4B02G232800	-1.6558	2.9302	6.71E-16	6.66E-14
TraesCS3B02G259200	1.7617	2.7505	7.33E-16	7.26E-14
TraesCS4D02G317300	1.7116	3.9316	7.62E-16	7.53E-14
TraesCS1B02G213600	-1.1801	6.286	7.64E-16	7.53E-14

TraesCS3B02G224500	-1.9081	2.7164	8.00E-16	7.87E-14
TraesCS3B02G210000	-2.5396	2.459	8.17E-16	8.01E-14
TraesCS2D02G228500	1.4524	5.1989	8.80E-16	8.59E-14
TraesCS6B02G031700	-1.2055	6.0835	9.15E-16	8.92E-14
TraesCS2A02G285900	2.2052	1.7505	1.07E-15	1.04E-13
TraesCS5A02G219000	2.2957	1.6511	1.11E-15	1.07E-13
TraesCS1D02G296700	2.408	2.4369	1.26E-15	1.22E-13
TraesCSU02G146800	-1.231	4.6762	1.34E-15	1.29E-13
TraesCS5B02G150700	-1.3109	4.2744	1.41E-15	1.36E-13
TraesCS6D02G054800	-1.5928	4.597	1.48E-15	1.42E-13
TraesCS5B02G245700	4.3736	0.4766	1.49E-15	1.43E-13
TraesCS6A02G296200	1.2901	4.1075	1.60E-15	1.53E-13
TraesCS6A02G000300	-1.5152	4.1694	1.64E-15	1.57E-13
TraesCS5D02G449400	-1.5099	3.3644	1.75E-15	1.67E-13
TraesCS7B02G113000	-1.088	4.9223	1.79E-15	1.70E-13
TraesCS1B02G122200	4.0979	0.5764	1.83E-15	1.74E-13
TraesCS7B02G171300	-1.6766	3.1417	1.96E-15	1.85E-13
TraesCS5B02G199200	1.4895	3.8333	2.03E-15	1.92E-13
TraesCS2D02G418300	4.0198	1.1821	2.21E-15	2.08E-13
TraesCS2A02G194500	1.32	4.4162	2.21E-15	2.08E-13
TraesCSU02G146600	1.562	4.7439	2.23E-15	2.09E-13
TraesCS2B02G205000	-2.0962	2.2288	2.31E-15	2.16E-13
TraesCS4D02G188800	-1.6774	2.5664	2.33E-15	2.18E-13
TraesCS2A02G210700	-2.969	1.141	2.34E-15	2.18E-13
TraesCS7D02G093200	-1.4482	6.0278	2.34E-15	2.18E-13
TraesCS2D02G015000	-1.5278	3.0094	2.46E-15	2.28E-13
TraesCS4A02G016600	-1.2832	4.0223	2.70E-15	2.49E-13
TraesCS1D02G239000	1.5418	3.9261	2.78E-15	2.56E-13
TraesCS1D02G433600	1.4021	3.2164	2.89E-15	2.65E-13
TraesCS5D02G053200	-1.1967	6.6614	2.93E-15	2.69E-13
TraesCS2A02G153000	-1.0578	5.0201	2.98E-15	2.73E-13
TraesCS7A02G075600	1.5906	4.3002	3.02E-15	2.76E-13
TraesCS1B02G257700	1.1448	4.2662	3.05E-15	2.78E-13
TraesCS2B02G095900	1.1511	6.1784	3.09E-15	2.81E-13
TraesCS3A02G118300	1.113	5.036	3.36E-15	3.05E-13
TraesCS3D02G418900	-7.1871	0.1641	3.51E-15	3.18E-13
TraesCS5A02G281700	2.2032	2.896	3.59E-15	3.24E-13
TraesCS2A02G062100	-1.2611	6.3225	3.59E-15	3.24E-13
TraesCS7A02G083800	-1.5316	2.9132	3.78E-15	3.40E-13
TraesCS2B02G370200	-1.2939	5.6887	4.06E-15	3.64E-13
TraesCS3D02G326900	-1.1788	7.8062	4.21E-15	3.77E-13
TraesCS6D02G041700	-1.1025	4.9266	4.31E-15	3.86E-13
TraesCS5A02G089400	-1.4608	3.1362	4.34E-15	3.87E-13
TraesCS1B02G100600	-1.3753	4.2256	4.58E-15	4.08E-13
TraesCS2D02G033700	1.4806	3.5103	6.13E-15	5.45E-13
TraesCS3D02G067300	1.6112	4.0911	6.78E-15	6.01E-13
TraesCS7D02G347300	-1.3105	6.3098	6.79E-15	6.01E-13
TraesCS2B02G014700	-2.4858	1.5518	7.18E-15	6.34E-13

TraesCS3D02G105500	-1.1141	5.6497	7.44E-15	6.56E-13
TraesCS3B02G021700	-1.568	2.959	7.80E-15	6.86E-13
TraesCS2D02G016800	-1.1352	4.6493	7.88E-15	6.91E-13
TraesCS3D02G055000	-5.4258	0.2878	8.00E-15	7.01E-13
TraesCS2A02G034500	2.0362	2.0327	8.20E-15	7.17E-13
TraesCS5D02G388200	-1.5081	3.7394	8.43E-15	7.35E-13
TraesCS4B02G207000	-1.6455	2.7914	8.45E-15	7.36E-13
TraesCSU02G243700	-1.4213	5.9382	8.72E-15	7.58E-13
TraesCS5D02G441000	-1.7597	2.6969	8.84E-15	7.67E-13
TraesCS2B02G220800	-1.9959	2.0465	9.10E-15	7.87E-13
TraesCS4A02G092100	1.6344	2.7784	9.34E-15	8.07E-13
TraesCS7D02G036600	-1.251	7.0541	9.64E-15	8.31E-13
TraesCS7A02G024600	-1.4375	3.1125	1.01E-14	8.70E-13
TraesCS4D02G361500	-2.0412	1.9212	1.09E-14	9.39E-13
TraesCS3D02G187300	-1.1786	4.5026	1.12E-14	9.63E-13
TraesCS1D02G393600	-1.0732	5.7237	1.13E-14	9.63E-13
TraesCS1B02G293100	-1.3607	5.2299	1.14E-14	9.70E-13
TraesCS4D02G016300	-1.723	2.5831	1.23E-14	1.05E-12
TraesCS3D02G345100	-2.2255	1.9899	1.23E-14	1.05E-12
TraesCS7D02G171000	5.456	0.2679	1.29E-14	1.09E-12
TraesCS3B02G137400	1.0341	5.7985	1.32E-14	1.12E-12
TraesCS1D02G084400	-1.2061	3.9727	1.33E-14	1.12E-12
TraesCS7A02G360400	7.1917	0.1437	1.33E-14	1.12E-12
TraesCS2D02G158200	-1.1229	4.7223	1.36E-14	1.14E-12
TraesCS1D02G309200	-1.0589	6.1144	1.40E-14	1.18E-12
TraesCS5B02G270300	1.9382	2.0962	1.43E-14	1.19E-12
TraesCS2D02G021600	2.3209	1.5818	1.45E-14	1.21E-12
TraesCS1A02G069900	1.6048	4.9216	1.49E-14	1.24E-12
TraesCS5A02G152400	1.192	4.6158	1.51E-14	1.26E-12
TraesCS2B02G221800	-1.1222	6.9313	1.53E-14	1.27E-12
TraesCS7D02G153100	-7.5047	0.4234	1.64E-14	1.36E-12
TraesCS2B02G138500	-1.927	2.86	1.64E-14	1.36E-12
TraesCS7D02G168600	2.0113	2.2026	1.65E-14	1.36E-12
TraesCS2B02G075400	-1.218	5.289	1.70E-14	1.40E-12
TraesCS5B02G007100	-1.1567	4.4634	1.77E-14	1.46E-12
TraesCS7B02G271900	-1.1176	4.5468	1.78E-14	1.46E-12
TraesCS7A02G113600	-1.1805	6.7158	1.83E-14	1.50E-12
TraesCS1A02G283900	-1.2736	4.897	1.85E-14	1.52E-12
TraesCS1D02G270000	-1.2612	3.991	1.86E-14	1.52E-12
TraesCS2B02G098400	-2.7342	1.2935	1.88E-14	1.53E-12
TraesCS4A02G061900	-1.2878	5.474	1.90E-14	1.55E-12
TraesCS2B02G018600	-3.2382	1.2615	2.00E-14	1.62E-12
TraesCS2A02G259900	-1.2488	4.4352	2.08E-14	1.69E-12
TraesCS2D02G026000	-2.341	2.0911	2.12E-14	1.72E-12
TraesCS1B02G119800	1.7174	2.6556	2.25E-14	1.82E-12
TraesCS2D02G311400	7.0583	0.032	2.28E-14	1.83E-12
TraesCS2A02G062000	-1.2161	6.1783	2.30E-14	1.85E-12
TraesCS2B02G232700	-1.3631	4.1388	2.42E-14	1.95E-12

TraesCS4A02G226700	3.2174	1.0695	2.68E-14	2.15E-12
TraesCS6B02G092000	1.3255	4.051	2.75E-14	2.20E-12
TraesCS1A02G083100	-1.2718	3.9804	2.89E-14	2.31E-12
TraesCS4D02G050600	-1.3066	5.9921	2.98E-14	2.38E-12
TraesCS2D02G404600	-1.1418	5.1023	2.99E-14	2.38E-12
TraesCS3B02G094100	-7.1685	0.1454	3.05E-14	2.43E-12
TraesCS3B02G206100	-1.0418	4.8956	3.09E-14	2.45E-12
TraesCS1A02G270000	-1.2512	4.1847	3.15E-14	2.49E-12
TraesCS6D02G223100	1.1843	4.091	3.17E-14	2.50E-12
TraesCS5D02G400700	1.1982	5.7454	3.43E-14	2.70E-12
TraesCS5D02G152400	-1.2928	5.2939	3.45E-14	2.71E-12
TraesCS2A02G034200	2.5697	1.7341	3.61E-14	2.84E-12
TraesCS2D02G290300	-1.2158	5.4004	3.70E-14	2.90E-12
TraesCS2A02G205300	-1.5033	3.5563	3.81E-14	2.98E-12
TraesCS5B02G304900	1.4978	5.1425	3.86E-14	3.01E-12
TraesCS4B02G207100	-2.5308	1.2775	3.95E-14	3.08E-12
TraesCS3B02G123700	1.7645	2.9516	4.71E-14	3.66E-12
TraesCS2D02G108500	-1.4656	4.1483	4.72E-14	3.66E-12
TraesCS2D02G186200	-1.9093	2.5701	4.78E-14	3.70E-12
TraesCS6B02G192400	1.081	5.3596	4.81E-14	3.72E-12
TraesCS5D02G248700	-1.0628	5.1168	4.87E-14	3.75E-12
TraesCS7D02G095900	-1.0305	5.9933	5.05E-14	3.89E-12
TraesCS2A02G062800	-1.2911	5.6272	5.21E-14	4.00E-12
TraesCS5D02G014100	-3.508	0.6204	5.49E-14	4.21E-12
TraesCS3B02G146500	1.483	3.6437	5.97E-14	4.57E-12
TraesCS2B02G177900	-1.0482	4.6954	6.34E-14	4.85E-12
TraesCS6B02G063800	-1.1877	6.3062	6.65E-14	5.07E-12
TraesCS4B02G212300	1.9864	2.2673	6.90E-14	5.25E-12
TraesCS1D02G026500	-1.3746	5.5979	7.01E-14	5.33E-12
TraesCS7A02G233300	2.2633	1.8795	8.26E-14	6.27E-12
TraesCS4D02G342600	-1.2695	3.9856	8.38E-14	6.34E-12
TraesCSU02G035900	-1.1503	4.226	8.58E-14	6.49E-12
TraesCS2A02G016500	-1.1612	4.8909	8.67E-14	6.54E-12
TraesCS1D02G260700	1.755	3.1298	8.88E-14	6.69E-12
TraesCS5B02G092500	-1.0776	5.8993	8.96E-14	6.74E-12
TraesCS4D02G130700	-1.55	2.7609	9.24E-14	6.94E-12
TraesCS2D02G061200	-1.7092	3.9808	9.38E-14	7.03E-12
TraesCS5A02G219400	2.0396	1.7328	9.64E-14	7.21E-12
TraesCS2D02G327800	-6.9943	0.0064	9.70E-14	7.24E-12
TraesCS7A02G188900	-1.89	2.9258	1.02E-13	7.62E-12
TraesCS4D02G200700	1.6118	2.5617	1.05E-13	7.77E-12
TraesCS7B02G115300	-1.1913	4.3508	1.17E-13	8.67E-12
TraesCS5D02G111200	-7.0801	0.076	1.21E-13	8.95E-12
TraesCS1D02G218800	-2.0376	2.5193	1.22E-13	8.99E-12
TraesCS5B02G161700	-1.3614	4.1874	1.22E-13	9.01E-12
TraesCS1D02G433300	-1.8753	2.5999	1.26E-13	9.29E-12
TraesCS3A02G283400	-1.1032	5.9022	1.31E-13	9.62E-12
TraesCS6A02G264900	-1.3302	4.7142	1.36E-13	9.98E-12

TraesCS2D02G173300	1.1666	6.1276	1.39E-13	1.02E-11
TraesCS6A02G040400	-1.669	2.8135	1.42E-13	1.04E-11
TraesCS2D02G081900	-1.2099	3.7107	1.47E-13	1.08E-11
TraesCS1B02G183100	1.5093	2.9118	1.58E-13	1.15E-11
TraesCS4A02G171900	-1.3574	3.2608	1.64E-13	1.19E-11
TraesCS1A02G248900	1.2292	4.1392	1.69E-13	1.23E-11
TraesCS5A02G153400	2.9255	0.8432	1.70E-13	1.24E-11
TraesCS5D02G243100	1.0079	7.0446	1.72E-13	1.25E-11
TraesCS5D02G468600	-1.3296	4.6518	1.73E-13	1.25E-11
TraesCS4A02G134100	1.445	6.5303	1.75E-13	1.26E-11
TraesCS7B02G218400	7.0238	0.006	1.79E-13	1.29E-11
TraesCS1B02G310100	1.0602	5.0158	1.80E-13	1.29E-11
TraesCS6D02G285300	-1.5837	2.8037	1.81E-13	1.30E-11
TraesCS3D02G185000	-1.7711	4.0016	1.84E-13	1.32E-11
TraesCS1D02G283000	-1.2311	5.426	1.87E-13	1.34E-11
TraesCS5B02G261700	-1.8905	3.039	1.88E-13	1.34E-11
TraesCS7A02G213300	-1.1683	3.7687	1.91E-13	1.36E-11
TraesCS4B02G032700	-7.0286	0.0318	1.99E-13	1.41E-11
TraesCS2D02G202200	-1.0952	7.1865	2.11E-13	1.49E-11
TraesCS4D02G170600	-1.916	2.5889	2.11E-13	1.49E-11
TraesCS5D02G402600	3.5332	0.7836	2.27E-13	1.60E-11
TraesCS6B02G196500	1.1824	4.254	2.27E-13	1.60E-11
TraesCS2B02G024500	-1.0725	5.0699	2.28E-13	1.60E-11
TraesCS2D02G332500	-1.1202	4.2749	2.31E-13	1.62E-11
TraesCS6A02G181000	2.7137	1.3846	2.44E-13	1.71E-11
TraesCS2D02G004800	-1.9907	2.0239	2.47E-13	1.73E-11
TraesCS5B02G152000	-1.1405	4.2947	2.67E-13	1.87E-11
TraesCS5B02G219300	-2.3052	2.2805	2.72E-13	1.89E-11
TraesCS2D02G322600	-2.4846	1.2471	2.91E-13	2.03E-11
TraesCS2A02G222500	1.1213	5.0998	3.09E-13	2.14E-11
TraesCS4D02G004000	-1.2671	3.5148	3.14E-13	2.17E-11
TraesCS5D02G127300	1.3482	3.2552	3.14E-13	2.17E-11
TraesCS5B02G160700	-1.3065	3.5863	3.19E-13	2.20E-11
TraesCS1A02G339400	-1.2963	4.7645	3.20E-13	2.20E-11
TraesCS2A02G025500	2.3072	1.7129	3.27E-13	2.25E-11
TraesCS2A02G297700	-1.162	5.7165	3.36E-13	2.31E-11
TraesCS7D02G367100	-1.0314	5.0434	3.46E-13	2.38E-11
TraesCS3D02G115000	5.3294	0.1644	3.55E-13	2.43E-11
TraesCS6A02G243900	-1.1946	3.9509	3.64E-13	2.49E-11
TraesCS5B02G246200	-1.263	4.2422	3.69E-13	2.52E-11
TraesCS5A02G151400	-1.7703	2.9274	3.69E-13	2.52E-11
TraesCS4A02G089900	-1.1132	4.2609	3.75E-13	2.55E-11
TraesCS3B02G049800	7.009	-0.0122	3.76E-13	2.55E-11
TraesCS6A02G222900	-1.1711	8.037	3.80E-13	2.58E-11
TraesCS5D02G238800	-1.3595	4.5109	3.82E-13	2.59E-11
TraesCS4B02G014700	-1.0554	6.9898	3.83E-13	2.59E-11
TraesCS3D02G185200	-1.8944	4.1928	3.85E-13	2.60E-11
TraesCS4D02G337400	-1.3474	3.7823	3.86E-13	2.60E-11

TraesCS3B02G131300	3.0965	0.7318	3.88E-13	2.61E-11
TraesCS1D02G341200	-1.0488	5.4125	3.97E-13	2.67E-11
TraesCS6D02G002000	-6.9913	0.0049	4.14E-13	2.78E-11
TraesCS2D02G350200	-1.1931	5.2645	4.17E-13	2.80E-11
TraesCS5B02G280900	3.1321	0.761	4.25E-13	2.85E-11
TraesCS7A02G009100	-1.2772	3.565	4.26E-13	2.85E-11
TraesCS1A02G259700	1.1771	6.3816	4.37E-13	2.91E-11
TraesCS5A02G004500	1.4729	3.4984	4.41E-13	2.94E-11
TraesCS2D02G265100	-1.6505	3.2196	4.64E-13	3.09E-11
TraesCS4D02G279400	-1.0019	4.8691	4.97E-13	3.30E-11
TraesCS7D02G149000	-1.89	2.6396	4.98E-13	3.30E-11
TraesCS4B02G029600	-1.0038	6.6987	5.03E-13	3.33E-11
TraesCS7B02G093700	-1.9933	2.9134	5.25E-13	3.47E-11
TraesCS3B02G310100	1.8067	2.4802	5.29E-13	3.49E-11
TraesCS5B02G089500	-1.2932	3.6489	5.41E-13	3.56E-11
TraesCS5D02G485800	-1.0841	6.6072	5.60E-13	3.68E-11
TraesCS2D02G029500	2.1706	1.5103	5.62E-13	3.69E-11
TraesCS5D02G278200	2.9453	0.8586	5.62E-13	3.69E-11
TraesCS5A02G017200	-1.7358	2.2098	5.68E-13	3.72E-11
TraesCS5D02G400800	1.1456	7.3794	5.72E-13	3.74E-11
TraesCS5B02G207900	1.0047	6.3081	6.02E-13	3.93E-11
TraesCS7D02G008700	-1.227	4.1507	6.05E-13	3.93E-11
TraesCS4B02G005800	-3.9575	0.5113	6.32E-13	4.11E-11
TraesCS1D02G036000	1.475	3.2092	6.47E-13	4.20E-11
TraesCSU02G024800	-1.253	5.8153	6.54E-13	4.24E-11
TraesCS7D02G386000	-1.1676	4.6493	6.57E-13	4.25E-11
TraesCS3A02G180300	-1.9243	3.3106	6.62E-13	4.28E-11
TraesCS4B02G007100	-1.0708	4.4059	6.67E-13	4.30E-11
TraesCS6D02G226100	-1.0394	4.4363	6.71E-13	4.32E-11
TraesCS5A02G114700	6.9069	-0.0898	6.81E-13	4.38E-11
TraesCS4A02G116900	-1.4859	2.9259	7.10E-13	4.55E-11
TraesCS5B02G007000	-1.1206	4.3304	7.16E-13	4.59E-11
TraesCS5D02G428000	-2.0378	2.3381	7.44E-13	4.76E-11
TraesCS1A02G107500	-1.2376	5.4872	7.62E-13	4.86E-11
TraesCS7D02G189400	3.7372	0.533	7.75E-13	4.94E-11
TraesCS5D02G488400	1.7617	3.0979	7.86E-13	5.00E-11
TraesCS5D02G413400	-1.168	3.6845	7.92E-13	5.03E-11
TraesCS6A02G150200	-2.1548	2.3662	8.09E-13	5.13E-11
TraesCS6D02G173000	-6.9678	-0.0168	8.13E-13	5.15E-11
TraesCS4D02G051900	1.0252	4.5297	8.14E-13	5.15E-11
TraesCS6D02G121100	1.8766	2.4702	8.24E-13	5.20E-11
TraesCS2A02G175000	-1.6282	3.2793	8.58E-13	5.41E-11
TraesCS5A02G229000	1.1757	5.7164	8.61E-13	5.42E-11
TraesCS2D02G030600	1.2018	3.7186	8.73E-13	5.49E-11
TraesCS7B02G290600	-1.1958	3.5866	9.18E-13	5.76E-11
TraesCS5A02G225600	-1.5352	3.0398	9.22E-13	5.77E-11
TraesCS7D02G332100	3.896	0.4085	9.28E-13	5.81E-11
TraesCS5A02G016200	1.1038	4.0297	9.67E-13	6.04E-11

TraesCS5A02G097800	-1.121	4.2224	9.74E-13	6.08E-11
TraesCS6D02G060900	1.642	3.1501	1.02E-12	6.36E-11
TraesCS3B02G130900	4.6169	0.2484	1.04E-12	6.44E-11
TraesCS2D02G375500	-1.7927	2.4183	1.06E-12	6.59E-11
TraesCS3B02G104300	-1.1414	4.1606	1.08E-12	6.67E-11
TraesCS6A02G063400	-2.4401	1.5065	1.13E-12	6.97E-11
TraesCS2B02G038600	1.8768	2.1105	1.16E-12	7.14E-11
TraesCS3D02G064000	-6.9317	-0.0434	1.16E-12	7.16E-11
TraesCS4B02G237600	-1.1844	4.9347	1.19E-12	7.34E-11
TraesCSU02G095400	-1.1653	6.0587	1.21E-12	7.42E-11
TraesCS6D02G227200	-1.1319	4.3891	1.29E-12	7.93E-11
TraesCS5B02G181500	1.0343	7.8159	1.34E-12	8.21E-11
TraesCS1A02G217000	-2.5849	0.9318	1.37E-12	8.38E-11
TraesCS2A02G034400	2.0573	1.5945	1.41E-12	8.60E-11
TraesCS1D02G081700	-1.554	2.736	1.41E-12	8.63E-11
TraesCS5D02G337200	-2.1538	1.6722	1.42E-12	8.66E-11
TraesCS6A02G223000	-1.1875	7.8651	1.42E-12	8.66E-11
TraesCS7D02G003800	-1.0549	4.5626	1.43E-12	8.70E-11
TraesCS2A02G108600	-3.1628	1.4687	1.49E-12	9.07E-11
TraesCS5D02G488700	2.3762	3.5779	1.50E-12	9.10E-11
TraesCS3D02G003200	-2.6156	1.1225	1.82E-12	1.10E-10
TraesCSU02G107200	-1.3767	2.8995	1.89E-12	1.14E-10
TraesCS6B02G196600	1.0484	4.5853	1.92E-12	1.16E-10
TraesCSU02G166900	-6.8365	-0.1193	1.95E-12	1.17E-10
TraesCS7D02G384600	-1.4071	3.3874	1.98E-12	1.19E-10
TraesCS4D02G317800	-1.2884	4.0907	1.99E-12	1.19E-10
TraesCS2B02G346900	-6.9303	-0.0439	2.00E-12	1.20E-10
TraesCS2B02G204900	-1.763	2.1679	2.00E-12	1.20E-10
TraesCS6D02G399500	-1.0331	4.3225	2.03E-12	1.21E-10
TraesCS1A02G198600	-1.0511	6.6787	2.05E-12	1.22E-10
TraesCS6D02G054900	-1.1049	6.5068	2.07E-12	1.23E-10
TraesCS5D02G312100	1.0829	4.7812	2.11E-12	1.26E-10
TraesCS7A02G270800	-1.1067	4.3999	2.40E-12	1.42E-10
TraesCS6D02G287100	-1.2107	4.3228	2.51E-12	1.49E-10
TraesCS6D02G306600	-1.5255	2.4909	2.56E-12	1.51E-10
TraesCS2A02G200200	-7.2239	0.2036	2.57E-12	1.51E-10
TraesCS2B02G014900	-2.8635	1.3201	2.76E-12	1.62E-10
TraesCS7D02G079800	-3.6592	0.524	2.78E-12	1.63E-10
TraesCS6B02G091500	-1.1112	3.9012	2.78E-12	1.63E-10
TraesCS5B02G227600	-1.9715	3.0972	2.84E-12	1.67E-10
TraesCS6B02G074200	4.074	0.2339	2.86E-12	1.68E-10
TraesCS2B02G284900	-1.3987	4.0018	2.92E-12	1.71E-10
TraesCS2B02G178100	-1.0699	4.0481	2.93E-12	1.71E-10
TraesCS5D02G157300	-1.2485	4.5779	2.96E-12	1.73E-10
TraesCS5D02G287800	-1.034	6.4787	3.04E-12	1.77E-10
TraesCS2A02G033700	1.4164	3.4424	3.13E-12	1.82E-10
TraesCS3D02G224000	-1.5446	2.4935	3.28E-12	1.90E-10
TraesCS5B02G152400	2.4384	1.6603	3.29E-12	1.91E-10

TraesCS3D02G361300	-1.0156	5.4328	3.36E-12	1.94E-10
TraesCS1D02G037300	-6.8032	-0.1456	3.37E-12	1.95E-10
TraesCSU02G235600	-1.602	2.4322	3.47E-12	2.00E-10
TraesCS5A02G264000	-1.3479	3.6301	3.51E-12	2.02E-10
TraesCS6B02G031300	-1.5686	3.4326	3.54E-12	2.04E-10
TraesCS7A02G355800	-1.042	4.2679	3.72E-12	2.13E-10
TraesCS2A02G262400	-1.2858	4.952	3.93E-12	2.25E-10
TraesCS7D02G363600	2.2045	1.6194	4.00E-12	2.28E-10
TraesCS4B02G263300	1.0915	5.5962	4.03E-12	2.30E-10
TraesCS2B02G157800	-3.8382	0.4174	4.12E-12	2.34E-10
TraesCS6D02G316600	1.144	4.291	4.29E-12	2.44E-10
TraesCS6D02G008900	-1.7115	2.717	4.32E-12	2.46E-10
TraesCS6A02G023200	-1.0166	6.0187	4.55E-12	2.58E-10
TraesCS5B02G181700	6.833	-0.1461	4.61E-12	2.61E-10
TraesCS4B02G050400	-1.1856	6.1088	4.73E-12	2.68E-10
TraesCS5A02G138700	-1.2495	6.2959	4.94E-12	2.79E-10
TraesCS3A02G187700	-6.8028	-0.1458	4.97E-12	2.80E-10
TraesCS2D02G362300	-1.4368	3.3975	5.07E-12	2.86E-10
TraesCS4D02G302800	1.9434	2.2578	5.17E-12	2.91E-10
TraesCS2D02G180500	-1.5623	3.08	5.20E-12	2.92E-10
TraesCS6D02G100200	1.4203	3.4484	5.21E-12	2.92E-10
TraesCS5D02G351800	-2.6001	1.0316	5.23E-12	2.93E-10
TraesCS4B02G193700	1.7773	2.1272	5.69E-12	3.18E-10
TraesCS4D02G177300	3.5596	0.7839	5.71E-12	3.19E-10
TraesCS4A02G136600	-1.6457	2.4332	5.73E-12	3.19E-10
TraesCSU02G024600	-1.9879	2.4532	5.79E-12	3.22E-10
TraesCS2A02G110300	1.1705	5.7917	5.87E-12	3.27E-10
TraesCS4D02G280400	-1.1257	6.5224	5.93E-12	3.29E-10
TraesCS4B02G187500	-1.4565	2.9244	6.24E-12	3.46E-10
TraesCS6D02G157600	1.2067	4.1629	6.38E-12	3.53E-10
TraesCS7D02G210500	-1.2527	4.1495	6.44E-12	3.56E-10
TraesCS2D02G393700	1.1359	5.9205	6.68E-12	3.69E-10
TraesCS3D02G310200	1.0276	4.929	6.68E-12	3.69E-10
TraesCS5D02G280400	-1.0758	5.9684	6.79E-12	3.74E-10
TraesCS1D02G158400	-1.2925	3.0503	6.90E-12	3.79E-10
TraesCS2B02G003400	-5.074	0.0048	6.90E-12	3.79E-10
TraesCS3B02G011600	-3.3716	0.5094	7.31E-12	4.01E-10
TraesCS4B02G050600	-1.0104	5.8306	7.31E-12	4.01E-10
TraesCS5D02G012300	1.0867	3.8926	7.55E-12	4.13E-10
TraesCS2B02G075500	-1.3531	3.4533	7.82E-12	4.25E-10
TraesCS4D02G317400	2.9522	0.9664	8.21E-12	4.45E-10
TraesCS5D02G110100	-1.2217	3.5531	8.38E-12	4.53E-10
TraesCS7A02G219700	-5.1039	0.028	8.44E-12	4.56E-10
TraesCSU02G242600	-1.0884	5.3535	8.47E-12	4.57E-10
TraesCS7B02G145200	1.25	4.0767	8.49E-12	4.58E-10
TraesCS5B02G165000	1.2507	3.9749	8.86E-12	4.77E-10
TraesCS6A02G298400	3.3673	0.4567	8.86E-12	4.77E-10
TraesCS4D02G321800	-1.2953	3.1595	8.93E-12	4.79E-10

TraesCS4D02G265100	1.4617	3.44	9.11E-12	4.88E-10
TraesCS6D02G092200	-1.032	6.8915	9.18E-12	4.91E-10
TraesCS4A02G000600	-1.3107	2.9789	9.19E-12	4.91E-10
TraesCS2B02G180400	1.1484	3.8296	9.31E-12	4.97E-10
TraesCS7B02G031400	-2.0297	2.0794	9.36E-12	4.99E-10
TraesCS3D02G368800	1.6316	2.6376	9.41E-12	5.01E-10
TraesCS5B02G103300	-1.3741	3.1607	9.51E-12	5.05E-10
TraesCS4B02G017100	-1.2752	3.4782	9.51E-12	5.05E-10
TraesCS2A02G062200	-1.1505	5.5011	9.68E-12	5.13E-10
TraesCS1B02G024200	-6.8045	-0.1453	9.89E-12	5.23E-10
TraesCS4B02G223300	-1.0463	3.9182	1.03E-11	5.46E-10
TraesCSU02G126000	-2.3601	1.7449	1.07E-11	5.66E-10
TraesCS2D02G018800	-1.2616	3.0673	1.08E-11	5.71E-10
TraesCS2A02G035300	2.1399	1.3934	1.10E-11	5.79E-10
TraesCS1D02G061600	-3.391	1.2739	1.10E-11	5.79E-10
TraesCS2D02G260000	-1.2136	4.2657	1.11E-11	5.83E-10
TraesCS6D02G373400	-1.0966	3.6393	1.13E-11	5.90E-10
TraesCS7D02G353800	1.845	2.329	1.15E-11	6.03E-10
TraesCS2A02G104100	-1.2744	3.3632	1.20E-11	6.27E-10
TraesCS2D02G217100	-1.3814	2.8382	1.20E-11	6.27E-10
TraesCS4D02G226700	-1.0779	4.5621	1.24E-11	6.47E-10
TraesCS7A02G211200	-1.6024	3.4288	1.26E-11	6.57E-10
TraesCS1D02G215100	-1.0431	6.1124	1.30E-11	6.73E-10
TraesCS1D02G296500	1.448	3.0431	1.42E-11	7.35E-10
TraesCS5A02G305100	1.0018	4.8287	1.44E-11	7.48E-10
TraesCS1A02G331800	-1.3282	3.617	1.45E-11	7.48E-10
TraesCS6D02G130500	-1.022	7.245	1.47E-11	7.60E-10
TraesCS2A02G102200	-1.3965	2.7139	1.50E-11	7.73E-10
TraesCS5D02G365800	2.4544	1.7436	1.52E-11	7.83E-10
TraesCS7D02G108900	-1.058	6.7985	1.62E-11	8.31E-10
TraesCS4D02G189200	-2.4139	1.7505	1.65E-11	8.46E-10
TraesCS5B02G269600	-1.1328	3.7855	1.65E-11	8.47E-10
TraesCS7D02G157800	-1.2217	3.6243	1.74E-11	8.89E-10
TraesCS5D02G401300	-1.6272	2.3228	1.76E-11	9.01E-10
TraesCS2B02G318900	-3.1455	0.8158	1.82E-11	9.31E-10
TraesCS5A02G307200	1.4554	2.837	1.87E-11	9.51E-10
TraesCS4D02G276400	1.0154	6.4043	1.87E-11	9.54E-10
TraesCS1D02G387100	-1.333	3.2509	1.97E-11	1.00E-09
TraesCS2D02G295700	-1.1744	5.4692	1.98E-11	1.00E-09
TraesCS6A02G115400	1.0521	3.9512	2.02E-11	1.02E-09
TraesCS5B02G138300	-1.1785	5.7621	2.06E-11	1.04E-09
TraesCS4A02G115400	1.5395	2.5736	2.07E-11	1.05E-09
TraesCS1D02G249600	1.8694	1.7787	2.11E-11	1.07E-09
TraesCS3B02G261800	-1.5766	2.4319	2.12E-11	1.07E-09
TraesCS2D02G061000	-1.0118	5.249	2.13E-11	1.07E-09
TraesCS6B02G159600	1.5201	2.8565	2.22E-11	1.12E-09
TraesCS2B02G158000	-2.1725	1.4686	2.27E-11	1.14E-09
TraesCS7A02G215700	-1.1294	5.4315	2.29E-11	1.15E-09

TraesCS5D02G273300	-1.0262	4.2942	2.30E-11	1.15E-09
TraesCS1D02G133200	-1.1139	3.5944	2.31E-11	1.16E-09
TraesCS4D02G145500	4.3789	0.0541	2.34E-11	1.17E-09
TraesCS5B02G030500	-2.0956	1.402	2.40E-11	1.20E-09
TraesCS5B02G101300	-1.305	3.302	2.46E-11	1.23E-09
TraesCS1B02G250900	1.1982	3.8448	2.47E-11	1.23E-09
TraesCS7D02G273400	-1.4758	2.5828	2.54E-11	1.26E-09
TraesCS7D02G208500	-1.1362	5.8138	2.57E-11	1.28E-09
TraesCS3D02G031400	-1.1799	3.6243	2.63E-11	1.30E-09
TraesCS4A02G190700	1.1945	3.8282	2.81E-11	1.39E-09
TraesCS5A02G304400	1.1353	4.5658	2.90E-11	1.43E-09
TraesCS1B02G233600	1.2522	3.3072	2.96E-11	1.46E-09
TraesCS2A02G062600	-1.1937	6.3537	3.00E-11	1.48E-09
TraesCS2D02G186600	-2.0313	1.9049	3.05E-11	1.50E-09
TraesCS1A02G226000	-2.1721	1.5014	3.12E-11	1.53E-09
TraesCS7A02G191400	-1.2868	3.654	3.15E-11	1.54E-09
TraesCS5B02G051300	-1.1758	3.1347	3.18E-11	1.55E-09
TraesCS6D02G253800	-1.4823	2.6747	3.50E-11	1.71E-09
TraesCS4D02G143900	-1.1085	3.9699	3.51E-11	1.71E-09
TraesCS1B02G200600	1.4263	4.2672	3.68E-11	1.79E-09
TraesCS3B02G198200	-1.6273	2.5601	3.73E-11	1.81E-09
TraesCS1A02G177500	-1.2336	4.277	3.76E-11	1.82E-09
TraesCS7D02G205900	-4.3943	0.1196	3.80E-11	1.84E-09
TraesCS5D02G468000	-1.2218	4.2069	3.96E-11	1.92E-09
TraesCS2A02G153300	-1.4368	2.5351	4.02E-11	1.94E-09
TraesCS6A02G245100	-1.0394	5.3925	4.04E-11	1.95E-09
TraesCS2D02G344200	1.1382	4.4044	4.08E-11	1.97E-09
TraesCS1A02G076700	-2.5495	1.0725	4.14E-11	2.00E-09
TraesCS4D02G055500	1.4018	2.6201	4.42E-11	2.13E-09
TraesCS2B02G029200	4.5349	0.1858	4.55E-11	2.19E-09
TraesCS7D02G273500	-1.4626	2.8021	4.59E-11	2.20E-09
TraesCS1A02G070500	2.3594	1.8085	4.93E-11	2.36E-09
TraesCS6D02G233800	-1.3695	2.8229	4.96E-11	2.37E-09
TraesCS7D02G192700	-1.7338	2.3324	5.02E-11	2.39E-09
TraesCS1D02G195300	-1.1978	3.2722	5.03E-11	2.40E-09
TraesCS5D02G228200	-1.5008	3.5909	5.05E-11	2.40E-09
TraesCS7D02G348600	1.2633	3.4204	5.09E-11	2.42E-09
TraesCS2D02G033200	1.363	2.7774	5.15E-11	2.45E-09
TraesCS5A02G073700	-1.3931	4.2785	5.27E-11	2.50E-09
TraesCS2D02G081000	3.2558	0.3697	5.31E-11	2.51E-09
TraesCS5A02G301100	2.1687	1.4566	5.40E-11	2.55E-09
TraesCS6A02G071000	1.8032	2.7219	5.41E-11	2.55E-09
TraesCS2B02G124600	-1.756	1.8073	5.42E-11	2.56E-09
TraesCS5D02G277500	-1.0188	4.0519	5.66E-11	2.66E-09
TraesCS7B02G061600	-1.2201	4.1152	5.69E-11	2.67E-09
TraesCS3B02G080600	1.2229	4.4495	5.79E-11	2.72E-09
TraesCS4B02G127100	-1.061	4.035	5.86E-11	2.75E-09
TraesCS2D02G145400	-1.1255	5.607	5.94E-11	2.78E-09

TraesCS5D02G058900	-1.2487	2.9152	6.02E-11	2.82E-09
TraesCS6D02G298300	1.1835	6.306	6.46E-11	3.02E-09
TraesCSU02G040800	-1.1601	3.609	6.55E-11	3.06E-09
TraesCS7B02G277800	-1.3908	2.6241	6.61E-11	3.08E-09
TraesCS1D02G395200	-1.3355	2.9732	6.62E-11	3.08E-09
TraesCS2A02G104600	1.2695	5.4829	6.62E-11	3.08E-09
TraesCS7A02G273400	-1.4994	2.4509	6.66E-11	3.10E-09
TraesCS3B02G200200	-1.0677	4.9586	6.67E-11	3.10E-09
TraesCS6D02G276500	1.1157	3.8865	6.70E-11	3.11E-09
TraesCS7D02G357900	1.5008	2.6534	6.70E-11	3.11E-09
TraesCS3D02G092800	-1.1817	3.2049	6.72E-11	3.11E-09
TraesCS1A02G327500	5.004	-0.0936	6.84E-11	3.16E-09
TraesCS4D02G238700	-3.8155	0.6518	7.08E-11	3.27E-09
TraesCS1B02G195000	-1.9135	1.7327	7.15E-11	3.30E-09
TraesCS2B02G032000	1.9222	1.4976	7.23E-11	3.33E-09
TraesCS2B02G356100	1.1413	3.6674	7.25E-11	3.33E-09
TraesCS3D02G114700	2.9903	0.8842	7.30E-11	3.35E-09
TraesCS6D02G284200	1.3723	2.975	7.52E-11	3.45E-09
TraesCS2D02G016700	-1.0385	5.7295	7.73E-11	3.54E-09
TraesCS5B02G300300	1.3748	5.1251	8.01E-11	3.66E-09
TraesCS4B02G167100	-1.3222	3.4935	8.02E-11	3.66E-09
TraesCS5B02G212000	1.0284	4.003	8.03E-11	3.66E-09
TraesCS1D02G034800	-1.4084	4.5955	8.16E-11	3.71E-09
TraesCS6D02G158100	1.2701	3.1458	8.33E-11	3.79E-09
TraesCS7B02G062500	2.5523	1.1022	8.46E-11	3.84E-09
TraesCS1D02G266000	1.1687	5.4033	8.84E-11	4.01E-09
TraesCS3D02G271000	-7.3344	0.288	8.95E-11	4.06E-09
TraesCS2D02G378900	-2.7509	0.7553	9.09E-11	4.11E-09
TraesCS6D02G158200	1.2582	3.0995	9.23E-11	4.17E-09
TraesCS5D02G498400	-1.1425	4.2424	9.29E-11	4.19E-09
TraesCS6D02G014100	-6.7039	-0.2261	9.37E-11	4.23E-09
TraesCS2A02G051700	-3.186	0.3665	9.39E-11	4.23E-09
TraesCSU02G242700	-1.0588	5.2865	9.62E-11	4.33E-09
TraesCS2B02G329900	6.618	-0.3137	1.01E-10	4.55E-09
TraesCS4A02G076700	-1.4486	2.5191	1.02E-10	4.57E-09
TraesCS6A02G047200	-2.9168	0.6394	1.02E-10	4.58E-09
TraesCS1A02G299100	1.3278	3.248	1.03E-10	4.61E-09
TraesCS2B02G303200	2.7266	0.8928	1.03E-10	4.62E-09
TraesCS1A02G350900	1.3504	2.9535	1.04E-10	4.63E-09
TraesCS6A02G244900	-1.1333	4.74	1.06E-10	4.70E-09
TraesCS1A02G238800	1.3238	3.906	1.06E-10	4.70E-09
TraesCS1D02G032500	-1.4478	3.0636	1.08E-10	4.80E-09
TraesCS5D02G229700	-1.2596	3.3779	1.08E-10	4.81E-09
TraesCS7A02G185300	-2.2647	1.0776	1.10E-10	4.89E-09
TraesCS7B02G267300	6.6239	-0.3113	1.11E-10	4.89E-09
TraesCS5D02G288900	1.6626	3.0902	1.15E-10	5.06E-09
TraesCS2A02G025700	2.6741	0.756	1.15E-10	5.06E-09
TraesCS5B02G123100	1.8238	4.2778	1.16E-10	5.09E-09

TraesCS7D02G319700	-1.1576	3.3926	1.20E-10	5.26E-09
TraesCS1B02G204100	-1.6654	2.471	1.20E-10	5.28E-09
TraesCS1D02G114500	-1.1686	3.313	1.24E-10	5.44E-09
TraesCS5A02G004800	1.0164	4.6724	1.28E-10	5.63E-09
TraesCS2A02G061800	-1.0108	5.7471	1.29E-10	5.64E-09
TraesCS5D02G507800	1.5392	2.2227	1.30E-10	5.67E-09
TraesCS2B02G167100	-1.0365	5.2782	1.34E-10	5.86E-09
TraesCS6B02G207200	-1.3633	2.918	1.50E-10	6.51E-09
TraesCS2A02G235500	-2.8372	0.5746	1.54E-10	6.70E-09
TraesCSU02G253900	1.8002	1.821	1.55E-10	6.71E-09
TraesCS6A02G084500	-2.0077	1.4726	1.57E-10	6.81E-09
TraesCS5D02G441200	-1.9838	1.9388	1.59E-10	6.87E-09
TraesCS3B02G264000	-1.1947	3.8609	1.61E-10	6.96E-09
TraesCS3B02G258500	-1.358	2.4559	1.63E-10	7.04E-09
TraesCS4D02G303000	1.6635	2.4365	1.69E-10	7.28E-09
TraesCS3A02G213500	-1.9156	1.9777	1.78E-10	7.68E-09
TraesCS2B02G352000	-1.0568	4.0933	1.88E-10	8.09E-09
TraesCS2D02G290400	-1.2754	4.9421	1.88E-10	8.09E-09
TraesCS3A02G288800	1.7088	2.2781	1.90E-10	8.12E-09
TraesCS4D02G328400	-1.9803	2.7792	1.96E-10	8.37E-09
TraesCS7B02G152800	-1.1001	4.4303	1.97E-10	8.40E-09
TraesCS1A02G339700	1.5595	4.7736	2.00E-10	8.52E-09
TraesCS5B02G014500	-1.4981	2.5432	2.06E-10	8.77E-09
TraesCS2A02G295100	-6.8624	-0.095	2.08E-10	8.83E-09
TraesCS7A02G057700	3.0457	0.9445	2.19E-10	9.28E-09
TraesCS1D02G154300	1.8893	1.3892	2.27E-10	9.61E-09
TraesCS5D02G318900	1.2734	2.6797	2.28E-10	9.61E-09
TraesCS5B02G313000	1.2553	3.7636	2.28E-10	9.63E-09
TraesCS7D02G207600	-1.1227	3.305	2.34E-10	9.86E-09
TraesCS1D02G334400	-1.3055	3.2472	2.38E-10	9.99E-09
TraesCS7D02G126400	1.9167	1.2719	2.38E-10	1.00E-08
TraesCS2B02G278200	-1.364	3.3215	2.44E-10	1.02E-08
TraesCSU02G083100	-1.0472	4.5631	2.44E-10	1.02E-08
TraesCS1D02G367400	-2.0915	1.1958	2.44E-10	1.02E-08
TraesCS7D02G351300	1.1162	5.1574	2.45E-10	1.02E-08
TraesCS7A02G106900	-2.4103	1.3097	2.52E-10	1.05E-08
TraesCS3A02G246600	-1.2206	5.0996	2.56E-10	1.06E-08
TraesCS6A02G255400	1.3549	2.7092	2.59E-10	1.08E-08
TraesCS4B02G023200	1.8867	2.1357	2.61E-10	1.08E-08
TraesCS7B02G227800	-1.0039	5.2634	2.61E-10	1.08E-08
TraesCS6A02G177500	-1.0936	3.2298	2.70E-10	1.12E-08
TraesCS6A02G086300	1.6986	7.6356	2.76E-10	1.14E-08
TraesCS4B02G199700	1.4978	3.1502	2.76E-10	1.14E-08
TraesCS6D02G375700	3.846	0.0574	2.77E-10	1.14E-08
TraesCS6A02G185100	-1.1071	3.5724	2.80E-10	1.15E-08
TraesCSU02G140200	-6.6866	-0.2312	2.90E-10	1.19E-08
TraesCS7D02G378300	-1.823	1.5079	2.90E-10	1.19E-08
TraesCS3A02G239200	1.5681	2.4952	2.90E-10	1.19E-08

TraesCS2A02G155300	1.1602	4.8186	2.99E-10	1.22E-08
TraesCS1D02G138300	-6.542	-0.3462	3.05E-10	1.24E-08
TraesCS4B02G005000	-3.2193	0.5732	3.18E-10	1.30E-08
TraesCS2D02G396400	-1.1483	3.8915	3.30E-10	1.35E-08
TraesCS2B02G201400	-1.446	2.4825	3.32E-10	1.35E-08
TraesCS5D02G137500	-1.0781	5.4327	3.36E-10	1.37E-08
TraesCS5D02G470800	1.414	2.9211	3.38E-10	1.37E-08
TraesCS5A02G213300	-1.1322	3.3147	3.38E-10	1.37E-08
TraesCS1D02G266100	1.2419	3.9862	3.44E-10	1.40E-08
TraesCS1A02G221600	-1.0643	3.6498	3.45E-10	1.40E-08
TraesCS5D02G273700	-1.1012	3.4872	3.52E-10	1.43E-08
TraesCS2D02G165800	1.2529	3.3696	3.57E-10	1.44E-08
TraesCS1D02G116000	-1.1506	3.0964	3.59E-10	1.45E-08
TraesCS2D02G274600	-1.5202	1.9848	3.61E-10	1.45E-08
TraesCS7D02G233300	2.7802	0.6103	3.74E-10	1.51E-08
TraesCS5B02G220700	-1.3693	2.6641	3.90E-10	1.57E-08
TraesCS7B02G001700	-1.1963	3.2297	3.91E-10	1.57E-08
TraesCS3A02G200300	-1.6526	2.3878	3.95E-10	1.58E-08
TraesCS4D02G114400	-1.816	1.5428	4.04E-10	1.61E-08
TraesCS3D02G416700	6.5804	-0.3431	4.15E-10	1.66E-08
TraesCS7D02G048000	-1.2232	3.435	4.48E-10	1.79E-08
TraesCS1A02G021700	-6.5784	-0.3173	4.49E-10	1.79E-08
TraesCS5B02G197000	-1.9336	1.1859	4.50E-10	1.79E-08
TraesCS7B02G207400	-1.0918	3.305	4.67E-10	1.85E-08
TraesCS3D02G244800	1.7766	2.0238	4.68E-10	1.85E-08
TraesCS2B02G047500	2.1935	1.6517	4.75E-10	1.88E-08
TraesCS3B02G164500	-1.0644	3.9587	4.82E-10	1.90E-08
TraesCS2B02G097600	6.5906	-0.3403	4.82E-10	1.90E-08
TraesCSU02G136000	-6.5817	-0.316	4.83E-10	1.90E-08
TraesCS6D02G069200	-6.6578	-0.2574	4.96E-10	1.95E-08
TraesCS3B02G130500	3.3527	0.2335	5.18E-10	2.03E-08
TraesCS5A02G133900	-1.0061	3.9327	5.27E-10	2.07E-08
TraesCS3D02G067200	1.0548	5.2192	5.36E-10	2.10E-08
TraesCS1D02G217600	1.5256	5.1033	5.55E-10	2.17E-08
TraesCS2D02G328200	-1.0366	5.6634	5.61E-10	2.19E-08
TraesCS2D02G352500	-1.2168	4.0205	5.76E-10	2.25E-08
TraesCS4A02G104300	1.5444	2.3464	5.91E-10	2.31E-08
TraesCS5D02G491100	1.8887	1.7603	6.12E-10	2.38E-08
TraesCS2B02G172300	1.6669	1.7821	6.29E-10	2.44E-08
TraesCS1A02G319300	-1.1426	3.2147	6.31E-10	2.45E-08
TraesCS7D02G217400	-1.075	5.4044	6.36E-10	2.46E-08
TraesCS5B02G074400	1.1569	5.6502	6.45E-10	2.49E-08
TraesCS6B02G115900	1.6815	7.2485	6.46E-10	2.49E-08
TraesCS1A02G090400	-2.6725	1.0834	6.58E-10	2.54E-08
TraesCS3D02G225800	1.6246	1.8416	6.64E-10	2.56E-08
TraesCS7D02G004700	-1.3003	2.7128	6.73E-10	2.59E-08
TraesCS2A02G215200	1.0383	4.2403	6.79E-10	2.61E-08
TraesCS2A02G205700	-1.0338	3.8854	7.10E-10	2.73E-08

TraesCS3A02G165500	-1.1271	4.4717	7.27E-10	2.78E-08
TraesCS7D02G160700	-3.8911	0.1412	7.55E-10	2.89E-08
TraesCS5A02G178300	-1.0582	3.9584	7.68E-10	2.93E-08
TraesCS5B02G245800	1.4767	2.6156	7.76E-10	2.96E-08
TraesCS5A02G214200	-1.3338	2.512	8.18E-10	3.12E-08
TraesCS4D02G261300	-1.7082	1.8268	8.50E-10	3.23E-08
TraesCS7A02G196800	-2.3161	1.0427	8.57E-10	3.25E-08
TraesCS7B02G132600	-1.3776	2.501	8.79E-10	3.33E-08
TraesCS7D02G130400	-2.0213	1.9897	8.82E-10	3.34E-08
TraesCS2A02G292600	-1.1547	4.5127	8.99E-10	3.40E-08
TraesCS6D02G300600	2.2121	0.9876	9.15E-10	3.46E-08
TraesCS3D02G293600	-1.9666	1.2594	9.31E-10	3.51E-08
TraesCS2A02G118600	-1.7725	2.6327	9.36E-10	3.52E-08
TraesCS6B02G118000	-1.669	2.396	9.51E-10	3.58E-08
TraesCS6B02G268100	1.4113	2.301	9.70E-10	3.65E-08
TraesCS1B02G311800	-1.1808	2.8485	9.71E-10	3.65E-08
TraesCS7A02G084600	-1.7386	1.5965	9.83E-10	3.69E-08
TraesCS4D02G025700	1.3947	6.6734	9.84E-10	3.69E-08
TraesCS7D02G161000	1.1368	7.376	1.02E-09	3.81E-08
TraesCS2B02G341100	-1.3391	2.6988	1.03E-09	3.83E-08
TraesCS3B02G163300	-1.1396	4.9602	1.03E-09	3.84E-08
TraesCS3D02G071900	-6.5813	-0.3161	1.03E-09	3.84E-08
TraesCS1B02G230100	-1.5713	2.5939	1.03E-09	3.84E-08
TraesCS7A02G361000	1.0343	4.0807	1.04E-09	3.86E-08
TraesCS5A02G017100	-1.6367	1.7466	1.05E-09	3.89E-08
TraesCS6D02G136300	-6.7507	-0.1764	1.06E-09	3.94E-08
TraesCS3D02G381500	-1.6258	1.8236	1.08E-09	4.00E-08
TraesCS5D02G314100	1.7513	1.9368	1.10E-09	4.08E-08
TraesCS3D02G244900	-1.1657	5.7943	1.11E-09	4.09E-08
TraesCS1D02G061000	-4.1645	-0.0673	1.15E-09	4.24E-08
TraesCS4A02G147700	-2.7403	0.622	1.15E-09	4.25E-08
TraesCS6B02G246400	1.6019	3.3781	1.17E-09	4.30E-08
TraesCS2A02G033000	-1.308	2.3874	1.18E-09	4.32E-08
TraesCS1D02G028400	2.9692	0.3328	1.20E-09	4.41E-08
TraesCS7D02G187000	-2.194	1.0884	1.24E-09	4.56E-08
TraesCS3B02G107900	-1.0958	3.1637	1.25E-09	4.57E-08
TraesCS6D02G208900	2.2123	0.9886	1.28E-09	4.68E-08
TraesCS2D02G358400	-1.1241	4.0244	1.28E-09	4.69E-08
TraesCSU02G036600	-2.5751	0.6227	1.30E-09	4.76E-08
TraesCS7A02G329300	-1.0134	3.7574	1.31E-09	4.77E-08
TraesCS7A02G062600	-2.8884	0.9734	1.32E-09	4.81E-08
TraesCS3D02G354200	-1.1024	5.4611	1.33E-09	4.83E-08
TraesCS4B02G197500	-1.1559	4.1438	1.35E-09	4.90E-08
TraesCSU02G132300	1.3106	5.2449	1.37E-09	4.95E-08
TraesCS7A02G051100	1.6646	2.4569	1.37E-09	4.95E-08
TraesCS6D02G137000	-1.2103	3.0888	1.38E-09	5.00E-08
TraesCS5B02G270500	1.3346	2.5283	1.39E-09	5.03E-08
TraesCS4A02G200900	1.4219	3.041	1.40E-09	5.04E-08

TraesCS6D02G212300	-1.0742	7.3951	1.40E-09	5.06E-08
TraesCS3D02G128500	1.196	3.395	1.41E-09	5.09E-08
TraesCS6D02G285600	-1.1167	3.0537	1.46E-09	5.26E-08
TraesCS1D02G073300	-1.4696	2.4483	1.56E-09	5.59E-08
TraesCS1D02G418700	1.0522	4.2612	1.56E-09	5.60E-08
TraesCS5B02G177200	1.3444	3.9229	1.58E-09	5.64E-08
TraesCS5A02G198800	1.2617	3.0837	1.63E-09	5.82E-08
TraesCS5A02G008800	-2.4029	1.297	1.66E-09	5.93E-08
TraesCS5A02G219500	1.6449	1.961	1.67E-09	5.95E-08
TraesCS6D02G220700	2.3893	1.8553	1.71E-09	6.09E-08
TraesCS2B02G126200	-1.1146	4.1069	1.80E-09	6.39E-08
TraesCS3D02G149000	1.3355	5.9679	1.82E-09	6.45E-08
TraesCS5D02G156500	-1.3478	2.3126	1.83E-09	6.49E-08
TraesCS7A02G208100	-1.0647	3.9537	1.83E-09	6.49E-08
TraesCS1A02G212400	-1.1632	5.5609	1.85E-09	6.57E-08
TraesCS5D02G396200	1.1328	3.1396	1.88E-09	6.65E-08
TraesCSU02G007900	1.4402	2.2584	1.92E-09	6.79E-08
TraesCS2D02G185800	-1.061	3.3186	2.03E-09	7.16E-08
TraesCS2D02G285600	-1.2212	3.2208	2.05E-09	7.21E-08
TraesCSU02G144500	-1.085	5.4815	2.05E-09	7.21E-08
TraesCS4D02G227700	-1.1674	4.0252	2.11E-09	7.41E-08
TraesCS7D02G127600	1.1377	3.5852	2.12E-09	7.43E-08
TraesCS7D02G176500	3.9615	0.1623	2.18E-09	7.65E-08
TraesCS4D02G359800	1.2903	2.4302	2.19E-09	7.67E-08
TraesCS2B02G309000	-1.406	4.321	2.20E-09	7.68E-08
TraesCS5D02G204700	1.1509	3.3478	2.20E-09	7.68E-08
TraesCS2A02G264000	-1.5758	2.569	2.20E-09	7.69E-08
TraesCS3B02G317000	-1.1533	5.3256	2.31E-09	8.03E-08
TraesCS3A02G114900	-1.8701	1.8388	2.31E-09	8.04E-08
TraesCS2A02G035100	1.2787	8.1756	2.34E-09	8.12E-08
TraesCS1D02G017500	-1.505	2.7603	2.45E-09	8.50E-08
TraesCS3A02G301200	2.6454	0.6259	2.46E-09	8.52E-08
TraesCS7D02G271900	1.7978	2.6331	2.48E-09	8.59E-08
TraesCS2D02G186900	-1.7419	2.8415	2.51E-09	8.66E-08
TraesCS6D02G304300	-1.109	3.4296	2.53E-09	8.74E-08
TraesCS3A02G038200	-1.1539	3.7814	2.55E-09	8.80E-08
TraesCS4B02G112500	1.0101	3.6389	2.60E-09	8.96E-08
TraesCS2B02G236500	-6.4932	-0.3794	2.73E-09	9.39E-08
TraesCS5D02G139600	-2.2922	0.8771	2.75E-09	9.44E-08
TraesCS6B02G255400	1.3456	2.5236	2.80E-09	9.59E-08
TraesCS6D02G165200	-2.1209	1.1639	2.82E-09	9.66E-08
TraesCS1D02G405200	2.0649	1.2377	2.82E-09	9.66E-08
TraesCS3B02G110600	-1.7068	2.3165	2.82E-09	9.66E-08
TraesCS4D02G004200	-6.37	-0.4738	2.95E-09	1.01E-07
TraesCS7A02G131000	-1.8975	2.2695	3.01E-09	1.03E-07
TraesCS6A02G265800	-1.038	3.5509	3.02E-09	1.03E-07
TraesCS3B02G000200	10.5411	3.2286	3.07E-09	1.04E-07
TraesCS4B02G188100	-1.0244	3.568	3.07E-09	1.04E-07

TraesCS3B02G145500	1.0945	3.0609	3.08E-09	1.04E-07
TraesCS2B02G251700	-1.2155	2.8139	3.11E-09	1.05E-07
TraesCS1D02G372700	1.3158	2.9755	3.12E-09	1.06E-07
TraesCS3A02G278000	-1.8612	1.6208	3.22E-09	1.09E-07
TraesCS1B02G274300	1.2189	3.3069	3.22E-09	1.09E-07
TraesCS6B02G064700	-1.2653	3.026	3.30E-09	1.11E-07
TraesCS2B02G037600	-1.6757	1.9147	3.31E-09	1.11E-07
TraesCS4A02G071100	-1.4854	2.3834	3.38E-09	1.14E-07
TraesCS1A02G283400	-1.2334	3.2765	3.44E-09	1.16E-07
TraesCS5B02G206100	-1.9787	0.9937	3.51E-09	1.18E-07
TraesCS7D02G375800	1.2655	2.5818	3.53E-09	1.18E-07
TraesCS2D02G182400	-1.3645	2.5993	3.63E-09	1.22E-07
TraesCS2B02G125300	-2.7528	0.5077	3.65E-09	1.23E-07
TraesCS7B02G020300	-1	3.9351	3.80E-09	1.27E-07
TraesCS5B02G235700	1.1966	3.5103	3.83E-09	1.28E-07
TraesCS7A02G071600	-1.1046	3.3932	3.85E-09	1.29E-07
TraesCS3A02G221400	-1.9737	1.1082	3.85E-09	1.29E-07
TraesCS5D02G213100	-1.14	3.4326	3.88E-09	1.30E-07
TraesCS1D02G038300	-2.1974	1.083	4.02E-09	1.34E-07
TraesCS1D02G090000	1.2432	2.5571	4.13E-09	1.37E-07
TraesCS1B02G280400	-1.0507	3.2754	4.13E-09	1.37E-07
TraesCS2B02G106000	1.2668	2.6236	4.15E-09	1.38E-07
TraesCS2D02G295500	1.2031	7.7314	4.21E-09	1.40E-07
TraesCS5D02G265700	1.151	3.5442	4.23E-09	1.40E-07
TraesCS4A02G011700	1.0236	3.4056	4.24E-09	1.40E-07
TraesCS1A02G168500	-1.6846	1.5222	4.24E-09	1.40E-07
TraesCS7D02G177000	-1.1688	4.1384	4.24E-09	1.40E-07
TraesCS6D02G228900	-1.9506	1.591	4.25E-09	1.41E-07
TraesCS1D02G352000	1.1889	3.9087	4.46E-09	1.47E-07
TraesCS5D02G307000	1.0257	5.9903	4.57E-09	1.51E-07
TraesCS5A02G030700	-1.8186	1.7793	4.60E-09	1.51E-07
TraesCS5B02G149900	-1.8338	2.1761	4.80E-09	1.58E-07
TraesCS4D02G298400	-1.5765	1.7879	4.85E-09	1.59E-07
TraesCS3D02G235200	-1.1215	4.0624	4.90E-09	1.61E-07
TraesCS3D02G266000	1.8647	1.3252	5.27E-09	1.73E-07
TraesCS4D02G145600	1.8029	1.2816	5.47E-09	1.79E-07
TraesCS6B02G100100	3.3608	0.4694	5.51E-09	1.80E-07
TraesCS7B02G084600	-1.9036	1.3106	5.73E-09	1.87E-07
TraesCS1B02G226000	-1.0203	6.0858	5.75E-09	1.87E-07
TraesCS7B02G072600	1.7219	2.6945	5.81E-09	1.89E-07
TraesCS5B02G071000	-3.7873	0.0556	6.13E-09	1.99E-07
TraesCS1D02G184600	-1.295	4.013	6.19E-09	2.00E-07
TraesCS3A02G276100	-1.73	1.5189	6.19E-09	2.00E-07
TraesCS1B02G287400	1.7879	2.5702	6.57E-09	2.12E-07
TraesCS6A02G034900	-3.5829	-0.0926	6.67E-09	2.15E-07
TraesCS3B02G296100	1.2698	2.995	6.82E-09	2.20E-07
TraesCS2D02G136700	-1.7831	1.3557	6.94E-09	2.24E-07
TraesCS5A02G176600	1.1392	3.4288	6.98E-09	2.25E-07

TraesCS6D02G015100	-1.2631	4.1967	7.14E-09	2.30E-07
TraesCS7A02G225000	1.0578	3.3127	7.17E-09	2.30E-07
TraesCSU02G029800	-1.5917	2.3108	7.40E-09	2.37E-07
TraesCS7A02G271600	1.3063	2.5565	7.67E-09	2.45E-07
TraesCS3A02G022900	-1.1452	3.3272	7.69E-09	2.46E-07
TraesCS2D02G075200	2.6382	0.5086	7.72E-09	2.47E-07
TraesCS5A02G300800	1.1274	5.9214	7.82E-09	2.50E-07
TraesCS6D02G076600	-1.3483	2.331	8.40E-09	2.68E-07
TraesCS2B02G196700	2.2658	0.6414	9.17E-09	2.91E-07
TraesCS2B02G230700	-1.2426	2.9846	9.21E-09	2.93E-07
TraesCS3D02G220800	2.9337	0.3075	9.31E-09	2.95E-07
TraesCS6D02G007400	4.6788	-0.342	9.33E-09	2.96E-07
TraesCS6B02G138700	-1.2012	3.6872	9.36E-09	2.96E-07
TraesCS6D02G238200	1.375	2.3611	9.76E-09	3.09E-07
TraesCS6D02G080200	-1.1408	3.2612	9.78E-09	3.09E-07
TraesCS7D02G025300	-2.433	0.8171	1.00E-08	3.15E-07
TraesCS3A02G168200	-1.3842	2.7528	1.01E-08	3.19E-07
TraesCS3A02G262600	2.4179	0.8429	1.04E-08	3.26E-07
TraesCS3D02G340500	-1.4466	2.965	1.04E-08	3.26E-07
TraesCS1A02G284100	-1.1855	2.6716	1.06E-08	3.32E-07
TraesCS4D02G303300	1.7899	1.8968	1.06E-08	3.33E-07
TraesCS1A02G202700	-6.2736	-0.5432	1.08E-08	3.39E-07
TraesCS6B02G070900	-2.0421	1.0435	1.10E-08	3.43E-07
TraesCS1A02G260700	1.724	3.1623	1.11E-08	3.47E-07
TraesCS2B02G144200	1.3177	3.4749	1.13E-08	3.50E-07
TraesCS3B02G256500	2.0303	1.2133	1.13E-08	3.51E-07
TraesCS4D02G277300	-1.0497	3.0885	1.19E-08	3.70E-07
TraesCS5B02G224000	-1.5069	1.7425	1.20E-08	3.70E-07
TraesCS4A02G039700	1.8953	1.6996	1.21E-08	3.73E-07
TraesCS3A02G127300	1.2831	2.9162	1.22E-08	3.77E-07
TraesCS7B02G082000	1.766	1.9864	1.23E-08	3.79E-07
TraesCS3A02G219100	1.1513	5.117	1.23E-08	3.79E-07
TraesCS5D02G047100	-1.1078	3.1497	1.24E-08	3.83E-07
TraesCS5D02G135500	-2.8272	0.4231	1.28E-08	3.93E-07
TraesCS2B02G177300	1.0609	3.0181	1.28E-08	3.94E-07
TraesCS7D02G084700	-1.7284	2.1366	1.30E-08	3.98E-07
TraesCS4D02G103600	-1.0108	3.8111	1.30E-08	3.98E-07
TraesCS5D02G441300	-1.6488	1.9163	1.30E-08	4.00E-07
TraesCS4D02G235800	-1.1935	2.8919	1.35E-08	4.13E-07
TraesCS1B02G018200	-1.4527	2.5406	1.35E-08	4.14E-07
TraesCS6D02G061700	-1.099	3.0563	1.36E-08	4.16E-07
TraesCS1D02G160400	-4.6508	-0.3172	1.37E-08	4.18E-07
TraesCS6A02G005100	4.8762	-0.1965	1.37E-08	4.18E-07
TraesCS1D02G389200	1.3283	2.355	1.38E-08	4.21E-07
TraesCS4B02G017200	-6.32	-0.5092	1.40E-08	4.28E-07
TraesCS1B02G067800	-4.7257	-0.2599	1.42E-08	4.33E-07
TraesCS1A02G022500	-4.7611	-0.231	1.42E-08	4.33E-07
TraesCS4B02G197100	2.958	0.1455	1.43E-08	4.36E-07

TraesCS6D02G083500	1.0135	4.9848	1.46E-08	4.44E-07
TraesCS5B02G150000	-1.9167	1.7228	1.48E-08	4.49E-07
TraesCSU02G059200	-1.8577	1.5671	1.48E-08	4.49E-07
TraesCS2D02G150400	-1.1082	3.1044	1.53E-08	4.62E-07
TraesCS4B02G169900	-1.1698	2.7751	1.53E-08	4.62E-07
TraesCS5D02G075800	2.943	1.0476	1.53E-08	4.62E-07
TraesCS5D02G427600	-2.1188	1.159	1.56E-08	4.70E-07
TraesCS2D02G209100	1.1925	4.3038	1.59E-08	4.79E-07
TraesCS5A02G167100	2.4033	0.9836	1.59E-08	4.80E-07
TraesCS4D02G359400	-2.3471	0.5567	1.61E-08	4.85E-07
TraesCS6D02G350300	-1.0403	3.9033	1.62E-08	4.86E-07
TraesCS6D02G140100	2.3707	0.5291	1.64E-08	4.92E-07
TraesCSU02G115200	-1.0068	3.2002	1.64E-08	4.93E-07
TraesCS4B02G058200	1.7629	1.8377	1.66E-08	4.98E-07
TraesCS2B02G117500	-1.4759	1.9754	1.72E-08	5.13E-07
TraesCS3A02G226000	-1.1565	2.8167	1.72E-08	5.14E-07
TraesCS7A02G156900	1.6376	4.0319	1.73E-08	5.16E-07
TraesCS6A02G091300	-1.2183	2.7527	1.74E-08	5.19E-07
TraesCS5D02G488300	1.0503	3.5621	1.75E-08	5.23E-07
TraesCS2B02G233000	-1.1907	2.5258	1.76E-08	5.24E-07
TraesCS4A02G025300	-1.0108	3.6618	1.78E-08	5.31E-07
TraesCS3A02G266400	1.4775	2.0081	1.80E-08	5.34E-07
TraesCS2D02G107500	-1.7043	1.7074	1.82E-08	5.40E-07
TraesCS7D02G314000	-1.1764	2.6519	1.90E-08	5.63E-07
TraesCS5B02G224100	-1.6455	2.3053	1.93E-08	5.71E-07
TraesCS5D02G341500	-1.1408	2.9552	1.94E-08	5.73E-07
TraesCS5A02G064500	1.2602	2.2635	1.95E-08	5.76E-07
TraesCS4D02G019500	-1.988	2.6326	2.01E-08	5.93E-07
TraesCSU02G201000	-3.1799	2.105	2.03E-08	5.98E-07
TraesCS3D02G145600	-1.1884	2.7743	2.07E-08	6.10E-07
TraesCS6B02G209600	1.037	4.3502	2.09E-08	6.16E-07
TraesCS5B02G197400	1.4126	2.5662	2.11E-08	6.22E-07
TraesCS7A02G119200	-2.9482	0.3636	2.13E-08	6.27E-07
TraesCS2A02G186300	-1.1707	2.8743	2.14E-08	6.27E-07
TraesCS3A02G246700	1.876	2.3363	2.18E-08	6.39E-07
TraesCS5B02G304800	1.8884	4.6871	2.20E-08	6.46E-07
TraesCS4B02G089800	3.3076	-0.0428	2.21E-08	6.47E-07
TraesCS3D02G256500	1.0641	3.0987	2.24E-08	6.56E-07
TraesCS4B02G175400	1.7388	2.0464	2.29E-08	6.68E-07
TraesCS2D02G284700	1.9736	1.2646	2.29E-08	6.68E-07
TraesCS3A02G283800	1.157	4.208	2.41E-08	7.02E-07
TraesCS2B02G076400	-1.4545	2.3356	2.46E-08	7.14E-07
TraesCS5B02G133700	-1.4339	2.0862	2.47E-08	7.17E-07
TraesCS1B02G304600	1.8078	1.5611	2.54E-08	7.34E-07
TraesCS1D02G008000	-1.3092	2.1245	2.55E-08	7.39E-07
TraesCS5B02G015300	-1.9646	1.1012	2.60E-08	7.51E-07
TraesCS5D02G275200	3.0795	0.0323	2.63E-08	7.60E-07
TraesCS5D02G470200	-1.5878	1.957	2.66E-08	7.65E-07

TraesCS5B02G137300	-1.3281	2.4869	2.70E-08	7.76E-07
TraesCS7D02G268500	-1.1323	2.6546	2.73E-08	7.83E-07
TraesCS1A02G240500	-1.7747	1.4685	2.74E-08	7.87E-07
TraesCS5B02G312700	1.3681	2.1208	2.75E-08	7.88E-07
TraesCS2B02G311100	-1.1754	2.8008	2.75E-08	7.88E-07
TraesCS7B02G075000	3.9779	-0.2527	2.76E-08	7.89E-07
TraesCS3B02G317400	3.2795	-0.0634	2.76E-08	7.90E-07
TraesCS6A02G000200	-4.9244	-0.1191	2.82E-08	8.05E-07
TraesCS3B02G190800	1.8208	1.5404	2.86E-08	8.13E-07
TraesCS7A02G253700	-2.5366	0.5888	2.88E-08	8.19E-07
TraesCS5B02G209500	-1.7451	1.535	2.97E-08	8.40E-07
TraesCS4A02G050000	1.0826	4.0645	3.05E-08	8.62E-07
TraesCS3D02G103800	2.1777	0.7451	3.05E-08	8.63E-07
TraesCS2B02G357200	2.3192	0.4907	3.11E-08	8.77E-07
TraesCS1D02G392100	-1.988	1.27	3.12E-08	8.80E-07
TraesCS2D02G201600	-1.6224	1.6783	3.14E-08	8.86E-07
TraesCS6B02G243900	-1.4235	1.964	3.18E-08	8.94E-07
TraesCS1A02G187300	-1.1456	3.1227	3.24E-08	9.11E-07
TraesCS2D02G212300	-1.0581	3.0866	3.24E-08	9.12E-07
TraesCS3B02G131000	2.7983	0.5076	3.25E-08	9.12E-07
TraesCS6D02G236800	1.4164	2.9469	3.26E-08	9.15E-07
TraesCS6D02G234600	-1.3554	2.0021	3.26E-08	9.15E-07
TraesCS1A02G192200	1.3073	4.1483	3.35E-08	9.39E-07
TraesCS2B02G236200	-1.9056	1.1518	3.36E-08	9.41E-07
TraesCS1A02G250000	1.484	1.9847	3.43E-08	9.60E-07
TraesCS2A02G305000	-1.5571	1.6992	3.46E-08	9.67E-07
TraesCS5B02G169200	2.0496	0.9974	3.51E-08	9.78E-07
TraesCS4A02G103700	1.7083	1.258	3.52E-08	9.82E-07
TraesCS4D02G198000	-1.0171	4.7974	3.58E-08	9.98E-07
TraesCS2D02G116700	-1.3491	2.0198	3.59E-08	9.98E-07
TraesCS3B02G207700	-1.9723	1.3959	3.59E-08	1.00E-06
TraesCS6A02G181400	-1.2877	2.2746	3.61E-08	1.00E-06
TraesCS5A02G216400	-1.0729	3.0973	3.62E-08	1.01E-06
TraesCS4B02G106500	-2.5842	0.3852	3.63E-08	1.01E-06
TraesCS7A02G054100	-2.0466	0.7708	3.64E-08	1.01E-06
TraesCS6D02G009200	-2.6738	0.4399	3.65E-08	1.01E-06
TraesCS1D02G418000	1.2149	2.215	3.67E-08	1.02E-06
TraesCS3A02G132700	1.7823	5.7935	3.74E-08	1.03E-06
TraesCS7B02G118200	-1.2508	3.794	3.75E-08	1.04E-06
TraesCS5D02G299400	1.4908	1.6641	3.81E-08	1.05E-06
TraesCS2D02G099900	-1.606	2.286	3.87E-08	1.06E-06
TraesCS2D02G132500	-1.0895	4.4383	3.91E-08	1.08E-06
TraesCS2A02G100400	-1.3825	2.0572	3.95E-08	1.09E-06
TraesCS3D02G225000	-1.096	3.2872	3.97E-08	1.09E-06
TraesCS2D02G265200	-1.863	1.7045	3.99E-08	1.09E-06
TraesCS1A02G284200	-1.237	2.4923	4.12E-08	1.13E-06
TraesCS4D02G303500	-1.0248	3.3942	4.13E-08	1.13E-06
TraesCS2D02G131800	-4.4822	-0.4414	4.14E-08	1.13E-06

TraesCS6D02G135000	-1.0959	2.7487	4.19E-08	1.14E-06
TraesCS7A02G077000	-1.3167	2.9056	4.19E-08	1.14E-06
TraesCS5A02G121800	-1.5408	1.6503	4.22E-08	1.15E-06
TraesCS2D02G374700	-1.0156	3.6704	4.27E-08	1.16E-06
TraesCS1A02G070900	-1.4148	1.9106	4.28E-08	1.16E-06
TraesCS5D02G368100	-2.0159	1.2472	4.29E-08	1.17E-06
TraesCS3B02G209700	-1.4984	2.2081	4.35E-08	1.18E-06
TraesCS2A02G178100	-1.6231	1.6662	4.35E-08	1.18E-06
TraesCS5D02G427300	-1.5942	2.6498	4.36E-08	1.18E-06
TraesCS2D02G097800	-1.4786	2.0905	4.49E-08	1.21E-06
TraesCS1A02G009300	-3.5077	-0.147	4.49E-08	1.21E-06
TraesCS4B02G066500	-1.3853	2.1451	4.49E-08	1.21E-06
TraesCS6D02G096100	1.0706	3.9588	4.49E-08	1.21E-06
TraesCS5A02G102900	-1.8958	0.9906	4.57E-08	1.23E-06
TraesCS3A02G229900	1.397	3.1351	4.60E-08	1.24E-06
TraesCS7A02G225600	1.2098	2.4592	4.64E-08	1.25E-06
TraesCS3D02G107400	1.3056	3.5773	4.66E-08	1.25E-06
TraesCS3D02G077400	1.1414	2.8062	4.66E-08	1.25E-06
TraesCS1A02G114700	-1.2788	3.2124	4.72E-08	1.27E-06
TraesCS4B02G088100	-1.403	2.4486	4.85E-08	1.30E-06
TraesCS6A02G256900	1.4189	1.8997	4.87E-08	1.31E-06
TraesCS4A02G059400	1.4263	1.8986	5.04E-08	1.35E-06
TraesCS7A02G309600	2.1458	0.7969	5.08E-08	1.36E-06
TraesCS1D02G437100	-1.0218	3.457	5.14E-08	1.37E-06
TraesCS3B02G041700	-1.0375	3.7341	5.15E-08	1.37E-06
TraesCS5D02G343200	3.3555	-0.0122	5.26E-08	1.40E-06
TraesCS7A02G068600	-1.1837	3.6818	5.26E-08	1.40E-06
TraesCS3A02G092700	-1.0918	2.6961	5.32E-08	1.42E-06
TraesCS5D02G014400	-1.1342	2.6933	5.33E-08	1.42E-06
TraesCS6D02G199100	1.4718	4.1655	5.36E-08	1.42E-06
TraesCS3B02G328500	-1.0269	2.9822	5.40E-08	1.43E-06
TraesCS1B02G093900	1.2521	3.47	5.40E-08	1.43E-06
TraesCS5D02G507600	1.6467	1.8307	5.42E-08	1.44E-06
TraesCS3D02G041700	-1.1393	3.6518	5.50E-08	1.46E-06
TraesCS7B02G131600	1.3517	2.1865	5.61E-08	1.49E-06
TraesCS5A02G220100	-1.8786	1.2919	5.64E-08	1.49E-06
TraesCS7D02G357400	1.1889	4.4544	5.82E-08	1.53E-06
TraesCS1D02G292100	1.2434	3.1441	5.85E-08	1.54E-06
TraesCS4A02G137000	-1.1231	3.5536	5.97E-08	1.57E-06
TraesCS2B02G364000	1.0767	3.0971	6.08E-08	1.60E-06
TraesCSU02G077300	1.5761	8.1786	6.15E-08	1.61E-06
TraesCS3B02G131200	2.6829	0.7609	6.21E-08	1.63E-06
TraesCS5B02G080000	-1.0717	4.2211	6.38E-08	1.67E-06
TraesCS6B02G275700	-1.0802	3.2139	6.41E-08	1.68E-06
TraesCS4A02G115200	-1.1617	2.7056	6.43E-08	1.68E-06
TraesCS2D02G103800	-1.0084	3.2999	6.43E-08	1.68E-06
TraesCS7D02G238000	-1.0664	2.9616	6.44E-08	1.68E-06
TraesCS5D02G244300	-1.0074	3.0821	6.45E-08	1.68E-06

TraesCS2D02G029300	2.325	0.497	6.46E-08	1.68E-06
TraesCS5B02G196600	-1.104	2.9375	6.47E-08	1.68E-06
TraesCS5D02G095900	-1.0466	3.8886	6.55E-08	1.70E-06
TraesCSU02G017100	-1.2195	2.3403	6.60E-08	1.71E-06
TraesCS7B02G171200	-1.539	2.0485	6.74E-08	1.75E-06
TraesCS7D02G208600	-6.178	-0.6133	6.79E-08	1.76E-06
TraesCS3D02G171700	1.2892	2.6596	6.94E-08	1.80E-06
TraesCS5B02G128800	-6.1711	-0.6155	6.98E-08	1.80E-06
TraesCS3D02G420000	-1.838	1.0661	6.99E-08	1.81E-06
TraesCS5D02G410800	1.342	2.2138	7.05E-08	1.82E-06
TraesCS6D02G066200	1.1491	3.0372	7.06E-08	1.82E-06
TraesCS1A02G166200	1.0102	4.7219	7.08E-08	1.83E-06
TraesCSU02G137900	-1.0452	3.923	7.11E-08	1.83E-06
TraesCS7D02G346700	-1.7242	1.3556	7.25E-08	1.86E-06
TraesCS6B02G091100	1.2564	2.796	7.33E-08	1.88E-06
TraesCSU02G114400	-1.5431	1.4538	7.41E-08	1.90E-06
TraesCS2D02G088200	-2.4761	0.3089	7.63E-08	1.95E-06
TraesCS4D02G303100	3.0212	0.1918	7.72E-08	1.97E-06
TraesCS2D02G187700	-1.0852	3.0691	7.84E-08	2.00E-06
TraesCS1B02G217200	1.3059	3.1638	7.87E-08	2.01E-06
TraesCS2A02G134500	-1.7086	2.0655	7.93E-08	2.02E-06
TraesCS5D02G234700	-1.6763	1.6771	7.97E-08	2.03E-06
TraesCS1A02G028500	-2.713	0.4864	8.02E-08	2.04E-06
TraesCS1D02G019100	1.0564	2.7355	8.02E-08	2.04E-06
TraesCS5B02G028800	-1.3502	2.2571	8.39E-08	2.13E-06
TraesCS3B02G129800	1.0111	3.6839	8.39E-08	2.13E-06
TraesCS3B02G006100	1.0732	2.9669	8.74E-08	2.21E-06
TraesCS4A02G002600	-1.0515	3.3549	8.85E-08	2.24E-06
TraesCS2B02G029100	-1.2406	2.5438	8.92E-08	2.26E-06
TraesCS1A02G305400	1.7028	3.3539	9.02E-08	2.28E-06
TraesCS3B02G235100	-1.1657	2.5966	9.31E-08	2.34E-06
TraesCS6D02G397500	1.8262	1.5794	9.43E-08	2.37E-06
TraesCS7B02G050500	-1.2186	3.2066	9.48E-08	2.38E-06
TraesCS2D02G094800	-1.5551	2.1302	9.55E-08	2.40E-06
TraesCS5A02G214600	-1.1393	3.4068	9.79E-08	2.45E-06
TraesCS5D02G061100	-1.3066	2.3945	9.83E-08	2.46E-06
TraesCS2B02G199600	-1.0199	3.2912	9.94E-08	2.48E-06
TraesCS4B02G231400	1.2003	2.3346	1.01E-07	2.52E-06
TraesCS5D02G059600	-2.6643	0.3054	1.02E-07	2.53E-06
TraesCS3B02G086900	-1.1907	2.4818	1.02E-07	2.54E-06
TraesCS7A02G223800	-3.4353	-0.2007	1.03E-07	2.56E-06
TraesCS7A02G240600	-1.3043	1.903	1.03E-07	2.57E-06
TraesCS5A02G205300	2.432	0.6632	1.04E-07	2.57E-06
TraesCS1D02G080900	-1.0481	3.9205	1.04E-07	2.57E-06
TraesCS7D02G197500	2.4133	4.0841	1.04E-07	2.58E-06
TraesCS4A02G109700	-1.0178	2.9604	1.05E-07	2.59E-06
TraesCS7D02G408400	1.1642	3.7647	1.05E-07	2.61E-06
TraesCS7A02G122400	-1.5672	1.7018	1.07E-07	2.64E-06

TraesCS1D02G427400	-1.0282	3.0022	1.09E-07	2.69E-06
TraesCS2D02G291800	-1.5709	1.7355	1.11E-07	2.73E-06
TraesCS5D02G441100	-1.9408	1.0282	1.12E-07	2.77E-06
TraesCS5D02G428100	-1.9768	2.4592	1.13E-07	2.77E-06
TraesCSU02G196400	-2.3151	0.7103	1.17E-07	2.88E-06
TraesCS1B02G142100	-1.4878	2.0827	1.18E-07	2.90E-06
TraesCS7A02G087400	-1.2372	2.4299	1.18E-07	2.90E-06
TraesCS1D02G376600	1.3588	2.1301	1.19E-07	2.92E-06
TraesCS4B02G191300	-1.3394	2.0193	1.19E-07	2.92E-06
TraesCS2D02G186700	-1.7077	1.4918	1.20E-07	2.93E-06
TraesCS6A02G226200	1.3932	2.0404	1.20E-07	2.94E-06
TraesCS6A02G123300	1.2413	2.7375	1.20E-07	2.95E-06
TraesCS6A02G179800	-2.0695	0.713	1.22E-07	2.97E-06
TraesCS4D02G275000	-1.8674	0.9712	1.22E-07	2.98E-06
TraesCS1A02G186400	1.3576	2.2055	1.23E-07	3.01E-06
TraesCS4B02G135900	-1.7618	2.0127	1.27E-07	3.09E-06
TraesCS4D02G353900	1.2993	3.4809	1.27E-07	3.10E-06
TraesCS5B02G345800	-1.9665	0.7859	1.28E-07	3.12E-06
TraesCS2B02G115100	1.6772	1.6807	1.29E-07	3.14E-06
TraesCS5A02G103200	1.142	2.9023	1.32E-07	3.22E-06
TraesCS2D02G027700	1.241	2.3036	1.35E-07	3.28E-06
TraesCS1B02G231600	-1.0939	2.7134	1.37E-07	3.33E-06
TraesCS2A02G220400	1.0939	4.2701	1.38E-07	3.35E-06
TraesCS2D02G396000	-1.216	2.26	1.40E-07	3.39E-06
TraesCS1D02G228100	-1.6208	1.9796	1.42E-07	3.42E-06
TraesCS5A02G237500	1.2065	2.7297	1.44E-07	3.48E-06
TraesCS2D02G336900	1.4209	1.7487	1.45E-07	3.49E-06
TraesCS1B02G077500	-1.2249	2.1749	1.46E-07	3.52E-06
TraesCS5A02G235200	1.2778	1.9992	1.46E-07	3.52E-06
TraesCS4A02G228100	-1.7692	2.2635	1.50E-07	3.61E-06
TraesCS6B02G117300	1.5725	6.0266	1.50E-07	3.61E-06
TraesCS4B02G015300	-2.1644	1.3245	1.51E-07	3.62E-06
TraesCS3B02G205100	1.0545	6.6214	1.52E-07	3.64E-06
TraesCS2D02G094500	-1.1314	2.3629	1.55E-07	3.70E-06
TraesCS7D02G192900	-1.2587	2.0034	1.59E-07	3.79E-06
TraesCS1A02G050600	-1.4956	1.9048	1.60E-07	3.82E-06
TraesCS4D02G298500	-1.382	1.6622	1.60E-07	3.82E-06
TraesCS3B02G290200	1.1621	2.2964	1.62E-07	3.85E-06
TraesCS7A02G121700	-1.1689	2.507	1.63E-07	3.88E-06
TraesCS5A02G265800	-2.4469	0.2891	1.63E-07	3.88E-06
TraesCS3A02G072600	-1.1759	2.3347	1.71E-07	4.04E-06
TraesCS1D02G101100	2.1753	0.5771	1.72E-07	4.06E-06
TraesCS6A02G131200	1.3363	1.7881	1.72E-07	4.07E-06
TraesCS7A02G066200	-1.3241	2.65	1.77E-07	4.18E-06
TraesCS3D02G419600	-2.9257	0.3297	1.80E-07	4.24E-06
TraesCS2B02G373300	-1.2886	2.6934	1.84E-07	4.32E-06
TraesCS7A02G131400	-3.0716	0.0734	1.87E-07	4.41E-06
TraesCS3D02G095300	-1.4666	1.7633	1.88E-07	4.42E-06

TraesCS5A02G228900	-1.1148	3.9571	1.89E-07	4.44E-06
TraesCS3A02G231500	-1.4283	2.1068	1.90E-07	4.45E-06
TraesCS6A02G144100	-3.134	-0.1194	1.90E-07	4.45E-06
TraesCS3A02G083500	-1.1073	2.8447	1.90E-07	4.46E-06
TraesCS1B02G033200	-1.187	4.5342	2.03E-07	4.74E-06
TraesCS4A02G182700	-1.1122	2.5687	2.07E-07	4.82E-06
TraesCS3B02G252600	-1.1633	2.5747	2.08E-07	4.83E-06
TraesCS1A02G308500	1.0002	5.4563	2.13E-07	4.94E-06
TraesCS1B02G000400	-2.1855	0.5387	2.14E-07	4.97E-06
TraesCS2B02G341800	-1.1318	2.6038	2.18E-07	5.04E-06
TraesCS1D02G227400	-2.139	0.5087	2.20E-07	5.09E-06
TraesCS3D02G392200	-1.2806	2.1604	2.22E-07	5.13E-06
TraesCS5D02G207400	1.706	1.2159	2.24E-07	5.16E-06
TraesCS7B02G139000	-1.4819	1.5454	2.27E-07	5.23E-06
TraesCSU02G160100	-1.7478	1.0998	2.28E-07	5.25E-06
TraesCS7D02G079600	-1.2333	2.1722	2.28E-07	5.26E-06
TraesCS5B02G200000	1.5071	1.4571	2.32E-07	5.33E-06
TraesCS3B02G099300	-1.2407	2.605	2.33E-07	5.35E-06
TraesCS7D02G101400	-1.5957	1.7544	2.43E-07	5.58E-06
TraesCSU02G113900	1.0328	5.4069	2.50E-07	5.73E-06
TraesCS6B02G022300	-1.6968	1.0633	2.52E-07	5.76E-06
TraesCS4D02G251900	1.2631	4.5908	2.53E-07	5.79E-06
TraesCS4B02G074700	-1.2829	3.0192	2.59E-07	5.92E-06
TraesCS7A02G174500	-2.4758	0.3101	2.66E-07	6.05E-06
TraesCS3D02G411600	-1.0494	3.2147	2.68E-07	6.09E-06
TraesCS7D02G361200	-1.2967	2.6884	2.72E-07	6.18E-06
TraesCS4D02G007200	1.9751	1.6091	2.75E-07	6.24E-06
TraesCS5B02G289100	-1.1211	2.4665	2.77E-07	6.28E-06
TraesCS5D02G420800	-1.1414	2.5799	2.82E-07	6.39E-06
TraesCS1B02G282000	-2.4326	0.9706	2.90E-07	6.55E-06
TraesCS2A02G183900	1.4323	1.6449	2.90E-07	6.55E-06
TraesCS3B02G200000	1.263	2.5311	2.99E-07	6.75E-06
TraesCS2B02G372500	-1.0932	3.9318	3.01E-07	6.78E-06
TraesCS5A02G024100	1.105	2.5172	3.05E-07	6.86E-06
TraesCS3B02G312300	-1.0685	2.7697	3.12E-07	7.01E-06
TraesCS1B02G271300	1.1128	2.5995	3.16E-07	7.08E-06
TraesCS2B02G334000	-1.099	3.0666	3.18E-07	7.12E-06
TraesCS5D02G328600	-1.0126	2.6705	3.18E-07	7.12E-06
TraesCS6D02G139900	-1.7009	1.1232	3.20E-07	7.15E-06
TraesCS2D02G157900	1.109	2.8062	3.23E-07	7.22E-06
TraesCS1A02G220300	1.123	2.9763	3.24E-07	7.23E-06
TraesCS5D02G048100	-1.3201	2.4058	3.25E-07	7.25E-06
TraesCS2A02G275600	-1.2134	2.2367	3.29E-07	7.32E-06
TraesCS5D02G461600	1.7542	1.4178	3.31E-07	7.37E-06
TraesCS1B02G282600	1.2715	1.8673	3.32E-07	7.38E-06
TraesCS3D02G324700	1.342	1.6414	3.39E-07	7.52E-06
TraesCS3A02G050500	2.6434	0.3897	3.43E-07	7.61E-06
TraesCS1D02G240600	-1.7157	1.2968	3.43E-07	7.61E-06

TraesCS5B02G286200	-2.3014	0.3093	3.51E-07	7.76E-06
TraesCS4D02G190300	-1.1829	2.2375	3.51E-07	7.76E-06
TraesCS5A02G304000	2.8397	0.2455	3.52E-07	7.79E-06
TraesCS5A02G304100	2.8397	0.2455	3.53E-07	7.79E-06
TraesCS7A02G206000	-1.5586	1.7427	3.55E-07	7.83E-06
TraesCS7A02G203000	-1.9998	0.9494	3.58E-07	7.89E-06
TraesCS7D02G314200	1.2088	3.1363	3.58E-07	7.89E-06
TraesCS5B02G252600	1.0157	4.7086	3.59E-07	7.90E-06
TraesCS5B02G116200	-1.8701	1.7995	3.60E-07	7.93E-06
TraesCS7A02G191700	-1.3794	2.2763	3.67E-07	8.06E-06
TraesCS7B02G205400	-1.1634	2.5953	3.72E-07	8.16E-06
TraesCS2D02G370000	1.831	1.8372	3.84E-07	8.41E-06
TraesCS1D02G249000	-1.3835	1.7973	3.86E-07	8.45E-06
TraesCS5D02G044500	-2.7324	0.0303	3.89E-07	8.51E-06
TraesCS7A02G162300	2.4675	0.2518	3.98E-07	8.69E-06
TraesCS4D02G032200	-1.1073	2.6407	4.01E-07	8.76E-06
TraesCS3D02G004600	1.8882	0.9452	4.02E-07	8.77E-06
TraesCS5D02G318800	1.2268	2.2005	4.06E-07	8.86E-06
TraesCS2D02G338100	-1.022	3.2113	4.09E-07	8.90E-06
TraesCS7A02G188300	1.671	1.2364	4.17E-07	9.08E-06
TraesCS7D02G179500	1.851	0.9133	4.31E-07	9.36E-06
TraesCS7A02G073000	2.6002	2.4426	4.35E-07	9.43E-06
TraesCS3D02G279300	-1.0181	3.5265	4.37E-07	9.47E-06
TraesCS3D02G025000	-1.051	3.3998	4.45E-07	9.64E-06
TraesCS2D02G174500	-1.0601	2.5652	4.48E-07	9.68E-06
TraesCS6D02G298100	-2.5001	0.1885	4.48E-07	9.70E-06
TraesCS1D02G155400	1.5384	4.7637	4.57E-07	9.86E-06
TraesCS7D02G115200	-2.2552	0.385	4.57E-07	9.87E-06
TraesCS5B02G316900	-1.1607	2.1871	4.59E-07	9.91E-06
TraesCS4D02G317200	-1.6354	1.6854	4.68E-07	1.01E-05
TraesCS5A02G119700	-1.6755	1.1009	4.68E-07	1.01E-05
TraesCS4A02G061600	-1.1271	2.79	4.70E-07	1.01E-05
TraesCS5D02G292600	-1.2048	2.6122	4.77E-07	1.02E-05
TraesCS3A02G026400	1.3723	1.6643	4.83E-07	1.04E-05
TraesCS7D02G019200	-1.2723	1.9904	4.83E-07	1.04E-05
TraesCS4D02G240300	-1.0242	2.8822	4.91E-07	1.05E-05
TraesCS1A02G185800	-1.7448	0.9929	4.94E-07	1.06E-05
TraesCS5B02G346000	-1.6443	1.1758	4.97E-07	1.07E-05
TraesCS5B02G170300	1.278	2.0744	4.98E-07	1.07E-05
TraesCS3A02G263000	1.0276	2.6164	5.00E-07	1.07E-05
TraesCS7D02G215700	1.8367	0.9058	5.18E-07	1.11E-05
TraesCS2B02G016100	-1.4851	1.6596	5.21E-07	1.11E-05
TraesCS5D02G227700	1.5659	1.2784	5.23E-07	1.11E-05
TraesCS7A02G273300	-1.1415	2.6627	5.31E-07	1.13E-05
TraesCS4A02G067900	-1.171	2.1249	5.38E-07	1.14E-05
TraesCS7D02G361300	-1.2075	2.0855	5.45E-07	1.16E-05
TraesCS4D02G071200	-2.3074	0.7865	5.46E-07	1.16E-05
TraesCS4B02G021800	-1.4339	3.0832	5.61E-07	1.19E-05

TraesCS7D02G261900	-2.5558	0.0779	5.61E-07	1.19E-05
TraesCS1D02G218900	1.5745	1.1662	5.92E-07	1.25E-05
TraesCS5D02G410700	1.1897	2.3229	6.08E-07	1.28E-05
TraesCS1D02G389100	1.1535	2.9355	6.12E-07	1.29E-05
TraesCS3A02G256200	1.1159	2.6854	6.17E-07	1.30E-05
TraesCS4B02G186300	1.9338	1.0383	6.25E-07	1.31E-05
TraesCS7D02G003600	-1.4623	2.3531	6.30E-07	1.32E-05
TraesCS3B02G059300	2.3242	0.4919	6.35E-07	1.33E-05
TraesCS1D02G249700	1.0104	3.8579	6.42E-07	1.35E-05
TraesCS6B02G178300	-1.8517	1.124	6.49E-07	1.36E-05
TraesCS2D02G351000	-1.2119	3.915	6.60E-07	1.38E-05
TraesCS1D02G387600	-1.1397	2.4004	6.66E-07	1.39E-05
TraesCS3A02G072900	-1.0714	2.6924	6.74E-07	1.41E-05
TraesCS5B02G218600	1.7953	0.8149	6.76E-07	1.41E-05
TraesCS3D02G223400	1.3008	2.0866	6.79E-07	1.41E-05
TraesCS4D02G115200	3.3414	-0.312	6.81E-07	1.42E-05
TraesCS5A02G094200	-1.059	2.6951	6.83E-07	1.42E-05
TraesCS5A02G214700	-1.211	2.859	6.84E-07	1.42E-05
TraesCS2A02G287100	-1.2621	1.9163	6.86E-07	1.42E-05
TraesCS4D02G016200	-1.5413	2.5922	6.87E-07	1.43E-05
TraesCS4D02G316600	1.7306	1.5832	6.90E-07	1.43E-05
TraesCS3B02G095900	-1.52	2.0917	6.95E-07	1.44E-05
TraesCS1B02G119600	1.2228	2.2208	7.11E-07	1.47E-05
TraesCS1D02G371800	-1.1699	2.9533	7.25E-07	1.50E-05
TraesCS1B02G188400	-1.2414	2.2264	7.36E-07	1.52E-05
TraesCS4A02G215100	-1.6818	1.4747	7.38E-07	1.52E-05
TraesCS6B02G122500	-1.3666	1.4375	7.62E-07	1.56E-05
TraesCSU02G014400	1.0405	2.4042	7.80E-07	1.60E-05
TraesCS5B02G153100	2.8723	-0.1156	7.83E-07	1.60E-05
TraesCS6B02G077800	1.1646	2.8628	7.91E-07	1.62E-05
TraesCS7B02G136000	-1.1093	2.8156	7.96E-07	1.63E-05
TraesCS6D02G387700	2.3143	0.2719	8.12E-07	1.66E-05
TraesCS3B02G286500	-1.0141	3.3446	8.34E-07	1.70E-05
TraesCS3B02G213800	-1.2628	1.9074	8.36E-07	1.70E-05
TraesCS5B02G195400	-1.3386	2.0191	8.38E-07	1.70E-05
TraesCS4D02G112000	1.4267	3.4834	8.41E-07	1.71E-05
TraesCS5A02G203400	-1.6144	1.75	8.42E-07	1.71E-05
TraesCS1B02G184300	-1.5268	1.1748	8.43E-07	1.71E-05
TraesCS5D02G399300	1.6085	1.2347	8.46E-07	1.72E-05
TraesCS5D02G158700	3.7507	-0.4101	8.68E-07	1.76E-05
TraesCS4B02G260500	-1.0627	2.7033	8.68E-07	1.76E-05
TraesCS4A02G053100	1.0513	3.2741	8.70E-07	1.76E-05
TraesCS1A02G038100	-1.3049	4.0459	8.70E-07	1.76E-05
TraesCS6B02G091300	2.3122	0.6643	8.72E-07	1.76E-05
TraesCS3D02G040700	1.6005	1.2182	9.07E-07	1.82E-05
TraesCS2D02G032700	-1.1243	3.1092	9.13E-07	1.83E-05
TraesCS5A02G271100	-1.0273	2.671	9.16E-07	1.84E-05
TraesCS7A02G061000	1.7265	0.8855	9.51E-07	1.90E-05

TraesCS5D02G125900	-1.6566	1.7403	9.58E-07	1.92E-05
TraesCS6D02G362900	-1.0256	2.3982	9.74E-07	1.95E-05
TraesCS1D02G090800	1.0511	2.7963	9.88E-07	1.97E-05
TraesCS5D02G278100	1.8358	0.7153	9.92E-07	1.98E-05
TraesCS6A02G243600	1.1749	5.193	1.02E-06	2.02E-05
TraesCS4D02G103900	-1.2701	1.9815	1.02E-06	2.02E-05
TraesCS7A02G317100	-1.1491	2.2977	1.02E-06	2.03E-05
TraesCS2B02G284500	-1.1381	2.5747	1.03E-06	2.04E-05
TraesCS7A02G158200	1.0068	3.5456	1.04E-06	2.07E-05
TraesCS2A02G258200	1.0969	2.2948	1.04E-06	2.07E-05
TraesCS4A02G029500	-1.4255	1.6685	1.04E-06	2.07E-05
TraesCS2B02G372600	3.3017	-0.3411	1.06E-06	2.10E-05
TraesCS4D02G320900	-1.2393	2.3727	1.07E-06	2.12E-05
TraesCS4D02G356900	-1.2957	1.7352	1.08E-06	2.15E-05
TraesCS4B02G227000	-1.141	2.3175	1.09E-06	2.15E-05
TraesCS2D02G070500	-1.0541	2.6756	1.09E-06	2.15E-05
TraesCS5A02G250900	-2.7645	-0.1462	1.09E-06	2.15E-05
TraesCS4D02G238600	1.6689	1.3985	1.09E-06	2.16E-05
TraesCS5A02G003700	-1.1163	3.0378	1.10E-06	2.18E-05
TraesCS4D02G303200	2.1481	4.2006	1.11E-06	2.18E-05
TraesCS6B02G125800	2.867	-0.1191	1.13E-06	2.22E-05
TraesCS4B02G072400	-1.9978	0.6691	1.18E-06	2.31E-05
TraesCS3D02G134200	-1.5133	1.3683	1.18E-06	2.31E-05
TraesCS6B02G275800	-2.2502	0.2694	1.19E-06	2.32E-05
TraesCS1D02G381100	-1.1083	2.4139	1.21E-06	2.37E-05
TraesCS6D02G213100	2.1453	0.3686	1.22E-06	2.37E-05
TraesCS4D02G194700	1.3879	1.5347	1.23E-06	2.39E-05
TraesCS3D02G346300	2.0521	0.493	1.23E-06	2.39E-05
TraesCS5A02G012300	-1.3524	1.4901	1.23E-06	2.40E-05
TraesCS5D02G447800	-1.2182	2.2579	1.24E-06	2.41E-05
TraesCSU02G249900	3.3704	-0.2874	1.25E-06	2.43E-05
TraesCS1D02G250000	1.1374	1.9836	1.26E-06	2.44E-05
TraesCS5D02G232900	-1.0211	3.0276	1.26E-06	2.44E-05
TraesCS1D02G267500	-1.0439	2.3241	1.32E-06	2.57E-05
TraesCS3D02G253200	2.5914	0.0581	1.33E-06	2.58E-05
TraesCS7A02G191000	1.2654	2.3753	1.34E-06	2.59E-05
TraesCS2D02G186800	-1.6298	2.0536	1.34E-06	2.59E-05
TraesCS3B02G300400	-1.0367	2.8337	1.35E-06	2.61E-05
TraesCS1A02G101200	-1.0928	2.7596	1.36E-06	2.63E-05
TraesCSU02G204900	-1.0019	3.7957	1.36E-06	2.63E-05
TraesCS1D02G333800	-1.2172	2.5344	1.37E-06	2.64E-05
TraesCS7B02G174100	-1.3372	1.9468	1.41E-06	2.71E-05
TraesCS2D02G076300	-2.3365	0.2098	1.42E-06	2.73E-05
TraesCS3B02G153100	1.18	2.4518	1.43E-06	2.75E-05
TraesCS6A02G234400	-1.073	2.2748	1.44E-06	2.77E-05
TraesCS5D02G244000	1.0021	2.6012	1.46E-06	2.80E-05
TraesCS7A02G132900	-3.0384	0.049	1.47E-06	2.82E-05
TraesCS3B02G244200	-1.7579	1.0047	1.47E-06	2.82E-05

TraesCS6B02G018700	-1.0771	3.8113	1.52E-06	2.90E-05
TraesCS2D02G145100	-1.0098	2.4534	1.53E-06	2.92E-05
TraesCS1D02G036500	1.1085	3.1704	1.54E-06	2.94E-05
TraesCS7B02G168500	-1.2817	1.7748	1.55E-06	2.96E-05
TraesCS1B02G193600	-1.7364	0.8824	1.56E-06	2.96E-05
TraesCS2D02G372500	-1.0096	2.6358	1.57E-06	2.99E-05
TraesCS7D02G120900	-1.2403	1.9915	1.57E-06	2.99E-05
TraesCS7B02G072700	-1.3662	1.6928	1.58E-06	3.00E-05
TraesCS5D02G172100	-1.4101	1.4036	1.59E-06	3.03E-05
TraesCS7D02G354000	-2.1996	0.3494	1.60E-06	3.04E-05
TraesCS7B02G259000	1.5813	2.5181	1.61E-06	3.06E-05
TraesCS5A02G163400	-1.0615	4.1963	1.62E-06	3.07E-05
TraesCS5D02G115100	1.0959	3.1754	1.65E-06	3.12E-05
TraesCS2D02G412800	-1.621	1.065	1.66E-06	3.13E-05
TraesCS7D02G192000	1.4872	3.3326	1.66E-06	3.13E-05
TraesCSU02G118100	1.2142	3.577	1.75E-06	3.29E-05
TraesCS7D02G364500	-1.6051	1.0509	1.75E-06	3.29E-05
TraesCS3B02G308500	-1.452	1.2886	1.75E-06	3.30E-05
TraesCS1D02G310300	1.1806	5.9426	1.77E-06	3.31E-05
TraesCS5A02G128700	-1.3726	1.3759	1.77E-06	3.32E-05
TraesCS5A02G312000	1.3864	4.1366	1.77E-06	3.33E-05
TraesCS5A02G248100	1.0345	3.3325	1.80E-06	3.36E-05
TraesCS6A02G236900	1.5885	0.9472	1.80E-06	3.37E-05
TraesCS7D02G225800	-1.6707	1.0521	1.82E-06	3.39E-05
TraesCS1A02G028900	-1.5381	1.142	1.82E-06	3.39E-05
TraesCS3D02G332400	1.0353	2.318	1.85E-06	3.44E-05
TraesCS4D02G111100	-1.4518	1.2082	1.85E-06	3.45E-05
TraesCS4A02G064900	-1.2284	1.6361	1.87E-06	3.49E-05
TraesCS6A02G216500	1.2374	3.5846	1.89E-06	3.51E-05
TraesCS3B02G251300	-1.4835	1.4821	1.89E-06	3.52E-05
TraesCS5A02G134700	-1.0555	2.6442	1.92E-06	3.57E-05
TraesCS1B02G182300	-1.3325	1.4459	1.94E-06	3.59E-05
TraesCS1A02G088600	1.2466	1.6575	1.98E-06	3.66E-05
TraesCS7A02G074800	-4.236	-0.6124	1.99E-06	3.69E-05
TraesCS4D02G021000	1.8083	1.5273	2.00E-06	3.70E-05
TraesCS4B02G086100	2.2499	0.5435	2.01E-06	3.71E-05
TraesCS6D02G335100	1.3765	2.1844	2.02E-06	3.72E-05
TraesCS1A02G056400	-3.0649	-0.1691	2.03E-06	3.73E-05
TraesCS7A02G040900	-1.313	3.098	2.03E-06	3.75E-05
TraesCS3B02G150500	1.9896	0.9021	2.04E-06	3.76E-05
TraesCS2B02G177600	1.1713	2.6112	2.07E-06	3.81E-05
TraesCS4B02G244600	-1.4032	1.6343	2.10E-06	3.87E-05
TraesCS5B02G054100	-1.7427	0.8279	2.13E-06	3.92E-05
TraesCS4B02G191600	-1.1439	1.9315	2.14E-06	3.94E-05
TraesCSU02G271300	-2.6573	0.4556	2.16E-06	3.97E-05
TraesCS2B02G184300	2.3373	0.4996	2.18E-06	3.99E-05
TraesCS4D02G060400	1.779	0.7412	2.18E-06	3.99E-05
TraesCS1B02G194600	2.6134	0.074	2.19E-06	4.00E-05

TraesCS3D02G097900	-1.0517	2.9209	2.19E-06	4.00E-05
TraesCS3B02G281700	-1.3034	2.1897	2.22E-06	4.05E-05
TraesCS6D02G235200	-1.6859	0.7849	2.22E-06	4.05E-05
TraesCS3A02G222400	-1.0101	3.2312	2.22E-06	4.06E-05
TraesCS1B02G195100	-1.0549	2.9813	2.23E-06	4.07E-05
TraesCS3A02G165700	1.1322	2.7985	2.23E-06	4.07E-05
TraesCS4B02G194500	-1.0248	2.7244	2.24E-06	4.07E-05
TraesCSU02G145400	-1.0261	2.8988	2.27E-06	4.13E-05
TraesCS4B02G162700	-1.2542	1.7762	2.32E-06	4.21E-05
TraesCS5D02G156600	-1.1869	1.8256	2.37E-06	4.30E-05
TraesCS1D02G130900	-1.382	1.5707	2.41E-06	4.36E-05
TraesCS7B02G274200	-1.2711	1.9715	2.42E-06	4.39E-05
TraesCS2A02G294600	1.1314	2.7639	2.45E-06	4.43E-05
TraesCS4D02G212300	1.8005	0.9969	2.45E-06	4.43E-05
TraesCS4B02G251400	1.3113	1.9922	2.47E-06	4.47E-05
TraesCS5D02G168400	-1.0972	3.0329	2.48E-06	4.48E-05
TraesCS5B02G312900	2.0054	0.6988	2.50E-06	4.50E-05
TraesCS1B02G181300	-1.609	0.9052	2.52E-06	4.54E-05
TraesCS1B02G290400	-1.0885	2.4703	2.53E-06	4.56E-05
TraesCS5B02G335800	-1.1471	1.8244	2.57E-06	4.63E-05
TraesCS5D02G216200	1.0244	2.567	2.61E-06	4.68E-05
TraesCS5A02G070600	1.5478	1.5861	2.62E-06	4.70E-05
TraesCS4D02G103100	1.8028	0.8827	2.67E-06	4.79E-05
TraesCS4B02G237000	-1.2469	1.6786	2.70E-06	4.82E-05
TraesCS1D02G370700	-1.3669	1.3351	2.74E-06	4.89E-05
TraesCS1B02G113600	3.2436	-0.3778	2.76E-06	4.93E-05
TraesCS4A02G034500	1.1525	2.0652	2.82E-06	5.02E-05
TraesCS5D02G323200	1.236	3.0773	2.82E-06	5.03E-05
TraesCS5D02G199900	-1.7659	1.1663	2.85E-06	5.07E-05
TraesCS1B02G143500	-1.3738	1.7018	2.89E-06	5.13E-05
TraesCS6D02G319500	-1.7404	0.6977	2.90E-06	5.14E-05
TraesCS2B02G031700	-1.2619	2.1824	2.91E-06	5.16E-05
ENSRNA050015984	-1.1059	4.0405	2.94E-06	5.20E-05
TraesCS2D02G006400	-1.264	1.7139	3.01E-06	5.32E-05
TraesCS2B02G357500	-1.0452	2.8497	3.06E-06	5.39E-05
TraesCSU02G171500	3.252	-0.3736	3.09E-06	5.45E-05
TraesCS6A02G273600	-1.5594	1.9331	3.13E-06	5.52E-05
TraesCS4D02G329200	-1.6406	0.812	3.14E-06	5.52E-05
TraesCS4D02G195100	-1.0133	3.247	3.15E-06	5.54E-05
TraesCS3D02G180800	1.1122	3.6923	3.17E-06	5.57E-05
TraesCS3D02G166000	-1.3822	1.8255	3.18E-06	5.58E-05
TraesCS5D02G184000	1.2525	3.0732	3.18E-06	5.58E-05
TraesCS6B02G133900	1.815	0.7041	3.22E-06	5.66E-05
TraesCS1D02G065300	-2.6832	-0.1999	3.28E-06	5.74E-05
TraesCS1B02G119700	1.0075	2.6563	3.30E-06	5.78E-05
TraesCS5A02G147700	1.8615	3.05	3.40E-06	5.92E-05
TraesCS5A02G220400	-1.0623	3.2606	3.42E-06	5.96E-05
TraesCS2B02G233700	-1.7133	0.869	3.42E-06	5.97E-05

TraesCS7B02G003800	-2.402	0.1225	3.49E-06	6.08E-05
TraesCS2B02G206100	-1.4484	1.2042	3.50E-06	6.09E-05
TraesCS5B02G264800	-3.1357	-0.4093	3.59E-06	6.24E-05
TraesCS4B02G219500	1.8621	2.4436	3.60E-06	6.25E-05
TraesCS2A02G143400	1.6196	1.4027	3.66E-06	6.35E-05
TraesCS6D02G009600	-1.3128	1.6611	3.69E-06	6.40E-05
TraesCS1A02G059100	-1.2008	1.8504	3.71E-06	6.44E-05
TraesCS3D02G128300	1.0995	2.9736	3.72E-06	6.44E-05
TraesCS3B02G242300	-1.4005	1.8736	3.77E-06	6.53E-05
TraesCS5A02G245300	-1.0383	2.5996	3.79E-06	6.56E-05
TraesCS5A02G112000	1.6993	0.8645	3.83E-06	6.61E-05
TraesCS1D02G412300	-1.1182	2.3222	3.83E-06	6.61E-05
TraesCS4B02G261300	-1.5106	1.4031	3.87E-06	6.67E-05
TraesCS2D02G396800	1.104	3.534	3.89E-06	6.69E-05
TraesCS7B02G106900	1.0106	2.7129	3.92E-06	6.74E-05
TraesCS1D02G230800	-1.2569	1.5182	3.95E-06	6.78E-05
TraesCS3A02G255500	-1.2126	2.4787	3.96E-06	6.80E-05
TraesCS6D02G306800	-1.4511	1.0405	3.98E-06	6.84E-05
TraesCS5B02G276700	1.0415	2.6197	4.07E-06	6.98E-05
TraesCS1D02G240300	1.1576	1.7914	4.08E-06	6.98E-05
TraesCS3D02G319100	-1.1002	2.2151	4.15E-06	7.10E-05
TraesCS7B02G032300	-1.7778	1.1357	4.15E-06	7.10E-05
TraesCS5D02G203400	1.1612	2.8524	4.19E-06	7.15E-05
TraesCS6D02G012600	-2.79	-0.1198	4.29E-06	7.30E-05
TraesCS7B02G270400	1.8427	0.7273	4.30E-06	7.32E-05
TraesCS1D02G428300	2.5565	2.5234	4.38E-06	7.44E-05
TraesCS1A02G335300	-1.0793	2.3246	4.45E-06	7.56E-05
TraesCS6D02G006500	-1.1954	1.6704	4.48E-06	7.61E-05
TraesCS3A02G131900	-1.6627	1.2111	4.53E-06	7.68E-05
TraesCS2A02G027300	2.4929	0.1448	4.53E-06	7.68E-05
TraesCS4B02G242300	-1.1914	1.6913	4.59E-06	7.77E-05
TraesCS7D02G200600	1.1977	5.6298	4.62E-06	7.81E-05
TraesCS6D02G338700	-1.0866	2.0613	4.63E-06	7.83E-05
TraesCS6D02G250600	-1.1379	2.5508	4.63E-06	7.83E-05
TraesCS3B02G210400	-1.8823	0.5725	4.65E-06	7.85E-05
TraesCS5D02G173900	1.2974	1.5008	4.67E-06	7.89E-05
TraesCS7B02G003900	-1.6687	0.7136	4.69E-06	7.91E-05
TraesCS6D02G085100	1.7295	0.769	4.72E-06	7.95E-05
TraesCS5D02G217700	-1.103	2.3108	4.76E-06	8.01E-05
TraesCS7D02G229300	-1.0192	2.5653	4.82E-06	8.10E-05
TraesCS1B02G075600	-1.2597	1.8549	4.90E-06	8.22E-05
TraesCS1D02G053000	-1.0295	2.8932	4.90E-06	8.22E-05
TraesCS7D02G055500	1.0105	3.8427	4.93E-06	8.27E-05
TraesCS1D02G061500	-1.6023	0.9571	4.94E-06	8.28E-05
TraesCS1A02G289200	1.9024	0.5479	4.98E-06	8.34E-05
TraesCS2B02G205300	-2.1906	0.231	5.07E-06	8.48E-05
TraesCS6D02G033300	-1.9899	0.3078	5.09E-06	8.51E-05
TraesCS6D02G371000	1.6611	2.5341	5.09E-06	8.51E-05

TraesCS7B02G090500	-1.3837	1.7047	5.09E-06	8.51E-05
TraesCS7D02G220500	-1.4975	2.0532	5.13E-06	8.56E-05
ENSRNA050018675	-1.8323	1.2624	5.21E-06	8.68E-05
TraesCS5D02G452700	-1.1506	2.4049	5.24E-06	8.74E-05
TraesCS3D02G305300	1.2342	1.82	5.26E-06	8.76E-05
TraesCS4B02G035700	-1.554	0.9707	5.27E-06	8.78E-05
TraesCS5A02G107300	-1.0477	2.1172	5.28E-06	8.79E-05
TraesCS5D02G311200	1.0337	5.1389	5.32E-06	8.83E-05
TraesCS5D02G204200	-2.2182	0.8286	5.32E-06	8.84E-05
TraesCS2D02G281000	-1.379	1.2379	5.37E-06	8.91E-05
TraesCS4D02G004100	-1.8648	0.4905	5.39E-06	8.94E-05
TraesCS5D02G129400	1.677	0.7334	5.41E-06	8.97E-05
TraesCS6B02G297500	2.5862	-0.1164	5.43E-06	9.00E-05
TraesCS3A02G093600	-1.5246	1.5024	5.43E-06	9.00E-05
TraesCS6D02G298200	-1.5868	1.9157	5.48E-06	9.08E-05
TraesCS7D02G300500	-1.0127	2.3966	5.49E-06	9.09E-05
TraesCS3A02G140600	1.6616	2.7534	5.50E-06	9.10E-05
TraesCS3D02G371700	1.858	0.7942	5.58E-06	9.21E-05
TraesCS4D02G060500	1.6616	0.8385	5.60E-06	9.25E-05
TraesCS7B02G018500	-1.2215	2.206	5.65E-06	9.32E-05
TraesCS7D02G322700	-1.3919	1.087	5.70E-06	9.38E-05
TraesCS7D02G051000	-1.5149	1.3139	5.78E-06	9.50E-05
TraesCS1D02G257800	2.1903	0.1844	5.80E-06	9.53E-05
TraesCS4B02G033100	-1.1011	2.9044	5.98E-06	9.81E-05
TraesCS7D02G399700	-2.266	0.5051	6.00E-06	9.84E-05
TraesCS2A02G007900	1.1588	4.3531	6.06E-06	9.92E-05
TraesCS2B02G278100	-2.196	0.4594	6.14E-06	1.00E-04
TraesCS6D02G121700	-1.3743	1.501	6.23E-06	1.02E-04
TraesCS5B02G279700	1.7835	0.8695	6.30E-06	1.03E-04
TraesCS6D02G313000	1.1136	3.356	6.36E-06	1.03E-04
TraesCS7D02G078900	-1.4953	1.6298	6.42E-06	1.04E-04
TraesCS2A02G005300	-1.3391	1.6121	6.59E-06	1.07E-04
TraesCS6B02G277900	-1.364	1.9296	6.59E-06	1.07E-04
TraesCS7A02G113800	-2.2975	0.1848	6.59E-06	1.07E-04
TraesCS7A02G234200	-1.5639	1.4472	6.60E-06	1.07E-04
TraesCS3D02G323900	-1.1385	2.006	6.60E-06	1.07E-04
TraesCS2D02G394600	2.3409	0.0331	6.71E-06	1.08E-04
TraesCS1D02G031500	1.4786	2.7021	6.71E-06	1.08E-04
TraesCS5D02G305800	1.3777	1.4285	6.74E-06	1.09E-04
TraesCS2B02G293300	-1.0868	2.236	6.75E-06	1.09E-04
TraesCS3B02G108700	-1.2409	1.9253	6.90E-06	1.11E-04
TraesCS7A02G089400	-1.4567	1.8069	6.90E-06	1.11E-04
TraesCS1A02G257200	1.2302	1.9859	6.97E-06	1.12E-04
TraesCS1D02G012300	-1.5743	1.565	7.03E-06	1.13E-04
TraesCS5B02G004300	-1.0732	2.1746	7.05E-06	1.13E-04
TraesCS5A02G063900	1.0721	2.7236	7.11E-06	1.14E-04
TraesCS3A02G113500	1.1472	3.7275	7.20E-06	1.16E-04
TraesCS7D02G353900	-1.2142	1.7722	7.29E-06	1.17E-04

TraesCS6D02G030200	1.1338	2.2728	7.30E-06	1.17E-04
TraesCS1B02G048300	-1.7562	0.88	7.39E-06	1.18E-04
TraesCS4B02G022900	-1.8679	0.8413	7.54E-06	1.20E-04
TraesCS1A02G331300	-1.5785	1.443	7.62E-06	1.22E-04
TraesCS2A02G257600	1.566	0.8842	7.73E-06	1.23E-04
TraesCS5B02G246300	-1.2371	1.8548	7.74E-06	1.23E-04
TraesCS7A02G244600	-1.0368	3.1722	7.80E-06	1.24E-04
TraesCS6B02G291200	-2.0573	0.248	7.83E-06	1.25E-04
TraesCS5A02G237600	-1.141	1.8837	7.87E-06	1.25E-04
TraesCS3A02G038000	-2.5163	-0.1174	7.87E-06	1.25E-04
TraesCS2A02G001500	-1.5537	0.9172	7.98E-06	1.27E-04
TraesCS3B02G006300	1.1831	5.1144	8.00E-06	1.27E-04
TraesCS2A02G212900	-1.1203	2.5291	8.03E-06	1.27E-04
TraesCS2A02G118300	-1.3775	1.2689	8.06E-06	1.28E-04
TraesCS6A02G238300	2.2617	3.3098	8.10E-06	1.28E-04
TraesCS3D02G174300	-1.0995	2.2169	8.12E-06	1.29E-04
TraesCS2D02G388500	1.4753	1.2959	8.13E-06	1.29E-04
TraesCS4D02G159600	1.0304	2.3125	8.25E-06	1.30E-04
TraesCSU02G145700	1.9761	0.3497	8.57E-06	1.35E-04
TraesCS6A02G168600	1.0476	2.0576	8.67E-06	1.36E-04
TraesCS5A02G241000	-1.811	0.6097	8.67E-06	1.36E-04
TraesCS1D02G100900	1.1051	1.906	8.74E-06	1.37E-04
ENSRNA050020224	2.9323	-0.3111	8.97E-06	1.40E-04
TraesCS1B02G230200	1.7622	0.7941	9.00E-06	1.41E-04
TraesCS4A02G189700	-1.2317	1.6788	9.05E-06	1.41E-04
TraesCS6D02G242100	1.7426	0.6547	9.14E-06	1.43E-04
TraesCS5B02G013800	1.4323	1.4783	9.20E-06	1.43E-04
TraesCS4D02G359900	-1.7382	0.8844	9.30E-06	1.45E-04
TraesCS5A02G290100	-1.0324	2.2828	9.39E-06	1.46E-04
TraesCS7A02G313900	-1.2989	1.4592	9.49E-06	1.48E-04
TraesCS5B02G242800	-1.0384	2.0149	9.54E-06	1.48E-04
TraesCS4A02G103300	1.2616	5.7237	9.69E-06	1.51E-04
TraesCS2A02G206500	-1.3762	1.5597	9.75E-06	1.51E-04
TraesCS5D02G342100	1.4409	1.2404	9.75E-06	1.51E-04
TraesCS7A02G261700	-1.374	1.3325	9.82E-06	1.52E-04
TraesCS1A02G268900	1.7938	0.6224	9.84E-06	1.52E-04
TraesCS4D02G104000	-1.019	3.0435	9.85E-06	1.52E-04
TraesCS1A02G028600	-1.627	1.0733	9.88E-06	1.52E-04
TraesCS6A02G297500	1.5715	1.1257	1.00E-05	1.54E-04
TraesCS7D02G013200	-1.1886	1.6601	1.01E-05	1.56E-04
TraesCS1B02G195600	-2.1292	0.2884	1.02E-05	1.57E-04
TraesCS6B02G288700	1.5227	0.9996	1.05E-05	1.60E-04
TraesCS7A02G106000	1.3685	1.4545	1.05E-05	1.60E-04
TraesCS2B02G125600	-1.8873	1.847	1.07E-05	1.63E-04
TraesCS2A02G281100	1.3437	1.2071	1.10E-05	1.68E-04
TraesCS5B02G271400	-1.1907	2.1716	1.10E-05	1.68E-04
TraesCS2D02G191800	1.0801	2.5414	1.13E-05	1.72E-04
TraesCS5A02G010900	1.9049	0.8954	1.14E-05	1.73E-04

TraesCS7B02G262400	1.2537	1.4115	1.14E-05	1.74E-04
ENSRNA050015978	-1.7031	2.3205	1.15E-05	1.74E-04
TraesCS1B02G292200	-1.1354	1.7461	1.16E-05	1.76E-04
TraesCS6B02G210300	-1.4029	1.1323	1.17E-05	1.77E-04
TraesCS3B02G006400	1.9284	0.9005	1.18E-05	1.78E-04
TraesCS4D02G197800	-1.7613	0.718	1.21E-05	1.83E-04
TraesCS6A02G235800	-1.4461	1.2501	1.21E-05	1.83E-04
TraesCS4A02G098600	1.0644	2.5375	1.21E-05	1.83E-04
TraesCS4A02G196800	-1.7334	0.6226	1.23E-05	1.86E-04
TraesCS4B02G018400	-1.2742	1.6645	1.27E-05	1.90E-04
TraesCS4B02G220000	1.0115	2.0684	1.27E-05	1.90E-04
TraesCS7D02G044500	1.793	0.4757	1.28E-05	1.92E-04
TraesCS7B02G251200	-1.5051	0.9784	1.29E-05	1.92E-04
TraesCS7D02G113800	1.4523	1.277	1.29E-05	1.92E-04
TraesCS3B02G176600	1.1023	1.7505	1.29E-05	1.92E-04
TraesCS3B02G315400	-1.168	1.5984	1.29E-05	1.93E-04
TraesCS3D02G142500	1.6965	5.9104	1.31E-05	1.94E-04
TraesCS6D02G396600	1.9333	0.4796	1.31E-05	1.95E-04
TraesCS4B02G117400	2.3848	0.0592	1.31E-05	1.95E-04
TraesCS1D02G206700	1.0059	2.8549	1.31E-05	1.95E-04
TraesCS1B02G225800	-1.9429	0.3672	1.31E-05	1.95E-04
TraesCS4A02G190800	-1.456	1.2156	1.32E-05	1.97E-04
TraesCS1D02G147800	1.1095	1.8059	1.33E-05	1.97E-04
TraesCS7A02G232100	1.5175	1.1671	1.33E-05	1.97E-04
TraesCS4B02G229500	-1.4839	1.015	1.35E-05	2.00E-04
TraesCS7D02G352400	1.5018	6.6735	1.35E-05	2.01E-04
TraesCS2D02G101700	-1.0395	3.0802	1.36E-05	2.01E-04
TraesCS2B02G310500	-1.5321	1.1333	1.37E-05	2.03E-04
TraesCS7D02G385300	-2.2552	0.2718	1.40E-05	2.07E-04
TraesCS1B02G088400	1.3542	4.048	1.42E-05	2.10E-04
TraesCS5A02G064600	1.3265	1.2289	1.42E-05	2.10E-04
TraesCS7D02G174500	1.054	5.5101	1.43E-05	2.10E-04
TraesCS7B02G194500	-1.0444	2.2138	1.43E-05	2.10E-04
TraesCS6A02G085000	1.7617	4.7357	1.43E-05	2.10E-04
TraesCS6D02G241700	2.0299	0.1886	1.45E-05	2.12E-04
TraesCS7D02G313000	-1.232	1.4107	1.47E-05	2.16E-04
TraesCS6A02G131900	-1.3231	1.598	1.48E-05	2.17E-04
TraesCS5A02G238300	-1.1408	2.0777	1.50E-05	2.19E-04
TraesCS1D02G431900	-1.1588	2.3886	1.50E-05	2.19E-04
TraesCS3D02G253000	-3.5386	-0.5107	1.50E-05	2.19E-04
TraesCS2D02G397000	1.1156	1.8088	1.51E-05	2.20E-04
TraesCS7B02G094700	-1.0677	2.0263	1.52E-05	2.22E-04
TraesCS5B02G255400	-1.4522	1.307	1.54E-05	2.25E-04
TraesCS1A02G275000	-1.6728	0.8402	1.56E-05	2.26E-04
TraesCS2B02G164000	-2.1765	0.0996	1.56E-05	2.26E-04
TraesCS3D02G115300	2.5003	-0.1729	1.58E-05	2.30E-04
TraesCS4D02G244800	-1.2147	1.861	1.62E-05	2.34E-04
TraesCS7A02G323100	-1.1066	2.1666	1.62E-05	2.35E-04

TraesCS6D02G296500	-1.166	1.6889	1.62E-05	2.35E-04
TraesCS4B02G177100	-1.0037	2.0449	1.65E-05	2.38E-04
TraesCS4A02G107200	1.058	2.259	1.65E-05	2.38E-04
TraesCS5B02G091800	1.3504	1.0549	1.65E-05	2.38E-04
TraesCS5A02G325200	1.3791	1.1961	1.65E-05	2.38E-04
TraesCS2A02G177900	-1.3549	2.1157	1.65E-05	2.39E-04
TraesCS6A02G139700	-2.0199	0.2264	1.66E-05	2.39E-04
TraesCS3B02G256400	2.3737	-0.0911	1.66E-05	2.39E-04
TraesCS4D02G084900	1.4165	6.0662	1.67E-05	2.41E-04
TraesCS2D02G390200	1.2255	1.4271	1.68E-05	2.41E-04
TraesCS7A02G253000	1.079	5.1062	1.69E-05	2.43E-04
TraesCS7D02G324200	-2.1895	0.2296	1.70E-05	2.44E-04
TraesCS3A02G046500	1.5227	0.8016	1.75E-05	2.50E-04
TraesCS7A02G027000	-1.1801	1.609	1.75E-05	2.50E-04
TraesCS3B02G272600	1.1131	2.5528	1.78E-05	2.54E-04
TraesCS5D02G049700	-2.6394	-0.2291	1.78E-05	2.55E-04
TraesCS7A02G353300	-1.0512	2.1746	1.83E-05	2.61E-04
TraesCS5B02G020700	1.8032	0.4049	1.86E-05	2.64E-04
TraesCS5A02G127700	-1.0848	2.1817	1.86E-05	2.64E-04
TraesCS1D02G112900	-1.2813	1.3488	1.86E-05	2.64E-04
TraesCS2D02G033800	1.0529	2.0077	1.88E-05	2.66E-04
TraesCS5D02G318700	2.1739	0.0529	1.89E-05	2.67E-04
TraesCS3B02G298200	-1.169	1.6674	1.92E-05	2.72E-04
TraesCSU02G139300	-1.1043	1.7068	1.93E-05	2.73E-04
TraesCS6B02G274000	-1.6657	0.829	1.94E-05	2.74E-04
TraesCS2D02G264900	-1.4004	1.0063	1.94E-05	2.74E-04
TraesCS1B02G309200	-1.0758	2.3773	1.95E-05	2.74E-04
TraesCS5B02G056500	-1.2781	1.3878	2.00E-05	2.81E-04
TraesCS7A02G147400	1.0463	2.5384	2.02E-05	2.83E-04
TraesCS7A02G084800	1.6808	0.5461	2.02E-05	2.84E-04
TraesCS2D02G259100	-1.6163	0.797	2.05E-05	2.88E-04
TraesCS7D02G318800	1.1518	2.1393	2.06E-05	2.89E-04
TraesCS5D02G268900	-1.4555	1.857	2.08E-05	2.91E-04
TraesCS6A02G294700	1.6638	0.7187	2.11E-05	2.95E-04
TraesCS5A02G207600	1.241	1.6239	2.11E-05	2.95E-04
TraesCS4D02G192500	-1.1792	1.6517	2.12E-05	2.96E-04
TraesCS4D02G020500	-1.7131	0.8006	2.15E-05	3.00E-04
TraesCS1A02G005500	-1.0828	2.3546	2.16E-05	3.01E-04
TraesCS1A02G283100	-1.0449	2.4005	2.16E-05	3.01E-04
TraesCS1A02G135900	-1.7151	0.5444	2.17E-05	3.02E-04
TraesCS5D02G015700	2.595	0.2107	2.19E-05	3.05E-04
TraesCS5D02G232800	-1.5113	1.9264	2.27E-05	3.14E-04
TraesCS4B02G018000	-1.3018	1.7753	2.29E-05	3.17E-04
TraesCS2D02G103200	1.2937	2.7196	2.29E-05	3.17E-04
TraesCS6D02G335500	-1.1946	1.7511	2.30E-05	3.17E-04
TraesCS6A02G110600	1.3851	1.9277	2.30E-05	3.18E-04
TraesCS7A02G221900	-1.592	0.9971	2.32E-05	3.20E-04
TraesCS7A02G215500	-1.327	1.7086	2.33E-05	3.20E-04

TraesCSU02G012300	-2.3794	0.0979	2.34E-05	3.22E-04
TraesCS4B02G101000	-1.6671	1.8409	2.35E-05	3.23E-04
TraesCS2D02G263500	-1.441	0.8939	2.40E-05	3.28E-04
TraesCS5B02G025100	-1.028	2.2233	2.40E-05	3.28E-04
TraesCS4B02G045400	1.0256	2.4684	2.44E-05	3.34E-04
TraesCS2A02G177800	-1.4802	1.3638	2.45E-05	3.34E-04
TraesCS7B02G055000	-1.1933	2.0239	2.51E-05	3.41E-04
TraesCS5A02G221900	-1.4057	1.4018	2.53E-05	3.44E-04
TraesCS5B02G009100	1.3986	1.5303	2.56E-05	3.47E-04
TraesCS7B02G079100	-1.1951	1.9331	2.57E-05	3.49E-04
TraesCS4D02G197400	2.9023	-0.3165	2.57E-05	3.49E-04
TraesCS1D02G267600	-1.4763	1.1104	2.59E-05	3.51E-04
TraesCSU02G246000	1.3833	1.2011	2.60E-05	3.52E-04
TraesCS1D02G363800	1.3284	2.7347	2.66E-05	3.58E-04
TraesCS1B02G137700	-1.2657	2.117	2.66E-05	3.59E-04
TraesCS5D02G150100	-1.5976	1.0436	2.67E-05	3.59E-04
TraesCS2D02G379600	-1.5482	0.9083	2.72E-05	3.65E-04
TraesCS2B02G114500	-1.1335	1.8646	2.72E-05	3.65E-04
TraesCS2A02G291400	-1.323	1.7509	2.74E-05	3.68E-04
TraesCS2A02G096200	-1.0889	1.7389	2.74E-05	3.68E-04
TraesCS5B02G119300	-1.2614	1.6754	2.75E-05	3.69E-04
TraesCS5B02G344400	-1.3414	1.5974	2.78E-05	3.72E-04
TraesCS7B02G095900	-1.5661	0.8693	2.80E-05	3.75E-04
TraesCS1B02G092100	-1.7535	0.8292	2.83E-05	3.78E-04
TraesCS3D02G183500	-1.5231	1.618	2.84E-05	3.78E-04
TraesCS7B02G138100	1.0964	2.2155	2.87E-05	3.83E-04
TraesCS4D02G049200	-1.3049	1.3649	2.87E-05	3.83E-04
TraesCS5A02G067500	1.0722	5.0014	2.99E-05	3.97E-04
TraesCS7D02G046000	1.3473	1.83	3.03E-05	4.02E-04
TraesCS1A02G241800	-1.2922	1.5114	3.04E-05	4.03E-04
TraesCS5D02G428200	-1.3219	1.5694	3.07E-05	4.06E-04
TraesCS5A02G316400	-1.333	1.2085	3.07E-05	4.06E-04
TraesCS1D02G418100	2.304	0.0058	3.08E-05	4.07E-04
TraesCS5B02G087400	-1.7141	0.5398	3.09E-05	4.08E-04
TraesCS5D02G218700	-1.1849	1.413	3.10E-05	4.09E-04
ENSRNA050017192	-1.3612	1.5058	3.10E-05	4.09E-04
TraesCS2D02G107300	-1.1256	1.5429	3.18E-05	4.18E-04
TraesCS1D02G427900	1.2703	1.8809	3.19E-05	4.19E-04
TraesCS1B02G132000	-1.2443	2.1375	3.20E-05	4.21E-04
TraesCS2A02G111300	1.7705	8.3717	3.27E-05	4.30E-04
TraesCS5A02G105500	-1.3378	1.2091	3.32E-05	4.35E-04
TraesCS2B02G205200	-2.3983	-0.2001	3.33E-05	4.36E-04
TraesCS6A02G079100	-1.3842	1.6216	3.33E-05	4.36E-04
TraesCS5A02G312200	-1.2946	1.831	3.33E-05	4.36E-04
TraesCS3D02G050200	1.9635	0.1455	3.34E-05	4.36E-04
TraesCS2A02G178300	-1.7791	0.8962	3.34E-05	4.37E-04
TraesCS5B02G344700	-2.2303	0.0066	3.35E-05	4.39E-04
TraesCS1D02G138700	-1.2864	1.1749	3.35E-05	4.39E-04

TraesCS2B02G103700	-1.148	2.1089	3.37E-05	4.40E-04
TraesCS7D02G276800	-1.1878	1.6478	3.37E-05	4.40E-04
TraesCS2D02G292400	-1.0541	2.8994	3.38E-05	4.41E-04
TraesCS1D02G250100	-1.0356	2.3313	3.48E-05	4.53E-04
TraesCS3A02G293800	-1.1743	1.622	3.50E-05	4.56E-04
TraesCS6D02G288000	1.5162	3.8229	3.51E-05	4.57E-04
TraesCS2B02G206000	-1.2966	2.2745	3.61E-05	4.69E-04
TraesCS3B02G322900	-2.1925	-0.0173	3.61E-05	4.70E-04
TraesCS5A02G198300	-1.6098	0.7978	3.63E-05	4.72E-04
TraesCS3B02G259500	-1.0489	2.4976	3.66E-05	4.75E-04
TraesCS6D02G030100	1.0174	2.4162	3.66E-05	4.75E-04
TraesCS2A02G062900	-1.2462	4.645	3.75E-05	4.85E-04
ENSRNA050017196	-1.814	0.5414	3.76E-05	4.86E-04
TraesCS2B02G103500	-1.5281	1.0097	3.81E-05	4.92E-04
TraesCS3A02G126300	1.096	2.1793	3.82E-05	4.93E-04
TraesCS4D02G178600	-1.9341	0.3665	3.84E-05	4.96E-04
TraesCS1A02G157900	1.4764	4.6016	3.88E-05	5.00E-04
TraesCS5B02G355900	-1.9991	0.4068	3.89E-05	5.01E-04
TraesCS3D02G306900	1.1769	1.8618	3.89E-05	5.01E-04
TraesCS2B02G102800	-1.3098	1.2544	3.90E-05	5.02E-04
TraesCS4A02G064500	-1.2249	1.6004	3.92E-05	5.04E-04
TraesCS3A02G082900	2.3636	-0.0936	3.92E-05	5.05E-04
TraesCS5D02G354000	-1.3917	1.4337	3.94E-05	5.06E-04
TraesCS2B02G069000	-1.2738	1.6788	3.96E-05	5.08E-04
TraesCS5B02G235200	1.2262	2.2409	3.98E-05	5.11E-04
TraesCS1A02G039800	-1.0556	1.8766	4.02E-05	5.15E-04
TraesCS2D02G374400	1.2435	1.5263	4.02E-05	5.16E-04
TraesCS2B02G003200	-1.6582	0.6374	4.06E-05	5.19E-04
ENSRNA050015989	-2.3793	-0.045	4.11E-05	5.25E-04
TraesCS3D02G229500	-1.1563	1.7747	4.12E-05	5.27E-04
TraesCS4D02G197500	1.1889	1.3434	4.18E-05	5.33E-04
TraesCS4D02G262100	-1.1573	2.4552	4.20E-05	5.36E-04
TraesCS5D02G253200	-1.2894	1.2194	4.22E-05	5.38E-04
TraesCS6D02G244600	1.5067	0.8441	4.30E-05	5.46E-04
TraesCS2B02G233900	-1.3617	1.2132	4.34E-05	5.51E-04
TraesCS5D02G394900	1.1026	1.8365	4.34E-05	5.51E-04
TraesCS3D02G068100	-1.0076	2.6252	4.34E-05	5.51E-04
TraesCS3B02G096000	-1.1969	2.3715	4.42E-05	5.59E-04
TraesCS3D02G418200	-1.1078	2.0413	4.46E-05	5.64E-04
TraesCS5D02G195200	-1.2852	1.2497	4.49E-05	5.66E-04
TraesCS2B02G154600	1.3307	1.235	4.50E-05	5.68E-04
TraesCS2B02G279200	1.2628	1.5713	4.52E-05	5.70E-04
TraesCS3D02G380500	1.4511	0.7001	4.56E-05	5.75E-04
TraesCS2B02G233500	-2.2703	-0.1171	4.61E-05	5.80E-04
TraesCS3D02G350700	1.9042	1.212	4.62E-05	5.81E-04
TraesCS5B02G127500	-1.0528	1.9979	4.64E-05	5.83E-04
TraesCS2D02G391600	-1.018	2.0003	4.67E-05	5.86E-04
TraesCS2B02G002900	-1.2522	1.8286	4.67E-05	5.86E-04

TraesCS4D02G244500	-1.5075	0.6697	4.69E-05	5.88E-04
TraesCSU02G090200	-1.507	0.9439	4.69E-05	5.88E-04
TraesCS1A02G265300	-1.5441	0.9044	4.72E-05	5.91E-04
TraesCS3A02G269400	-1.9259	0.0543	4.75E-05	5.95E-04
TraesCS4B02G064000	-1.8973	0.1423	4.77E-05	5.96E-04
TraesCS3D02G067500	1.0287	2.4737	4.78E-05	5.98E-04
TraesCS3B02G310800	-1.1866	1.4387	4.84E-05	6.05E-04
TraesCS7D02G190900	-1.1235	1.6902	4.85E-05	6.06E-04
TraesCS7A02G257600	1.5708	1.2829	4.87E-05	6.08E-04
ENSRNA050024386	-2.7456	-0.1478	4.88E-05	6.09E-04
TraesCS6D02G249700	1.089	1.6321	4.95E-05	6.17E-04
TraesCS7B02G120900	2.5327	-0.3448	5.00E-05	6.22E-04
TraesCS1D02G241700	-1.085	1.6695	5.03E-05	6.26E-04
TraesCS5D02G405400	-1.4671	0.6971	5.07E-05	6.30E-04
TraesCS2D02G411400	-1.5717	0.9192	5.09E-05	6.32E-04
TraesCS4D02G014300	1.1704	2.9964	5.12E-05	6.36E-04
TraesCS1D02G319400	1.0042	2.5415	5.16E-05	6.41E-04
TraesCS3A02G278300	1.578	1.3994	5.22E-05	6.47E-04
TraesCS7D02G134000	-1.3946	1.1204	5.23E-05	6.48E-04
TraesCS2B02G070800	1.1962	2.5699	5.32E-05	6.58E-04
TraesCS3B02G003800	1.5766	0.9412	5.35E-05	6.61E-04
TraesCS3A02G276600	-1.4231	1.7013	5.37E-05	6.62E-04
ENSRNA050024311	-1.7501	0.5727	5.38E-05	6.63E-04
TraesCSU02G009300	-1.4843	0.9225	5.45E-05	6.70E-04
TraesCS7B02G208800	1.0567	2.5112	5.48E-05	6.73E-04
TraesCS7A02G157300	-1.1632	2.8713	5.55E-05	6.82E-04
TraesCS5D02G061000	-1.3782	0.9967	5.56E-05	6.82E-04
TraesCS2A02G081500	-1.1627	2.5437	5.58E-05	6.85E-04
TraesCS5D02G427100	-1.3547	2.3242	5.59E-05	6.85E-04
TraesCS3D02G142200	1.1656	2.245	5.59E-05	6.85E-04
TraesCS1B02G246900	-1.3694	0.8937	5.60E-05	6.86E-04
TraesCS7A02G231100	1.6586	0.3848	5.61E-05	6.87E-04
TraesCS4B02G077900	-1.9317	0.0511	5.68E-05	6.95E-04
TraesCS3D02G145100	-1.0191	1.7403	5.73E-05	7.00E-04
TraesCS5D02G016100	1.4707	1.1361	5.75E-05	7.01E-04
TraesCS7D02G312200	-1.0381	2.043	5.75E-05	7.01E-04
TraesCS5D02G180900	-1.3586	0.9335	5.92E-05	7.20E-04
TraesCS4A02G084700	1.9528	2.0679	5.97E-05	7.25E-04
TraesCS1D02G272200	1.8094	0.6952	6.09E-05	7.39E-04
TraesCS5B02G111900	1.0917	1.6068	6.11E-05	7.41E-04
TraesCS6B02G275400	1.179	1.7324	6.12E-05	7.42E-04
TraesCS7D02G400500	1.0433	4.083	6.18E-05	7.48E-04
TraesCS3A02G224100	-1.2656	1.1217	6.30E-05	7.62E-04
TraesCS2B02G223300	-1.4976	0.9863	6.31E-05	7.63E-04
TraesCS5D02G497200	1.007	3.7734	6.38E-05	7.71E-04
TraesCS1D02G076000	-1.3263	1.3667	6.41E-05	7.74E-04
TraesCS1B02G240100	-1.8216	0.2916	6.44E-05	7.77E-04
TraesCS7D02G170800	-1.2623	1.1221	6.45E-05	7.77E-04

TraesCS1D02G418600	1.1082	1.9778	6.48E-05	7.81E-04
TraesCS5D02G260500	-1.0982	1.5956	6.51E-05	7.83E-04
TraesCS5B02G336300	1.0707	1.9442	6.59E-05	7.92E-04
TraesCS7D02G089400	1.0341	1.9297	6.70E-05	8.05E-04
TraesCS6D02G131100	-1.2531	1.7909	6.77E-05	8.12E-04
TraesCS6A02G270300	-1.2806	1.1739	6.78E-05	8.14E-04
TraesCS7B02G152200	-1.2863	3.3171	6.84E-05	8.19E-04
TraesCS5A02G286700	-1.2098	1.2657	6.87E-05	8.22E-04
TraesCS4B02G118500	-1.1892	1.1852	6.90E-05	8.26E-04
TraesCS5B02G029300	1.1403	1.3391	6.93E-05	8.28E-04
TraesCS7A02G234300	-1.2663	1.0875	7.00E-05	8.35E-04
TraesCS2D02G034500	1.0656	1.664	7.01E-05	8.36E-04
TraesCS6D02G371400	1.3166	0.9068	7.01E-05	8.36E-04
TraesCS3D02G136000	1.1749	1.3623	7.02E-05	8.37E-04
TraesCS2A02G082600	-1.3384	0.8263	7.05E-05	8.39E-04
TraesCS6B02G117700	-1.9059	0.7403	7.07E-05	8.41E-04
TraesCS5D02G150500	-1.6776	0.4398	7.10E-05	8.44E-04
TraesCS5A02G142500	-1.2321	1.6759	7.14E-05	8.48E-04
TraesCS4B02G226000	-1.2033	1.1216	7.19E-05	8.53E-04
TraesCS4B02G217300	-1.4073	1.0568	7.25E-05	8.61E-04
TraesCS7A02G142500	-2.463	-0.3461	7.29E-05	8.64E-04
TraesCS3B02G257400	1.0022	2.7512	7.33E-05	8.69E-04
TraesCS3B02G130000	1.1571	1.5231	7.37E-05	8.72E-04
TraesCS7D02G049400	-1.2054	1.2285	7.41E-05	8.76E-04
TraesCS2D02G089300	2.1885	-0.0698	7.50E-05	8.86E-04
TraesCSU02G113500	-1.1334	1.8166	7.50E-05	8.86E-04
TraesCS1D02G164900	-1.4574	0.8021	7.53E-05	8.89E-04
TraesCS4D02G314100	1.5134	0.6876	7.59E-05	8.95E-04
TraesCS5D02G360500	1.3253	0.9571	7.59E-05	8.95E-04
TraesCS7B02G109700	-1.2263	1.2351	7.63E-05	8.99E-04
TraesCS7D02G417600	1.1167	1.6474	7.67E-05	9.02E-04
TraesCS4A02G222700	-1.1375	1.3174	7.67E-05	9.02E-04
TraesCS4B02G200800	1.3099	5.1552	7.75E-05	9.09E-04
TraesCS3D02G155200	1.1954	1.9238	7.76E-05	9.10E-04
TraesCS4D02G072800	-1.0776	1.9988	7.81E-05	9.15E-04
TraesCS3A02G190200	1.4424	1.1509	7.83E-05	9.17E-04
TraesCS1A02G278700	-1.3299	1.1228	7.89E-05	9.23E-04
TraesCS1A02G236900	-1.3748	0.8004	8.02E-05	9.37E-04
ENSRNA050024387	-1.305	2.0214	8.09E-05	9.43E-04
TraesCS1B02G074100	1.5982	0.4927	8.10E-05	9.44E-04
TraesCS1A02G082300	-1.669	0.5106	8.19E-05	9.52E-04
TraesCS7B02G212000	1.418	1.7334	8.22E-05	9.55E-04
TraesCS7D02G015700	1.038	2.8113	8.33E-05	9.66E-04
TraesCS7D02G363200	-1.1111	1.5535	8.35E-05	9.68E-04
TraesCS1A02G217100	1.1961	1.4895	8.40E-05	9.73E-04
TraesCS7A02G152600	-1.3238	1.0859	8.44E-05	9.77E-04
TraesCS5B02G105900	-1.7998	0.3701	8.45E-05	9.78E-04
TraesCS2A02G282200	-1.4834	0.5916	8.46E-05	9.78E-04

TraesCS2D02G001000	-1.1719	1.1747	8.51E-05	9.83E-04
TraesCS1B02G161000	-1.4488	0.7407	8.75E-05	1.01E-03
TraesCS7A02G256900	1.3908	0.9611	8.79E-05	1.01E-03
TraesCSU02G046800	1.0543	2.3325	8.83E-05	1.02E-03
TraesCS5A02G050200	-1.137	1.8349	8.84E-05	1.02E-03
TraesCS6B02G096400	1.0203	1.8053	8.85E-05	1.02E-03
TraesCS1A02G005400	1.3992	0.7703	8.87E-05	1.02E-03
TraesCS7D02G326000	-1.0114	1.764	8.90E-05	1.02E-03
TraesCS2A02G294200	-1.2066	1.9898	8.93E-05	1.03E-03
TraesCS6A02G278100	1.3602	0.8443	9.02E-05	1.04E-03
TraesCS4B02G056600	1.0375	1.6684	9.05E-05	1.04E-03
TraesCS4D02G065400	-1.0881	1.9617	9.06E-05	1.04E-03
TraesCS2D02G222100	2.1162	-0.1164	9.22E-05	1.06E-03
TraesCS6A02G053000	1.1122	4.4621	9.33E-05	1.07E-03
TraesCS5A02G248600	-1.5203	0.7317	9.62E-05	1.10E-03
TraesCS3D02G119800	-1.1179	2.0405	9.64E-05	1.10E-03
TraesCS7D02G258300	-1.4699	0.9557	9.66E-05	1.10E-03
TraesCS5B02G279200	1.3317	1.1175	9.69E-05	1.10E-03
TraesCS2A02G229100	-1.3869	1.1704	9.73E-05	1.11E-03
TraesCS2A02G253300	1.828	0.251	9.80E-05	1.11E-03
TraesCS2B02G210600	1.2205	1.4265	9.81E-05	1.12E-03
TraesCS3D02G243300	-1.3495	1.0557	9.85E-05	1.12E-03
TraesCS3A02G129800	1.1557	1.4951	9.87E-05	1.12E-03
TraesCS3A02G280400	-1.8414	0.2086	9.89E-05	1.12E-03
TraesCS5A02G219200	1.0579	2.0566	9.97E-05	1.13E-03
TraesCS2B02G140300	1.0725	2.0815	1.01E-04	1.14E-03
TraesCS1D02G367700	-1.0319	1.6572	1.01E-04	1.14E-03
TraesCSU02G056200	-1.0041	2.3753	1.01E-04	1.14E-03
TraesCS1B02G277000	-1.4857	0.7149	1.01E-04	1.14E-03
TraesCS7A02G068300	-1.4116	1.2528	1.03E-04	1.16E-03
ENSRNA050020650	-2.5554	-0.2873	1.03E-04	1.16E-03
TraesCS5D02G464500	1.2155	1.869	1.03E-04	1.16E-03
TraesCS6D02G247100	-1.38	0.6998	1.03E-04	1.16E-03
TraesCS2A02G178200	-1.722	0.3096	1.05E-04	1.18E-03
TraesCS2D02G109500	2.0571	-0.0181	1.07E-04	1.20E-03
TraesCS6A02G034300	-1.6045	0.8367	1.08E-04	1.21E-03
TraesCS4A02G106500	-1.0648	1.6082	1.09E-04	1.22E-03
TraesCS1D02G352100	1.4259	0.6845	1.09E-04	1.22E-03
TraesCS7D02G344900	-1.2346	1.8618	1.09E-04	1.22E-03
TraesCS3A02G148600	-2.076	0.3704	1.10E-04	1.22E-03
TraesCS7A02G213700	1.5	0.8347	1.10E-04	1.23E-03
TraesCS5D02G348900	2.1093	-0.1198	1.14E-04	1.26E-03
TraesCS5B02G048500	-2.0289	0.007	1.14E-04	1.27E-03
TraesCS2D02G390700	-1.4797	0.5254	1.15E-04	1.28E-03
TraesCS2D02G194600	-1.0036	1.9118	1.15E-04	1.28E-03
TraesCS1A02G226700	-1.121	2.0694	1.16E-04	1.29E-03
TraesCS4B02G256500	1.7348	0.1907	1.17E-04	1.29E-03
TraesCS7A02G324500	-1.3328	1.5632	1.17E-04	1.30E-03

TraesCS7B02G148200	-1.1615	1.2362	1.18E-04	1.31E-03
TraesCS3D02G339700	-1.337	0.9176	1.19E-04	1.31E-03
TraesCS7B02G161800	-1.2689	1.0561	1.21E-04	1.34E-03
TraesCS7B02G083100	1.3712	0.9387	1.21E-04	1.34E-03
TraesCS2D02G221600	1.4935	0.6098	1.22E-04	1.34E-03
TraesCS3D02G247900	1.3051	1.4906	1.22E-04	1.35E-03
TraesCS5B02G289200	1.0415	1.7589	1.24E-04	1.36E-03
TraesCS5B02G292400	1.3549	1.0956	1.24E-04	1.36E-03
TraesCS7B02G226600	-1.6547	0.2698	1.24E-04	1.36E-03
TraesCS6D02G112000	-1.8264	0.1	1.24E-04	1.36E-03
TraesCS4B02G211700	1.6643	0.3098	1.24E-04	1.36E-03
TraesCS2A02G152200	1.3262	0.8711	1.25E-04	1.37E-03
TraesCS5B02G326100	-1.1183	1.5745	1.25E-04	1.38E-03
TraesCS5B02G217000	1.0438	1.6035	1.27E-04	1.39E-03
TraesCS5B02G130100	-1.0102	1.6701	1.27E-04	1.39E-03
TraesCS1B02G053900	-1.7615	0.2482	1.28E-04	1.40E-03
TraesCS6A02G071500	1.0746	1.8006	1.29E-04	1.41E-03
TraesCS7B02G228500	1.0066	4.4013	1.29E-04	1.41E-03
TraesCS4A02G218000	1.609	0.4247	1.29E-04	1.41E-03
TraesCS1D02G148700	1.8597	0.9124	1.31E-04	1.43E-03
TraesCS4B02G107000	-1.2105	1.2366	1.32E-04	1.43E-03
TraesCS2B02G017200	-2.0908	-0.092	1.34E-04	1.46E-03
TraesCS3D02G158800	1.0549	2.018	1.34E-04	1.46E-03
TraesCS6D02G134000	-1.2539	1.1189	1.35E-04	1.46E-03
TraesCS2D02G350100	1.9127	0.7752	1.36E-04	1.47E-03
TraesCS2A02G210100	-1.1865	1.188	1.38E-04	1.49E-03
TraesCS7B02G233900	-1.8856	0.3318	1.39E-04	1.49E-03
TraesCS3A02G227800	1.0435	1.5003	1.39E-04	1.50E-03
TraesCS5B02G317500	1.0832	1.7356	1.39E-04	1.50E-03
TraesCS3D02G340900	-1.2359	1.2575	1.40E-04	1.51E-03
TraesCS2B02G325800	-1.5684	0.575	1.40E-04	1.51E-03
TraesCS1D02G288200	1.6565	1.093	1.42E-04	1.53E-03
TraesCS6D02G337000	-1.4745	1.0118	1.44E-04	1.55E-03
TraesCS3D02G114900	2.4959	-0.1751	1.45E-04	1.55E-03
TraesCS7A02G222500	-1.1706	1.0638	1.45E-04	1.55E-03
TraesCS4D02G357300	-1.6202	0.4055	1.46E-04	1.57E-03
TraesCS3B02G041400	-1.0222	1.6791	1.46E-04	1.57E-03
TraesCS4D02G190100	1.1558	1.6005	1.47E-04	1.57E-03
TraesCS7D02G242600	-1.387	0.7554	1.47E-04	1.58E-03
TraesCS7B02G209600	1.5295	0.7585	1.47E-04	1.58E-03
TraesCS4A02G062600	1.198	3.0103	1.49E-04	1.59E-03
TraesCS5B02G085600	-1.761	0.5076	1.49E-04	1.59E-03
TraesCSU02G036400	1.2236	0.9347	1.49E-04	1.59E-03
TraesCS7D02G340300	-1.8992	0.14	1.50E-04	1.60E-03
TraesCS3A02G189200	-1.0911	1.8256	1.51E-04	1.61E-03
TraesCS5D02G202800	-1.3458	0.7281	1.51E-04	1.61E-03
TraesCS5B02G143700	1.0232	1.9883	1.52E-04	1.62E-03
TraesCS2B02G095800	1.4105	0.8327	1.52E-04	1.62E-03

TraesCS1B02G252600	-1.1914	1.1544	1.54E-04	1.63E-03
TraesCS5D02G253100	-1.4837	0.5885	1.54E-04	1.63E-03
TraesCS1D02G282600	-1.0044	1.9858	1.54E-04	1.64E-03
TraesCS5A02G178700	-1.2075	1.0869	1.54E-04	1.64E-03
TraesCS5D02G441600	-1.3079	0.8528	1.55E-04	1.65E-03
TraesCS3B02G289400	1.263	2.309	1.56E-04	1.65E-03
TraesCS6D02G373000	-1.3754	1.6	1.57E-04	1.66E-03
TraesCS3D02G315000	-2.0067	-0.1457	1.59E-04	1.68E-03
TraesCS2D02G007800	-2.0512	-0.1204	1.59E-04	1.68E-03
TraesCS5D02G351600	-1.9142	0.5961	1.61E-04	1.69E-03
TraesCS7D02G367300	1.924	0.0075	1.61E-04	1.70E-03
TraesCS7D02G251000	1.0034	1.6673	1.63E-04	1.72E-03
TraesCS2A02G046800	1.0901	1.8903	1.65E-04	1.73E-03
TraesCS2B02G223800	-1.1866	1.6231	1.67E-04	1.76E-03
TraesCS7A02G326000	-1.4849	0.7565	1.68E-04	1.76E-03
TraesCS6A02G247800	-1.426	0.9732	1.68E-04	1.76E-03
TraesCS4D02G291700	1.0251	2.2581	1.68E-04	1.76E-03
TraesCS2B02G299100	-1.7737	0.3432	1.69E-04	1.77E-03
TraesCS4A02G082500	-1.0338	1.9357	1.70E-04	1.77E-03
TraesCS7D02G239600	-1.0577	1.7421	1.72E-04	1.79E-03
TraesCS5A02G049000	-1.5698	0.7027	1.74E-04	1.81E-03
TraesCS1D02G233900	-1.9826	0.2135	1.77E-04	1.84E-03
TraesCS4B02G056300	1.3145	1.114	1.77E-04	1.85E-03
TraesCS1A02G203200	1.1873	3.9281	1.80E-04	1.87E-03
TraesCS7B02G280800	1.2793	1.7069	1.82E-04	1.88E-03
TraesCS2B02G293600	-1.2392	1.285	1.82E-04	1.89E-03
TraesCS4D02G344200	1.0532	1.7909	1.83E-04	1.90E-03
TraesCS3A02G109400	1.6625	0.2311	1.84E-04	1.90E-03
TraesCS1B02G216300	-1.016	1.9756	1.88E-04	1.94E-03
TraesCS1B02G255300	1.7689	0.21	1.89E-04	1.95E-03
TraesCS4D02G051600	-1.3167	0.8137	1.90E-04	1.96E-03
TraesCSU02G029900	-1.0035	1.9688	1.90E-04	1.96E-03
TraesCS3B02G284000	-1.6315	0.8062	1.90E-04	1.97E-03
TraesCS6A02G094000	1.0655	1.7997	1.91E-04	1.97E-03
TraesCS7A02G327800	1.2088	5.063	1.92E-04	1.98E-03
TraesCS7D02G234500	-1.0552	1.383	1.98E-04	2.04E-03
TraesCS5B02G229100	-1.6145	0.6047	2.00E-04	2.06E-03
TraesCS1B02G060200	1.0585	1.3497	2.01E-04	2.06E-03
TraesCS1B02G155800	-1.0487	1.6547	2.02E-04	2.07E-03
TraesCS5A02G086200	-1.7562	0.2516	2.02E-04	2.07E-03
TraesCS1B02G052900	-1.09	1.5193	2.12E-04	2.17E-03
TraesCS4B02G184400	1.0842	7.2941	2.14E-04	2.18E-03
TraesCS4D02G097600	-1.1123	1.8911	2.14E-04	2.18E-03
TraesCS7D02G367400	1.732	0.1881	2.16E-04	2.20E-03
TraesCS3B02G016500	-1.1944	1.478	2.17E-04	2.21E-03
TraesCS7A02G280900	-2.0984	-0.0936	2.18E-04	2.21E-03
TraesCS1D02G384300	1.9545	-0.0881	2.20E-04	2.23E-03
TraesCS4A02G041200	-1.0729	2.1023	2.21E-04	2.24E-03

TraesCS7D02G405200	-1.056	1.9571	2.21E-04	2.25E-03
TraesCS1D02G371700	-1.0322	2.7069	2.23E-04	2.26E-03
TraesCS5A02G130900	-1.1807	1.2164	2.24E-04	2.27E-03
TraesCS4A02G117500	-1.1839	0.9919	2.24E-04	2.27E-03
TraesCS7B02G052400	-1.3659	0.7428	2.25E-04	2.27E-03
TraesCS5D02G082200	-1.4357	0.8825	2.25E-04	2.28E-03
TraesCS6D02G281100	-1.6632	0.1892	2.26E-04	2.29E-03
TraesCS5A02G316900	1.3284	2.9508	2.28E-04	2.30E-03
TraesCS2D02G280700	-1.1681	1.5336	2.29E-04	2.31E-03
TraesCS7D02G390000	1.2732	1.2584	2.30E-04	2.32E-03
TraesCS4A02G067000	-1.4205	0.6666	2.30E-04	2.32E-03
TraesCS1B02G234900	1.793	2.3262	2.31E-04	2.33E-03
TraesCS1D02G348500	1.0634	1.2927	2.33E-04	2.34E-03
TraesCS5D02G120600	1.2418	0.9841	2.34E-04	2.35E-03
TraesCS3A02G098800	-1.6262	0.2503	2.34E-04	2.35E-03
TraesCS2A02G129200	-1.7877	0.1654	2.34E-04	2.35E-03
TraesCS3D02G220700	-1.0657	2.0657	2.37E-04	2.38E-03
TraesCS1A02G001400	-1.6806	1.535	2.38E-04	2.38E-03
TraesCS5A02G079400	1.0125	1.8957	2.39E-04	2.39E-03
TraesCS5B02G099000	-1.3573	1.0271	2.39E-04	2.39E-03
TraesCS6A02G285200	1.7431	0.1024	2.41E-04	2.40E-03
TraesCS4A02G085300	1.2075	2.3505	2.41E-04	2.41E-03
TraesCS7B02G096600	-1.4977	1.4641	2.42E-04	2.42E-03
TraesCS2B02G311000	1.1346	2.0758	2.43E-04	2.42E-03
TraesCS7B02G170400	1.1375	3.953	2.44E-04	2.43E-03
TraesCS5A02G061100	1.0507	1.6486	2.47E-04	2.46E-03
TraesCS5D02G239000	-1.0141	1.903	2.47E-04	2.46E-03
TraesCS5A02G219100	1.432	0.5775	2.49E-04	2.47E-03
TraesCS6A02G172900	-1.9744	-0.0412	2.52E-04	2.50E-03
TraesCS5D02G101500	-1.0989	1.5302	2.52E-04	2.50E-03
TraesCS3A02G135400	1.1081	1.2269	2.53E-04	2.51E-03
TraesCS4B02G114700	1.1578	3.6907	2.54E-04	2.52E-03
TraesCSU02G116200	-2.2117	-0.3153	2.56E-04	2.54E-03
TraesCS2D02G165300	-1.5837	0.3819	2.61E-04	2.58E-03
TraesCS3B02G271400	-1.223	1.1354	2.63E-04	2.60E-03
TraesCS6A02G143700	2.95	-0.5752	2.63E-04	2.60E-03
TraesCS7A02G122300	-1.0219	1.3944	2.68E-04	2.64E-03
TraesCS5D02G361500	-1.6418	0.3475	2.69E-04	2.65E-03
TraesCS7B02G004600	1.0152	1.4326	2.71E-04	2.67E-03
TraesCS2B02G137500	-2.0523	0.0306	2.72E-04	2.67E-03
TraesCS4B02G261200	-1.0571	1.8707	2.72E-04	2.68E-03
TraesCS2D02G381700	-1.2682	0.8255	2.77E-04	2.71E-03
TraesCS3B02G160300	1.1882	4.3698	2.81E-04	2.74E-03
TraesCS4D02G346800	-1.0592	1.4251	2.82E-04	2.75E-03
TraesCS6D02G225800	1.071	5.2922	2.83E-04	2.76E-03
TraesCS4B02G184900	1.268	1.0062	2.85E-04	2.77E-03
TraesCS5D02G427800	-1.2165	0.9675	2.87E-04	2.80E-03
TraesCS2D02G043300	-2.2125	-0.3168	2.90E-04	2.82E-03

TraesCS1B02G242600	-1.4525	0.5061	2.92E-04	2.83E-03
TraesCS3D02G349400	-1.5417	0.6226	2.93E-04	2.84E-03
TraesCS7A02G134600	-1.0288	1.4835	2.95E-04	2.85E-03
TraesCS6D02G225100	-1.1378	1.2557	3.00E-04	2.90E-03
TraesCS1D02G190800	-1.3636	0.5763	3.00E-04	2.90E-03
TraesCS2D02G325200	-1.2695	1.1604	3.02E-04	2.92E-03
TraesCS6B02G295100	-1.4386	0.5598	3.03E-04	2.92E-03
TraesCS1A02G013500	-2.2562	-0.2851	3.05E-04	2.94E-03
TraesCS7D02G036700	-1.0435	1.2581	3.06E-04	2.95E-03
TraesCS3B02G109300	-1.5013	0.6519	3.08E-04	2.97E-03
TraesCS7B02G097100	-1.5263	0.5405	3.12E-04	2.99E-03
TraesCS7D02G150200	-1.7553	0.0526	3.12E-04	3.00E-03
TraesCS1A02G123200	-1.2597	1.1629	3.13E-04	3.00E-03
TraesCS3D02G102200	2.065	-0.1462	3.13E-04	3.00E-03
TraesCS2A02G184100	-1.7432	0.1473	3.15E-04	3.02E-03
TraesCS6A02G204600	-1.0613	1.3968	3.16E-04	3.03E-03
TraesCS5B02G312800	1.3525	1.2195	3.18E-04	3.05E-03
TraesCS2B02G121600	-1.315	1.4933	3.20E-04	3.07E-03
TraesCS7A02G348800	-1.6364	0.7641	3.22E-04	3.07E-03
TraesCS2D02G312200	1.5105	1.0421	3.23E-04	3.09E-03
TraesCS1B02G174100	1.2372	4.3889	3.27E-04	3.12E-03
TraesCS4B02G116800	-1.0562	1.2706	3.31E-04	3.14E-03
TraesCS6A02G293700	-1.9947	-0.1466	3.31E-04	3.15E-03
TraesCS2D02G157500	1.4233	0.5088	3.35E-04	3.18E-03
TraesCS5B02G187600	-1.51	0.8682	3.40E-04	3.22E-03
TraesCS1A02G314600	1.3879	0.5443	3.44E-04	3.25E-03
TraesCS6D02G352300	1.0284	1.9014	3.48E-04	3.29E-03
TraesCS7A02G160200	1.1965	1.3428	3.52E-04	3.32E-03
TraesCS1D02G263500	-2.0571	0.1248	3.53E-04	3.32E-03
TraesCS6B02G175000	-1.6715	0.1886	3.54E-04	3.33E-03
TraesCS5D02G082700	1.2274	1.3035	3.56E-04	3.35E-03
TraesCS7A02G229300	1.0976	1.4573	3.63E-04	3.40E-03
TraesCS7A02G051400	-1.9187	-0.2015	3.63E-04	3.41E-03
TraesCS7A02G178700	-1.3775	0.7579	3.64E-04	3.41E-03
TraesCS1D02G419900	-1.1959	0.9199	3.68E-04	3.44E-03
TraesCS4D02G058200	1.2221	1.5436	3.69E-04	3.45E-03
TraesCS3A02G040900	-1.4379	0.5621	3.71E-04	3.46E-03
TraesCS3A02G190100	1.0544	3.7347	3.74E-04	3.49E-03
TraesCS6A02G014800	-1.4112	0.61	3.74E-04	3.49E-03
TraesCS7A02G165700	-1.0524	1.4668	3.77E-04	3.51E-03
TraesCS5D02G491000	1.444	0.459	3.79E-04	3.53E-03
TraesCS1A02G001100	-1.5265	1.7679	3.83E-04	3.56E-03
TraesCS3D02G079100	-1.6531	0.4917	3.85E-04	3.57E-03
TraesCS4D02G073300	-1.0878	2.7928	3.86E-04	3.58E-03
TraesCS1B02G087000	-1.6824	0.4536	3.90E-04	3.61E-03
TraesCS7A02G216300	-1.0016	1.3863	3.93E-04	3.63E-03
TraesCS6A02G059700	1.7113	0.0798	3.96E-04	3.66E-03
TraesCS3D02G367200	1.6716	0.3275	3.98E-04	3.67E-03

TraesCS6D02G243300	-1.5925	0.2292	4.00E-04	3.69E-03
TraesCS5B02G282300	1.731	0.4307	4.00E-04	3.69E-03
TraesCS1B02G195500	1.4512	0.704	4.01E-04	3.69E-03
TraesCS3D02G208500	-1.0858	1.9119	4.02E-04	3.70E-03
TraesCS7D02G413300	1.8264	0.0552	4.02E-04	3.70E-03
TraesCS4B02G210900	-1.0363	1.9318	4.02E-04	3.71E-03
TraesCS4B02G063800	-1.074	1.6638	4.05E-04	3.72E-03
TraesCS4A02G127300	-1.7535	0.0552	4.06E-04	3.73E-03
TraesCS1D02G223400	1.3722	1.6329	4.06E-04	3.73E-03
TraesCS2B02G243800	-1.5925	0.231	4.07E-04	3.74E-03
TraesCSU02G112000	-1.5562	0.2081	4.12E-04	3.77E-03
TraesCS5B02G236100	-1.0237	1.8733	4.20E-04	3.84E-03
TraesCS1B02G120000	-1.098	2.0539	4.23E-04	3.86E-03
TraesCS7B02G093400	1.5963	0.1863	4.25E-04	3.88E-03
TraesCS5D02G254600	1.7265	1.031	4.26E-04	3.88E-03
TraesCS3B02G041300	-1.2918	0.9765	4.29E-04	3.91E-03
TraesCS2B02G265700	-1.3715	1.0769	4.31E-04	3.93E-03
TraesCS6D02G309600	1.2234	1.2346	4.31E-04	3.93E-03
TraesCS3A02G085900	-1.2239	1.0211	4.34E-04	3.95E-03
TraesCS4B02G176900	-1.1418	1.2565	4.34E-04	3.95E-03
ENSRNA050020645	-1.6829	0.3651	4.36E-04	3.97E-03
TraesCS1D02G439200	1.6294	0.3711	4.37E-04	3.97E-03
TraesCS5D02G346000	-1.2023	1.6739	4.41E-04	4.00E-03
TraesCS6A02G285600	1.004	1.4269	4.43E-04	4.01E-03
TraesCS3B02G160000	1.0325	1.6919	4.44E-04	4.02E-03
TraesCS4B02G016500	1.8243	0.054	4.46E-04	4.04E-03
TraesCS3D02G349100	1.4743	0.6694	4.50E-04	4.07E-03
TraesCS5A02G188200	-1.722	0.0326	4.51E-04	4.07E-03
TraesCSU02G024700	-1.3479	0.9682	4.51E-04	4.07E-03
TraesCS1D02G028200	-2.0021	-0.1481	4.51E-04	4.07E-03
TraesCS4D02G213900	1.311	1.8126	4.52E-04	4.08E-03
TraesCS4B02G028600	1.0032	1.852	4.53E-04	4.08E-03
TraesCS6D02G099300	1.2608	0.912	4.56E-04	4.11E-03
TraesCSU02G122100	2.3901	-0.4383	4.59E-04	4.13E-03
TraesCS3B02G042700	1.3758	0.8575	4.60E-04	4.14E-03
TraesCS5A02G191700	1.0224	3.7414	4.62E-04	4.15E-03
TraesCS7D02G242400	-1.8191	0.1893	4.63E-04	4.16E-03
TraesCS5D02G325500	-1.503	0.3264	4.64E-04	4.16E-03
TraesCS6B02G073200	-1.3856	0.5935	4.66E-04	4.18E-03
TraesCS7D02G358400	-1.6221	0.5543	4.66E-04	4.18E-03
TraesCS3A02G063200	-1.7801	-0.041	4.67E-04	4.19E-03
TraesCS1B02G156200	-1.2331	0.9473	4.71E-04	4.21E-03
TraesCS2A02G054000	-1.5363	0.3458	4.71E-04	4.22E-03
TraesCSU02G221100	-1.1773	1.5691	4.72E-04	4.23E-03
TraesCS2A02G124300	1.3896	2.0249	4.77E-04	4.26E-03
TraesCS1D02G279800	-1.0515	1.6039	4.83E-04	4.31E-03
TraesCS1D02G206800	-1.3421	0.5572	4.84E-04	4.31E-03
TraesCS2A02G056700	-1.1985	1.0438	4.84E-04	4.31E-03

TraesCSU02G120800	1.1856	2.5776	4.85E-04	4.32E-03
TraesCS2D02G002600	-1.3314	0.8801	4.89E-04	4.35E-03
TraesCS2D02G186500	-1.2959	1.5711	4.90E-04	4.36E-03
TraesCS3B02G043400	1.1442	1.2391	4.91E-04	4.36E-03
TraesCS1D02G160700	1.4539	0.3316	4.92E-04	4.37E-03
TraesCS6D02G144300	-1.0442	1.5955	4.96E-04	4.40E-03
TraesCS1A02G221700	1.245	1.8054	4.96E-04	4.40E-03
TraesCS2B02G004500	-1.0436	1.2304	4.97E-04	4.41E-03
TraesCS1D02G382200	1.5532	0.5283	5.00E-04	4.43E-03
TraesCS5A02G205200	-1.205	1.0782	5.00E-04	4.43E-03
TraesCS5D02G057300	-1.2574	0.905	5.00E-04	4.43E-03
TraesCS5D02G068400	1.3883	0.6066	5.00E-04	4.43E-03
TraesCS6A02G246700	-1.2628	1.3039	5.03E-04	4.45E-03
TraesCS5D02G388800	-1.674	0.0988	5.03E-04	4.45E-03
TraesCS6A02G120600	-1.3343	1.1348	5.04E-04	4.46E-03
TraesCS1A02G203300	-1.6564	0.186	5.19E-04	4.58E-03
TraesCS2B02G078600	-1.5616	0.1222	5.19E-04	4.58E-03
TraesCS3B02G090000	1.7329	0.2852	5.24E-04	4.61E-03
TraesCS7B02G207800	-1.0329	1.3505	5.25E-04	4.62E-03
TraesCS6A02G250000	1.3561	0.5798	5.28E-04	4.64E-03
TraesCS2D02G324700	-1.1803	1.4936	5.34E-04	4.69E-03
TraesCS2B02G336300	1.3954	0.7636	5.37E-04	4.71E-03
TraesCS1A02G277700	-1.1924	1.3129	5.40E-04	4.73E-03
TraesCS6A02G282600	2.1227	-0.2573	5.43E-04	4.75E-03
TraesCS5A02G179300	1.1237	3.7679	5.45E-04	4.76E-03
TraesCS7A02G270000	-1.1646	1.1328	5.46E-04	4.77E-03
TraesCS1B02G108400	1.2152	0.8016	5.54E-04	4.83E-03
TraesCS3A02G180000	-1.5361	0.6881	5.58E-04	4.86E-03
TraesCS7B02G274900	-1.56	0.2075	5.61E-04	4.89E-03
TraesCS4A02G091600	1.0423	2.2475	5.64E-04	4.91E-03
TraesCS4D02G124800	-1.4292	0.7915	5.65E-04	4.92E-03
ENSRNA050018907	-2.0451	2.867	5.65E-04	4.92E-03
TraesCS1D02G408500	1.5525	0.9654	5.75E-04	4.99E-03
TraesCS1D02G069800	1.6931	0.1655	5.82E-04	5.04E-03
TraesCS3D02G231500	-1.1406	1.1556	5.84E-04	5.05E-03
TraesCS1D02G351600	-1.4556	0.4389	5.86E-04	5.07E-03
TraesCS2A02G299600	-1.5983	0.0548	5.90E-04	5.09E-03
TraesCS5A02G101000	-1.7855	-0.0445	5.91E-04	5.10E-03
ENSRNA050016007	-1.1682	1.4591	5.93E-04	5.11E-03
TraesCS4B02G219400	-1.5274	0.2669	5.97E-04	5.14E-03
TraesCS2A02G019900	1.7561	0.0085	5.99E-04	5.16E-03
TraesCS1B02G169300	1.4374	0.3845	5.99E-04	5.16E-03
TraesCS1D02G139800	1.1917	0.9109	6.04E-04	5.19E-03
TraesCS5B02G017300	-1.2048	0.7423	6.06E-04	5.21E-03
ENSRNA050016398	-1.6873	1.5733	6.06E-04	5.21E-03
TraesCS5D02G004500	1.3932	0.494	6.09E-04	5.23E-03
TraesCS5D02G130800	-1.9569	-0.3153	6.11E-04	5.24E-03
TraesCS7B02G291100	-1.5091	0.9746	6.15E-04	5.27E-03

TraesCS1A02G301100	-1.0735	1.741	6.20E-04	5.32E-03
TraesCS7B02G128800	1.3337	0.7785	6.23E-04	5.34E-03
ENSRNA050021227	-1.6082	0.3263	6.32E-04	5.40E-03
TraesCS6B02G190000	1.6383	1.5334	6.37E-04	5.44E-03
TraesCS4D02G331600	-1.4901	0.459	6.37E-04	5.44E-03
TraesCS5A02G073100	-1.0466	1.7457	6.39E-04	5.45E-03
ENSRNA050020728	-1.7124	0.8091	6.43E-04	5.48E-03
TraesCS3B02G144900	1.0933	1.1546	6.46E-04	5.50E-03
TraesCS7B02G248700	-1.0963	1.9645	6.49E-04	5.53E-03
TraesCS4A02G156700	-1.1674	1.095	6.50E-04	5.53E-03
TraesCS2A02G210400	-1.1827	1.1156	6.57E-04	5.58E-03
TraesCS5B02G240000	-1.3378	0.9582	6.59E-04	5.59E-03
TraesCS7D02G310800	-1.0684	1.2789	6.62E-04	5.62E-03
TraesCS5B02G267800	1.2014	0.7877	6.63E-04	5.62E-03
TraesCS3B02G166900	1.0164	5.5034	6.68E-04	5.66E-03
TraesCS5D02G190200	-1.5599	0.1241	6.69E-04	5.67E-03
TraesCS5D02G352100	-1.2871	1.2993	6.70E-04	5.67E-03
TraesCS5A02G064900	1.3755	1.4664	6.70E-04	5.67E-03
TraesCS5B02G161200	-1.229	0.9328	6.73E-04	5.69E-03
TraesCS7D02G274800	-1.7346	-0.068	6.75E-04	5.71E-03
TraesCS1D02G115800	-1.0716	1.144	6.76E-04	5.72E-03
TraesCS1A02G270900	-1.4126	0.7701	6.77E-04	5.73E-03
TraesCS5D02G403800	-1.4982	0.2475	6.79E-04	5.74E-03
TraesCS2D02G081500	-1.0439	1.4114	6.81E-04	5.75E-03
TraesCS7D02G094300	-1.5418	0.561	6.91E-04	5.83E-03
TraesCS7A02G008300	-1.0197	1.7441	6.94E-04	5.85E-03
TraesCS6D02G263100	1.7172	0.4992	6.99E-04	5.89E-03
TraesCS2A02G251000	-1.4067	0.4721	7.10E-04	5.97E-03
TraesCS4D02G243000	1.3377	0.4552	7.17E-04	6.02E-03
TraesCS4A02G204900	-1.4609	0.2283	7.18E-04	6.02E-03
TraesCS5D02G186700	-1.0971	1.5811	7.26E-04	6.08E-03
TraesCS2D02G100600	-1.2351	0.9969	7.38E-04	6.16E-03
TraesCS4D02G063500	1.0509	1.423	7.39E-04	6.17E-03
TraesCS6D02G379200	1.4314	1.5369	7.40E-04	6.17E-03
TraesCS2A02G178400	-1.8408	-0.1205	7.40E-04	6.18E-03
TraesCS1A02G314700	1.334	0.5105	7.42E-04	6.19E-03
TraesCS6B02G174300	-1.1515	1.231	7.42E-04	6.19E-03
TraesCS5A02G014900	-1.0844	1.118	7.46E-04	6.21E-03
TraesCS4D02G336100	-1.4561	0.4416	7.51E-04	6.25E-03
TraesCS2D02G013100	1.7645	-0.0896	7.52E-04	6.26E-03
TraesCS1A02G031100	-1.1114	1.0666	7.55E-04	6.28E-03
TraesCSU02G109700	1.1275	0.9931	7.56E-04	6.28E-03
TraesCS7A02G337300	-1.0445	2.3099	7.59E-04	6.30E-03
TraesCS7D02G329600	-1.4985	0.2518	7.59E-04	6.30E-03
TraesCS3D02G159200	-1.1	1.0172	7.59E-04	6.30E-03
TraesCS6D02G007900	-1.0339	1.052	7.68E-04	6.36E-03
TraesCS4D02G062900	-1.6327	0.0744	7.68E-04	6.36E-03
TraesCS7B02G046300	-1.3657	1.9329	7.70E-04	6.37E-03

TraesCS6A02G301800	-1.1885	1.222	7.86E-04	6.49E-03
TraesCS4D02G201800	1.134	5.7174	7.89E-04	6.51E-03
TraesCS1D02G372800	1.1311	1.3419	7.94E-04	6.54E-03
TraesCS4D02G149900	1.2196	1.0799	7.99E-04	6.58E-03
TraesCS3A02G149300	1.1337	1.5193	8.00E-04	6.58E-03
TraesCS6A02G265300	-1.2565	1.0299	8.13E-04	6.67E-03
TraesCS5A02G270000	1.3187	0.6721	8.19E-04	6.71E-03
TraesCS2D02G028100	-1.8249	-0.257	8.21E-04	6.72E-03
TraesCS1A02G085300	-1.1464	0.9348	8.22E-04	6.73E-03
TraesCS5B02G064800	-2.3039	-0.4415	8.22E-04	6.73E-03
TraesCS4D02G315300	1.0938	2.4966	8.25E-04	6.75E-03
TraesCS3D02G257600	1.0927	1.5808	8.26E-04	6.75E-03
TraesCS4D02G192800	-1.1914	0.9597	8.27E-04	6.76E-03
TraesCS2D02G157300	1.2938	0.6575	8.31E-04	6.79E-03
TraesCS7B02G149100	1.8927	-0.257	8.35E-04	6.82E-03
ENSRNA050024305	-1.5845	1.048	8.38E-04	6.83E-03
TraesCS4A02G023300	-1.4198	0.3486	8.49E-04	6.92E-03
TraesCS5D02G038900	-1.2456	0.5583	8.55E-04	6.96E-03
TraesCS1D02G407600	-1.2251	0.9843	8.57E-04	6.97E-03
TraesCS7A02G358300	-1.1804	1.1098	8.61E-04	7.00E-03
TraesCS7D02G159200	1.5902	0.4103	8.71E-04	7.06E-03
TraesCS4D02G302200	-1.0526	1.4615	8.73E-04	7.08E-03
ENSRNA050022736	-1.278	2.7812	8.75E-04	7.09E-03
TraesCS1A02G219200	-1.0484	1.1009	8.75E-04	7.09E-03
TraesCS2B02G324800	-1.1097	1.295	8.87E-04	7.17E-03
TraesCS3D02G113300	1.6553	0.6775	8.91E-04	7.20E-03
ENSRNA050021228	-2.1548	-0.3467	9.04E-04	7.29E-03
TraesCS2A02G211200	1.2206	0.609	9.06E-04	7.30E-03
TraesCS2D02G021800	-1.846	-0.1167	9.07E-04	7.31E-03
TraesCS4B02G239400	1.2082	2.669	9.11E-04	7.33E-03
TraesCS2A02G211400	1.3708	0.5958	9.17E-04	7.37E-03
ENSRNA050018932	-1.0424	2.0783	9.20E-04	7.38E-03
TraesCS6B02G292100	1.2957	0.9864	9.21E-04	7.39E-03
TraesCS5D02G258600	-1.3643	0.3843	9.24E-04	7.41E-03
TraesCS7D02G099900	-1.1036	1.236	9.25E-04	7.41E-03
TraesCS3D02G396100	-1.4621	0.441	9.25E-04	7.41E-03
TraesCS3B02G151300	-1.2369	0.6055	9.30E-04	7.45E-03
TraesCS7B02G216700	1.3378	0.6291	9.31E-04	7.45E-03
TraesCS4B02G189600	-1.681	0.0043	9.35E-04	7.48E-03
TraesCS2D02G377700	-1.3424	0.9185	9.44E-04	7.54E-03
TraesCS4A02G127900	-1.6471	-0.0177	9.51E-04	7.59E-03
TraesCS5A02G053000	-1.3668	0.6409	9.57E-04	7.63E-03
TraesCS7A02G123100	-1.1016	1.0672	9.72E-04	7.73E-03
TraesCS5A02G146200	-1.1566	0.7562	9.72E-04	7.73E-03
TraesCS6D02G015900	1.3509	0.4589	9.74E-04	7.74E-03
ENSRNA050018659	-1.8114	1.1641	9.74E-04	7.74E-03
TraesCS4B02G022400	-1.8031	-0.0157	9.79E-04	7.77E-03
TraesCS7A02G072900	1.8972	-0.2539	9.82E-04	7.79E-03

TraesCS6B02G143000	1.3134	0.5557	9.88E-04	7.83E-03
TraesCS5A02G141200	1.148	1.3155	1.00E-03	7.91E-03
TraesCS5B02G150200	1.7416	0.005	1.02E-03	8.06E-03
ENSRNA050024577	-1.7682	0.7256	1.02E-03	8.06E-03
TraesCS2B02G043900	1.0234	1.1091	1.02E-03	8.06E-03
TraesCS1A02G068400	1.1868	1.5106	1.05E-03	8.24E-03
TraesCS5D02G030400	-1.5287	0.0987	1.06E-03	8.31E-03
TraesCS2B02G286200	-1.2468	0.8163	1.07E-03	8.38E-03
TraesCS7D02G383500	1.1148	0.9103	1.08E-03	8.43E-03
TraesCS6D02G130600	1.5442	0.653	1.08E-03	8.45E-03
TraesCS1D02G056200	1.0794	1.1043	1.08E-03	8.45E-03
TraesCS4D02G116200	-1.3271	0.8709	1.10E-03	8.55E-03
TraesCS5B02G133600	-1.5616	0.0324	1.10E-03	8.57E-03
TraesCS5B02G237900	-1.6541	0.1838	1.10E-03	8.58E-03
TraesCS1D02G142400	-1.0657	1.236	1.12E-03	8.69E-03
TraesCS7D02G142100	-1.1662	0.8933	1.12E-03	8.72E-03
TraesCS2B02G338400	-1.2948	0.4736	1.14E-03	8.83E-03
TraesCS5B02G262600	-1.2671	1.294	1.16E-03	8.94E-03
TraesCS1B02G258400	1.7168	-0.0168	1.16E-03	8.96E-03
TraesCS1D02G119100	-1.5566	0.1201	1.16E-03	8.96E-03
TraesCS5A02G233700	-1.0556	1.5006	1.18E-03	9.07E-03
TraesCS5A02G127900	-1.359	0.7281	1.19E-03	9.16E-03
TraesCS3D02G216100	-1.4991	0.4081	1.20E-03	9.24E-03
TraesCS3B02G059500	1.3967	1.0108	1.21E-03	9.25E-03
TraesCS5D02G174100	-1.0164	1.3013	1.21E-03	9.26E-03
TraesCS4D02G005600	-1.18	1.2704	1.21E-03	9.26E-03
TraesCS6D02G361900	-1.9052	0.2692	1.21E-03	9.30E-03
TraesCS5A02G252200	-1.0912	1.2839	1.23E-03	9.38E-03
TraesCS4D02G185400	1.0017	7.0517	1.23E-03	9.39E-03
TraesCS3B02G331000	-1.5645	0.2114	1.23E-03	9.40E-03
TraesCS6B02G054600	-1.0902	1.2235	1.23E-03	9.42E-03
TraesCS4D02G217700	-1.9575	-0.3175	1.24E-03	9.43E-03
TraesCS5A02G070400	-1.7714	0.0567	1.25E-03	9.50E-03
TraesCS1A02G050800	-1.3925	0.1888	1.25E-03	9.52E-03
TraesCS1A02G220000	-1.2958	0.7833	1.27E-03	9.66E-03
TraesCS4D02G239400	1.3759	3.2155	1.27E-03	9.68E-03
TraesCS5B02G139500	1.0334	1.4173	1.28E-03	9.74E-03
TraesCS7A02G148800	-1.2466	0.5569	1.29E-03	9.79E-03
TraesCS5B02G176400	-1.1959	0.8784	1.29E-03	9.80E-03
TraesCS5B02G035700	1.7171	-0.12	1.29E-03	9.81E-03
TraesCS1B02G296700	1.0962	1.1518	1.30E-03	9.83E-03
TraesCS6B02G154000	-1.0265	1.158	1.30E-03	9.87E-03
TraesCS4B02G033600	2.039	-0.4707	1.31E-03	9.92E-03
TraesCS7A02G307500	-1.0585	1.6684	1.32E-03	9.95E-03
TraesCS5B02G214100	1.5221	0.1439	1.32E-03	9.95E-03
TraesCS4A02G166000	-1.3489	0.5104	1.32E-03	9.99E-03
TraesCS6A02G130100	-1.1046	0.9464	1.32E-03	9.99E-03
TraesCS3A02G191500	-1.1537	0.7984	1.33E-03	1.00E-02

TraesCS6A02G269600	-1.1626	1.0206	1.35E-03	1.02E-02
TraesCS4D02G033800	-2.2051	-0.5101	1.37E-03	1.03E-02
TraesCS4D02G309700	-1.0244	1.452	1.38E-03	1.03E-02
TraesCS5A02G304700	-1.1464	0.8441	1.38E-03	1.03E-02
TraesCS5D02G502900	1.0925	7.2311	1.39E-03	1.04E-02
TraesCS3D02G312100	1.2105	0.9231	1.39E-03	1.04E-02
TraesCS7D02G320400	-1.1284	1.1916	1.40E-03	1.04E-02
TraesCS6B02G178500	1.4568	0.2526	1.40E-03	1.05E-02
TraesCS5D02G405300	1.0627	3.9202	1.40E-03	1.05E-02
TraesCS7D02G252400	-1.5124	0.2672	1.41E-03	1.05E-02
TraesCS7D02G159500	-1.2979	0.4748	1.42E-03	1.06E-02
TraesCS7D02G067800	1.2788	0.2911	1.43E-03	1.06E-02
TraesCS5B02G291700	1.0623	0.9943	1.44E-03	1.07E-02
TraesCS6D02G341200	-1.2579	0.5067	1.45E-03	1.07E-02
TraesCS1A02G271400	1.6776	-0.1441	1.47E-03	1.09E-02
TraesCS2B02G189700	-1.2416	0.7751	1.47E-03	1.09E-02
TraesCS7D02G276200	-1.5795	0.03	1.47E-03	1.09E-02
TraesCS6B02G168300	-1.1339	0.6998	1.48E-03	1.10E-02
TraesCS4A02G181200	-1.1959	0.6851	1.49E-03	1.10E-02
TraesCS5B02G187700	-1.7109	-0.2011	1.49E-03	1.10E-02
TraesCS5B02G076200	-1.234	1.8786	1.51E-03	1.12E-02
TraesCS7A02G273800	1.2255	0.8149	1.52E-03	1.12E-02
TraesCS4D02G241900	-1.094	1.1569	1.53E-03	1.12E-02
TraesCS6D02G283700	-1.0315	1.3161	1.53E-03	1.12E-02
TraesCS5D02G393700	-1.7113	-0.2016	1.53E-03	1.13E-02
TraesCS5B02G024700	1.3077	0.7736	1.53E-03	1.13E-02
TraesCS4D02G359200	-1.2919	0.5872	1.54E-03	1.13E-02
TraesCS5B02G048800	-1.0978	0.8112	1.55E-03	1.14E-02
TraesCS7D02G124500	-1.3196	0.5462	1.55E-03	1.14E-02
TraesCS3D02G033600	1.5873	0.0093	1.56E-03	1.14E-02
TraesCSU02G038500	-1.6609	-0.1187	1.57E-03	1.15E-02
TraesCS1D02G334100	-1.022	0.9325	1.57E-03	1.15E-02
TraesCS6D02G367100	1.1832	0.9587	1.59E-03	1.16E-02
TraesCS4A02G066100	1.4972	0.4383	1.59E-03	1.16E-02
TraesCS6A02G267800	-1.7247	-0.0686	1.60E-03	1.17E-02
TraesCS1A02G136000	-1.0807	1.2395	1.61E-03	1.17E-02
TraesCS3B02G266800	-1.2974	0.695	1.61E-03	1.17E-02
TraesCS5A02G047700	-1.5316	0.1008	1.61E-03	1.17E-02
TraesCS6B02G286100	1.1621	1.4158	1.65E-03	1.20E-02
TraesCS3A02G247900	1.1215	0.9936	1.65E-03	1.20E-02
TraesCS4D02G034500	-1.4525	0.3667	1.67E-03	1.21E-02
ENSRNA050023570	-1.5896	1.1918	1.67E-03	1.21E-02
TraesCS2B02G344200	-1.0666	0.9186	1.68E-03	1.21E-02
TraesCS3A02G261500	-1.6639	-0.2286	1.71E-03	1.23E-02
TraesCS5A02G297400	1.3171	0.7118	1.72E-03	1.24E-02
ENSRNA050016673	-1.4357	0.9016	1.75E-03	1.26E-02
TraesCS7B02G057900	1.2499	0.5754	1.75E-03	1.26E-02
TraesCS1A02G132300	1.0263	0.9314	1.75E-03	1.26E-02

TraesCS2D02G210900	-1.1593	0.9851	1.77E-03	1.27E-02
TraesCS1D02G155500	-1.1404	1.322	1.78E-03	1.27E-02
ENSRNA050018939	-1.829	1.45	1.80E-03	1.28E-02
TraesCS5A02G140900	-1.2652	0.6856	1.81E-03	1.29E-02
TraesCS4D02G345300	-1.0893	0.8944	1.84E-03	1.31E-02
TraesCS6D02G026700	-1.415	0.2699	1.84E-03	1.31E-02
TraesCS3A02G235300	-1.183	0.7306	1.86E-03	1.32E-02
TraesCS5A02G211400	1.1826	0.9811	1.86E-03	1.32E-02
TraesCS5D02G153600	1.0117	1.3679	1.87E-03	1.32E-02
TraesCS5A02G311900	1.3081	1.7123	1.89E-03	1.33E-02
TraesCS6D02G315700	-1.0672	0.9911	1.90E-03	1.34E-02
TraesCS7B02G272100	1.4208	0.5642	1.91E-03	1.35E-02
TraesCS5D02G038800	1.012	1.4871	1.92E-03	1.35E-02
TraesCS4B02G244900	-1.2984	0.3456	1.92E-03	1.36E-02
TraesCS7A02G240400	-1.6107	-0.1438	1.95E-03	1.37E-02
TraesCS6B02G185300	-1.2788	0.5254	1.96E-03	1.38E-02
TraesCS5D02G103800	-1.0744	1.4226	1.96E-03	1.38E-02
ENSRNA050024365	-1.4086	0.7815	1.97E-03	1.38E-02
TraesCS3A02G177300	-1.2658	1.3635	1.98E-03	1.39E-02
TraesCS7B02G022200	1.1792	0.6891	2.00E-03	1.40E-02
TraesCS5D02G076300	1.1404	1.0426	2.02E-03	1.41E-02
TraesCS3D02G312400	-1.052	1.7084	2.02E-03	1.41E-02
ENSRNA050016685	-1.5941	2.606	2.03E-03	1.42E-02
TraesCS1B02G292600	-1.0007	1.6332	2.03E-03	1.42E-02
ENSRNA050020639	-1.2397	2.1439	2.04E-03	1.42E-02
TraesCS3D02G193600	1.0903	2.0809	2.04E-03	1.42E-02
TraesCS6A02G106000	-1.0657	1.1813	2.05E-03	1.43E-02
TraesCS7A02G169000	1.735	0.0038	2.06E-03	1.44E-02
TraesCS1D02G214900	-1.2894	0.4	2.07E-03	1.44E-02
TraesCS3B02G277300	1.033	0.898	2.07E-03	1.44E-02
TraesCS1A02G077800	-1.1341	0.7825	2.08E-03	1.45E-02
TraesCS2B02G164100	-1.1976	0.7278	2.09E-03	1.45E-02
TraesCS6D02G125500	-1.2852	0.4006	2.10E-03	1.46E-02
TraesCS4D02G238500	-1.0247	0.9686	2.10E-03	1.46E-02
TraesCSU02G242000	-1.7461	0.4198	2.11E-03	1.46E-02
TraesCSU02G257600	1.6358	-0.1691	2.13E-03	1.47E-02
TraesCS5D02G005700	-1.7867	-0.1453	2.13E-03	1.48E-02
TraesCS1A02G232600	-1.8145	-0.0192	2.18E-03	1.50E-02
TraesCS4B02G052000	1.226	0.5604	2.20E-03	1.51E-02
TraesCS1D02G372400	1.0231	0.8544	2.20E-03	1.51E-02
TraesCS1D02G394400	-1.7292	-0.3148	2.22E-03	1.52E-02
TraesCS7B02G296200	1.2748	0.3493	2.22E-03	1.52E-02
TraesCS5B02G054900	-1.4776	0.5114	2.24E-03	1.53E-02
TraesCS1B02G116000	-1.5735	0.1243	2.25E-03	1.54E-02
TraesCS7D02G408500	-1.1165	0.6373	2.25E-03	1.54E-02
TraesCS5A02G231100	-1.5973	0.1221	2.26E-03	1.54E-02
TraesCS3A02G180700	-1.0448	0.942	2.27E-03	1.55E-02
TraesCS5A02G292000	1.5861	-0.0939	2.28E-03	1.55E-02

TraesCS6B02G088900	1.1242	3.5122	2.29E-03	1.56E-02
TraesCS1A02G302300	-1.1971	0.6841	2.32E-03	1.57E-02
TraesCS6A02G090200	-1.2315	0.4882	2.32E-03	1.58E-02
ENSRNA050013875	-1.4344	0.365	2.32E-03	1.58E-02
TraesCS1A02G187100	-1.4485	0.0524	2.35E-03	1.59E-02
TraesCS1A02G334700	-1.4851	0.0764	2.35E-03	1.60E-02
TraesCS7D02G346800	-1.4959	0.325	2.36E-03	1.60E-02
TraesCS5D02G015200	1.0714	0.7567	2.39E-03	1.62E-02
TraesCS3B02G314800	-1.5107	0.1866	2.40E-03	1.62E-02
TraesCSU02G003600	1.4659	0.1886	2.43E-03	1.64E-02
TraesCS5D02G236700	-1.7816	-0.283	2.43E-03	1.64E-02
TraesCS3A02G056800	-1.1907	2.7035	2.45E-03	1.65E-02
TraesCS2B02G094500	-1.6359	-0.0194	2.46E-03	1.66E-02
TraesCS5D02G373600	-1.4815	0.2492	2.46E-03	1.66E-02
TraesCS5D02G481000	1.0771	0.7174	2.46E-03	1.66E-02
TraesCS6D02G061600	1.0281	0.8586	2.47E-03	1.66E-02
ENSRNA050019700	-1.7056	2.2837	2.47E-03	1.66E-02
TraesCS7A02G176700	1.0215	2.0612	2.47E-03	1.66E-02
TraesCS1D02G157000	-1.6158	-0.1439	2.49E-03	1.67E-02
TraesCS2D02G080500	-1.4036	0.1886	2.50E-03	1.68E-02
TraesCS2D02G112000	1.393	8.5714	2.50E-03	1.68E-02
TraesCS4D02G210200	1.1853	0.8767	2.50E-03	1.68E-02
TraesCS5D02G414100	-1.6603	-0.2287	2.51E-03	1.68E-02
TraesCS5A02G238400	1.395	0.1486	2.52E-03	1.69E-02
TraesCS6B02G213300	-1.448	0.0514	2.55E-03	1.70E-02
TraesCS4B02G125200	1.1139	1.3155	2.58E-03	1.72E-02
ENSRNA050021430	-1.3386	1.8851	2.58E-03	1.72E-02
TraesCS3A02G130900	1.279	0.2935	2.61E-03	1.74E-02
TraesCS2D02G033300	1.1635	0.6279	2.61E-03	1.74E-02
TraesCS5D02G143200	-1.0488	1.0628	2.62E-03	1.74E-02
TraesCS4A02G202600	-1.0482	1.2424	2.62E-03	1.74E-02
TraesCS2D02G315800	1.1432	1.4782	2.63E-03	1.75E-02
TraesCS1D02G275300	-1.0733	0.7964	2.65E-03	1.76E-02
TraesCS1D02G146300	1.2152	0.4415	2.65E-03	1.76E-02
TraesCS4A02G048300	1.14	0.8459	2.66E-03	1.76E-02
TraesCS2B02G367000	-1.3315	0.2289	2.66E-03	1.76E-02
TraesCS5B02G063400	-1.1392	0.7454	2.69E-03	1.78E-02
TraesCS1D02G278000	-1.1229	0.5927	2.70E-03	1.78E-02
TraesCS6A02G277000	1.358	2.4747	2.70E-03	1.79E-02
TraesCS4A02G004400	-1.6704	-0.2269	2.71E-03	1.79E-02
TraesCS7A02G300900	1.1723	0.862	2.71E-03	1.79E-02
TraesCS7D02G206000	-1.6594	-0.2285	2.77E-03	1.82E-02
TraesCS6D02G355900	-1.4733	-0.1182	2.79E-03	1.83E-02
TraesCS2A02G001300	1.0941	1.1261	2.79E-03	1.83E-02
TraesCS1D02G029700	-1.474	-0.1196	2.80E-03	1.84E-02
TraesCS1D02G144500	1.2339	0.5146	2.82E-03	1.85E-02
TraesCS5B02G277500	-1.3435	0.0773	2.86E-03	1.86E-02
TraesCS1B02G082300	1.1981	0.3685	2.89E-03	1.88E-02

TraesCS6A02G237700	-1.4538	0.0535	2.90E-03	1.89E-02
TraesCS7B02G051200	1.2314	0.5087	2.93E-03	1.91E-02
TraesCS7D02G008500	-1.2235	0.5922	2.96E-03	1.92E-02
TraesCS7D02G206300	-1.2304	0.3663	2.96E-03	1.92E-02
TraesCS6B02G145300	1.0285	1.0741	2.98E-03	1.93E-02
TraesCS3A02G239600	-1.077	0.7584	3.00E-03	1.94E-02
TraesCS6A02G258000	-1.3321	0.4906	3.02E-03	1.95E-02
TraesCS3A02G136700	1.2207	1.3636	3.03E-03	1.96E-02
ENSRNA050024362	2.104	2.5795	3.04E-03	1.96E-02
TraesCSU02G102100	-1.1299	0.5427	3.05E-03	1.97E-02
TraesCS4D02G163900	-1.4217	0.2052	3.06E-03	1.97E-02
TraesCS2D02G067600	1.1627	0.8665	3.08E-03	1.98E-02
TraesCS5D02G450100	1.5525	-0.1169	3.08E-03	1.98E-02
TraesCS2D02G364800	1.3724	0.212	3.10E-03	1.99E-02
TraesCS7D02G210300	-1.1266	1.1364	3.10E-03	1.99E-02
TraesCS4D02G343600	-1.3742	0.4054	3.11E-03	2.00E-02
TraesCS6B02G082000	-1.263	0.3281	3.14E-03	2.02E-02
TraesCS2B02G049100	-1.3018	0.2062	3.15E-03	2.02E-02
TraesCS5D02G183200	-1.2349	0.3106	3.16E-03	2.02E-02
ENSRNA050024372	-1.8125	1.7834	3.23E-03	2.06E-02
TraesCS3D02G019900	-1.2034	0.5208	3.23E-03	2.06E-02
TraesCS7D02G346900	-1.4836	-0.0196	3.24E-03	2.07E-02
TraesCS5D02G386700	1.0272	0.8174	3.24E-03	2.07E-02
TraesCS1A02G328800	1.2827	0.4877	3.24E-03	2.07E-02
TraesCS7B02G267900	-1.4819	-0.0191	3.25E-03	2.07E-02
TraesCS3D02G235700	-1.0294	0.8569	3.25E-03	2.07E-02
TraesCS6A02G187400	1.521	0.1233	3.26E-03	2.07E-02
TraesCS2D02G256600	-1.6574	-0.2297	3.26E-03	2.07E-02
TraesCS4B02G095000	-1.41	0.0293	3.27E-03	2.08E-02
TraesCS1B02G301900	-1.2236	0.3658	3.27E-03	2.08E-02
TraesCS2B02G268100	-1.5139	-0.2003	3.28E-03	2.09E-02
TraesCSU02G026500	-1.5511	0.2736	3.29E-03	2.09E-02
TraesCS4B02G245900	-1.5255	-0.0925	3.29E-03	2.09E-02
TraesCS5D02G395600	-1.3395	0.2276	3.29E-03	2.09E-02
TraesCS6D02G327600	-1.0171	1.6165	3.30E-03	2.09E-02
TraesCS2D02G181200	1.0079	3.5732	3.30E-03	2.10E-02
ENSRNA050021251	-1.3425	0.926	3.32E-03	2.11E-02
TraesCS3A02G252600	1.2887	0.2315	3.32E-03	2.11E-02
TraesCS4D02G213300	1.0289	0.9362	3.34E-03	2.12E-02
TraesCS6B02G228300	1.3549	0.3334	3.35E-03	2.12E-02
TraesCS7B02G101900	-1.2114	0.4207	3.37E-03	2.13E-02
TraesCS1D02G422600	1.6779	-0.2588	3.44E-03	2.16E-02
TraesCS6A02G239900	-1.5597	-0.0684	3.48E-03	2.18E-02
TraesCS6A02G239800	-1.5597	-0.0684	3.48E-03	2.18E-02
TraesCS7A02G243800	-1.132	0.7871	3.50E-03	2.19E-02
TraesCS1A02G306500	-1.054	0.9189	3.51E-03	2.20E-02
TraesCS7A02G196000	1.2838	4.2292	3.51E-03	2.20E-02
TraesCS7A02G234700	1.1332	1.0326	3.52E-03	2.21E-02

TraesCS3D02G278600	1.1894	2.3054	3.53E-03	2.21E-02
TraesCS5D02G493000	-1.0373	1.0829	3.54E-03	2.21E-02
TraesCS5D02G222300	1.0126	6.2546	3.54E-03	2.21E-02
TraesCS1B02G276300	-2.1608	-0.3742	3.54E-03	2.21E-02
TraesCS1D02G395100	1.1843	0.4237	3.55E-03	2.22E-02
TraesCS2B02G056700	-1.3662	0.3294	3.55E-03	2.22E-02
TraesCS6B02G028400	1.7628	-0.2007	3.56E-03	2.22E-02
TraesCS4B02G217400	-1.4145	0.029	3.58E-03	2.23E-02
TraesCSU02G208200	1.3949	0.5696	3.59E-03	2.24E-02
TraesCS1D02G014400	-1.3041	0.2055	3.60E-03	2.25E-02
TraesCS2B02G042200	-1.4449	-0.0415	3.63E-03	2.26E-02
TraesCS2D02G093800	1.2334	1.0539	3.68E-03	2.29E-02
TraesCS5B02G277800	-1.3807	0.0976	3.70E-03	2.29E-02
TraesCS3A02G044700	1.1415	0.5103	3.71E-03	2.30E-02
TraesCS1D02G197200	-1.6169	-0.2582	3.71E-03	2.30E-02
ENSRNA050024580	-1.619	1.5691	3.75E-03	2.32E-02
TraesCS4A02G014600	1.2267	0.7183	3.79E-03	2.34E-02
TraesCS1D02G073000	1.5848	-0.1974	3.83E-03	2.36E-02
TraesCS3A02G009800	-1.167	0.5084	3.85E-03	2.37E-02
TraesCS2D02G334000	1.1613	0.8659	3.85E-03	2.37E-02
TraesCS6D02G129400	-1.3456	0.3064	3.90E-03	2.39E-02
TraesCS6B02G138800	1.1819	0.4729	3.95E-03	2.42E-02
TraesCS3B02G055900	-1.0331	0.6827	3.96E-03	2.42E-02
TraesCS3D02G344800	1.0811	0.717	4.00E-03	2.44E-02
TraesCS5B02G331800	1.4918	0.1227	4.02E-03	2.45E-02
TraesCS4D02G091800	-1.0998	0.6233	4.02E-03	2.45E-02
TraesCS4B02G194400	-1.0336	0.728	4.03E-03	2.46E-02
TraesCS7A02G140500	1.0825	0.8039	4.10E-03	2.49E-02
TraesCS2B02G336700	-1.0666	0.7008	4.10E-03	2.50E-02
TraesCS1D02G237100	1.0278	0.8188	4.12E-03	2.50E-02
TraesCS6D02G333800	-1.0012	1.2477	4.13E-03	2.51E-02
TraesCS4D02G308400	1.1675	0.7717	4.19E-03	2.54E-02
TraesCSU02G262400	-1.6295	0.6347	4.20E-03	2.54E-02
TraesCS6B02G106000	-1.2432	0.6556	4.32E-03	2.60E-02
TraesCS5D02G015300	1.0478	0.6984	4.34E-03	2.61E-02
TraesCS7A02G167600	-1.099	0.526	4.36E-03	2.62E-02
ENSRNA050016549	-1.286	3.9293	4.38E-03	2.63E-02
TraesCS5D02G014600	-1.3196	0.1421	4.40E-03	2.64E-02
ENSRNA050013834	-1.5212	0.0067	4.40E-03	2.64E-02
TraesCS1B02G156300	-1.0552	1.1197	4.41E-03	2.65E-02
TraesCS2D02G396100	-1.1652	0.6714	4.45E-03	2.67E-02
TraesCS2A02G265700	1.1178	0.8748	4.48E-03	2.68E-02
TraesCS3B02G264600	-1.2046	0.4749	4.49E-03	2.68E-02
TraesCS2A02G295200	-1.2896	0.3476	4.51E-03	2.69E-02
TraesCS6A02G237200	-1.039	0.9964	4.51E-03	2.69E-02
TraesCS6D02G115400	1.4675	-0.0657	4.51E-03	2.70E-02
TraesCS2B02G338800	-1.16	0.7691	4.51E-03	2.70E-02
TraesCS2A02G009000	1.2767	1.2839	4.55E-03	2.71E-02

TraesCS1D02G107100	-1.4055	0.0284	4.56E-03	2.72E-02
TraesCS6D02G099200	1.1256	1.1118	4.57E-03	2.73E-02
TraesCS3D02G216400	-2.0141	-0.4366	4.58E-03	2.73E-02
TraesCS3D02G280200	-1.6816	-0.3433	4.59E-03	2.73E-02
TraesCS7A02G118600	1.217	0.3873	4.61E-03	2.74E-02
TraesCS5B02G307600	1.0953	0.686	4.64E-03	2.76E-02
TraesCSU02G227200	-1.592	0.2347	4.67E-03	2.77E-02
TraesCS6B02G117900	-1.5971	0.5551	4.70E-03	2.79E-02
TraesCS4A02G197000	-1.0073	0.6699	4.72E-03	2.80E-02
TraesCS2A02G260800	1.12	0.4429	4.72E-03	2.80E-02
TraesCS3D02G119400	-1.0019	0.9225	4.77E-03	2.82E-02
TraesCS5B02G347000	-1.3243	-0.0161	4.78E-03	2.82E-02
TraesCS1A02G056100	-1.1676	0.4578	4.81E-03	2.84E-02
TraesCS1A02G319500	1.1007	2.3705	4.89E-03	2.87E-02
TraesCS6A02G110500	1.0955	0.7282	4.89E-03	2.88E-02
TraesCS7D02G355600	-1.4384	0.058	4.90E-03	2.88E-02
TraesCS5B02G165900	1.338	0.2545	4.91E-03	2.89E-02
TraesCS4B02G025000	-1.4374	-0.0444	4.94E-03	2.90E-02
TraesCS2B02G370100	1.4572	0.4613	4.98E-03	2.92E-02
TraesCS1B02G284900	-1.3311	0.4459	5.00E-03	2.93E-02
TraesCS3D02G382100	1.1087	0.8637	5.01E-03	2.93E-02
ENSRNA050024769	-1.4956	0.491	5.05E-03	2.95E-02
TraesCS4D02G296300	1.0257	0.6416	5.10E-03	2.97E-02
TraesCS6A02G228500	-1.3577	0.1624	5.14E-03	2.99E-02
TraesCS6B02G088800	-1.0266	0.8087	5.16E-03	3.00E-02
ENSRNA050016391	-1.6195	0.1638	5.19E-03	3.02E-02
ENSRNA050013847	-1.1561	2.4712	5.21E-03	3.02E-02
TraesCS4D02G131100	1.1996	0.311	5.22E-03	3.03E-02
TraesCS1A02G348600	-1.4729	0.0768	5.22E-03	3.03E-02
TraesCS1D02G150600	1.1765	0.2306	5.23E-03	3.03E-02
TraesCS1D02G405000	1.1959	0.7016	5.24E-03	3.03E-02
TraesCS7B02G106400	1.1195	0.4402	5.25E-03	3.04E-02
TraesCS4A02G025700	1.2812	0.1466	5.28E-03	3.05E-02
TraesCS5D02G188800	-1.0397	0.5909	5.30E-03	3.06E-02
TraesCS5B02G346900	-1.2048	0.424	5.34E-03	3.08E-02
TraesCS2D02G317200	1.4603	-0.1693	5.35E-03	3.08E-02
TraesCS4B02G225200	1.2652	0.146	5.37E-03	3.09E-02
TraesCS2A02G239400	-1.2124	0.229	5.45E-03	3.13E-02
TraesCS1A02G000400	-1.6002	1.8892	5.46E-03	3.13E-02
TraesCS6A02G205100	1.3778	0.0562	5.47E-03	3.14E-02
TraesCS1A02G209600	1.1243	4.6045	5.47E-03	3.14E-02
TraesCS3B02G135800	1.0873	0.9217	5.51E-03	3.16E-02
TraesCS1A02G000900	-1.5572	1.6354	5.52E-03	3.16E-02
TraesCS5B02G210500	-1.2347	0.3644	5.53E-03	3.17E-02
ENSRNA050018916	-2.1562	-0.0488	5.53E-03	3.17E-02
TraesCS3B02G325600	-1.0812	0.9567	5.55E-03	3.17E-02
TraesCS7D02G027400	-1.2327	0.3638	5.63E-03	3.21E-02
TraesCS2A02G259200	-1.1651	1.4186	5.63E-03	3.21E-02

TraesCS3D02G022500	-1.0268	0.8537	5.64E-03	3.21E-02
TraesCS1B02G310400	1.2655	0.5821	5.65E-03	3.22E-02
TraesCS3D02G175900	1.305	0.1671	5.71E-03	3.24E-02
TraesCS6B02G176300	-1.0288	0.7285	5.72E-03	3.25E-02
TraesCS3A02G086000	1.4113	0.0753	5.73E-03	3.25E-02
TraesCS3A02G102000	1.1696	0.291	5.74E-03	3.26E-02
TraesCS4B02G149800	1.1164	0.4385	5.76E-03	3.27E-02
TraesCS5D02G247400	1.2945	0.4818	5.77E-03	3.27E-02
TraesCS6D02G073700	-1.0085	0.7115	5.77E-03	3.27E-02
TraesCS5D02G146100	-1.4331	0.3507	5.77E-03	3.27E-02
TraesCS1B02G223100	-1.1309	0.6857	5.80E-03	3.28E-02
TraesCS1A02G352100	-1.0303	0.6823	5.81E-03	3.28E-02
TraesCS7A02G210600	-1.1092	0.4208	5.83E-03	3.29E-02
TraesCS3A02G059200	1.0685	0.8764	5.85E-03	3.30E-02
TraesCS5A02G028700	1.1995	0.3112	5.85E-03	3.30E-02
TraesCS1A02G210700	1.1821	2.0046	5.88E-03	3.32E-02
TraesCS3D02G154700	-1.1553	0.3814	5.91E-03	3.33E-02
TraesCS4B02G240300	1.0381	0.9445	5.95E-03	3.35E-02
TraesCS6A02G093900	1.13	0.9105	6.02E-03	3.38E-02
TraesCS6B02G290000	-1.1222	0.3652	6.03E-03	3.38E-02
TraesCS6D02G383900	1.4031	-0.016	6.03E-03	3.39E-02
TraesCS7A02G099700	1.0035	0.8379	6.09E-03	3.41E-02
TraesCS3D02G052400	-1.2735	0.3878	6.16E-03	3.44E-02
TraesCS2D02G423000	-1.6056	-0.2548	6.19E-03	3.46E-02
TraesCS7D02G048200	-1.1589	0.3866	6.22E-03	3.47E-02
TraesCS1D02G441800	-1.309	0.0565	6.24E-03	3.47E-02
TraesCSU02G026700	-1.0394	0.7898	6.25E-03	3.48E-02
TraesCS6B02G286200	-1.4109	0.1894	6.32E-03	3.51E-02
TraesCS1A02G040700	-1.1093	0.4708	6.34E-03	3.52E-02
TraesCS5B02G127000	-1.4254	-0.1452	6.44E-03	3.56E-02
TraesCS3D02G397200	-1.1749	1.0178	6.47E-03	3.58E-02
ENSRNA050021712	1.3787	-0.1204	6.48E-03	3.58E-02
TraesCS5A02G287000	-1.2309	0.1659	6.49E-03	3.59E-02
TraesCS7B02G162100	1.1478	0.4602	6.51E-03	3.60E-02
TraesCS2A02G043500	-1.0886	0.8127	6.54E-03	3.61E-02
TraesCS1A02G236300	-1.4674	0.0567	6.57E-03	3.62E-02
TraesCSU02G114300	-1.2272	0.1646	6.58E-03	3.63E-02
TraesCS5B02G183200	-1.3935	-0.0673	6.59E-03	3.63E-02
TraesCS6A02G135500	1.3133	0.0981	6.59E-03	3.63E-02
TraesCS1B02G142700	1.3606	-0.0387	6.68E-03	3.67E-02
TraesCS1A02G109100	1.2771	0.0769	6.71E-03	3.68E-02
ENSRNA050013873	-1.9182	-0.3479	6.71E-03	3.68E-02
TraesCS7D02G155700	1.0902	0.6813	6.71E-03	3.68E-02
TraesCS5D02G432600	1.1652	0.2908	6.76E-03	3.70E-02
TraesCS7D02G214700	1.1592	0.4609	6.87E-03	3.75E-02
TraesCS5D02G232300	1.1215	0.3878	6.90E-03	3.76E-02
TraesCS7A02G030600	-1.5091	-0.3124	6.91E-03	3.77E-02
TraesCS5B02G258700	1.0138	1.2528	6.96E-03	3.79E-02

TraesCS2A02G157700	-1.0576	0.6077	6.97E-03	3.79E-02
TraesCS1B02G292800	-1.0691	0.561	6.98E-03	3.80E-02
TraesCS7D02G026800	-1.0597	0.6564	7.01E-03	3.81E-02
TraesCS1A02G242000	-1.0468	0.6815	7.02E-03	3.81E-02
TraesCS5A02G236000	1.0297	0.543	7.03E-03	3.82E-02
TraesCS1A02G207200	-1.8178	0.2065	7.03E-03	3.82E-02
ENSRNA050020225	-1.7877	-0.1457	7.05E-03	3.82E-02
TraesCS5D02G291000	1.5146	0.2301	7.07E-03	3.83E-02
TraesCS6D02G308000	1.1568	0.524	7.09E-03	3.84E-02
TraesCS2A02G031100	1.352	-0.042	7.11E-03	3.84E-02
TraesCS7D02G179100	1.4348	0.0109	7.17E-03	3.87E-02
TraesCS1D02G199800	-1.1567	0.6515	7.23E-03	3.90E-02
TraesCS6D02G250700	-1.2965	0.5852	7.25E-03	3.91E-02
TraesCS6B02G270500	-1.1253	0.3088	7.27E-03	3.91E-02
ENSRNA050024755	-1.2636	3.3717	7.27E-03	3.91E-02
TraesCS3B02G079400	-1.0838	0.4022	7.28E-03	3.92E-02
TraesCS2B02G160100	1.4685	-0.168	7.29E-03	3.92E-02
TraesCS6D02G142100	-1.232	0.1626	7.33E-03	3.93E-02
TraesCS5B02G052300	-1.2859	0.5223	7.34E-03	3.94E-02
TraesCS2B02G259200	-1.8563	-0.3811	7.39E-03	3.96E-02
TraesCS5A02G036300	-1.4728	-0.1193	7.42E-03	3.97E-02
TraesCS2A02G182700	-1.438	0.1395	7.44E-03	3.98E-02
TraesCS5A02G137300	1.2232	0.1861	7.48E-03	4.00E-02
TraesCS5B02G137400	1.3543	-0.0396	7.52E-03	4.02E-02
TraesCS5A02G003600	-1.0413	0.5948	7.58E-03	4.04E-02
TraesCS3D02G316100	1.3317	0.7811	7.62E-03	4.06E-02
ENSRNA050019711	-1.7819	0.1821	7.64E-03	4.07E-02
TraesCS3A02G243300	-1.0582	0.8713	7.65E-03	4.07E-02
TraesCS7A02G078900	1.2268	0.1227	7.68E-03	4.08E-02
TraesCS4D02G141500	1.0069	2.0029	7.70E-03	4.09E-02
TraesCS1D02G056500	-1.2083	0.0777	7.78E-03	4.12E-02
TraesCS1D02G385400	1.041	2.0814	7.82E-03	4.14E-02
TraesCS5B02G236000	1.2114	0.1872	7.90E-03	4.18E-02
TraesCS5D02G401600	-1.1414	0.2491	7.94E-03	4.19E-02
TraesCS7D02G180800	-1.119	0.3657	7.95E-03	4.19E-02
TraesCS1D02G320100	-1.0823	0.5593	7.96E-03	4.20E-02
TraesCS4D02G063300	-1.1186	1.1054	8.09E-03	4.25E-02
TraesCS5B02G074100	-1.134	0.2512	8.28E-03	4.33E-02
TraesCS7D02G120800	-1.4301	-0.0397	8.32E-03	4.35E-02
TraesCS3D02G337400	-1.1098	0.5254	8.35E-03	4.36E-02
TraesCS3B02G164000	1.0039	0.7936	8.37E-03	4.37E-02
TraesCS2B02G151500	-1.3005	0.2118	8.39E-03	4.37E-02
TraesCS6B02G169500	1.5291	-0.2295	8.39E-03	4.37E-02
TraesCS5B02G221000	-1.4173	-0.254	8.41E-03	4.38E-02
TraesCS5A02G287800	1.0246	0.5444	8.41E-03	4.38E-02
TraesCS7B02G169300	-1.0094	0.6249	8.46E-03	4.40E-02
TraesCS3A02G215500	1.0965	0.3101	8.54E-03	4.44E-02
ENSRNA050024361	-1.788	0.6765	8.57E-03	4.45E-02

TraesCS5B02G005300	1.1473	0.2736	8.66E-03	4.49E-02
TraesCS6D02G393700	1.0849	0.4231	8.67E-03	4.49E-02
TraesCSU02G264900	-2.0364	-0.1253	8.72E-03	4.51E-02
TraesCS7D02G359600	-1.0764	0.458	8.74E-03	4.52E-02
TraesCS4B02G209400	1.053	0.6992	8.75E-03	4.52E-02
ENSRNA050022790	-1.0157	2.7055	8.76E-03	4.53E-02
TraesCS2B02G205900	-1.2249	0.0067	8.77E-03	4.53E-02
TraesCS2B02G012800	1.4171	1.8272	8.78E-03	4.53E-02
TraesCS5B02G263800	-1.3164	1.4884	8.81E-03	4.55E-02
TraesCS3B02G011800	1.2417	0.0572	8.86E-03	4.57E-02
ENSRNA050016389	-1.7053	0.1394	8.86E-03	4.57E-02
TraesCS3A02G154200	1.057	0.6082	8.87E-03	4.57E-02
TraesCS7A02G171600	-1.2081	0.0761	8.90E-03	4.58E-02
TraesCS4A02G159000	-1.0773	0.5081	8.92E-03	4.59E-02
TraesCS7B02G154400	-1.3023	0.0502	8.93E-03	4.59E-02
TraesCS5B02G226000	-1.4721	-0.0126	9.00E-03	4.62E-02
TraesCS5B02G264500	1.3773	0.1276	9.01E-03	4.62E-02
TraesCS6D02G098000	-1.267	0.029	9.03E-03	4.63E-02
TraesCS2A02G068900	1.1803	0.1673	9.05E-03	4.64E-02
TraesCS7D02G386900	-1.0477	0.4375	9.07E-03	4.65E-02
TraesCS5D02G362500	1.0487	1.4348	9.13E-03	4.67E-02
TraesCS1B02G166000	-1.1053	0.287	9.14E-03	4.68E-02
TraesCS1B02G174200	-1.3494	-0.095	9.17E-03	4.68E-02
TraesCSU02G215400	-1.0103	0.5739	9.22E-03	4.70E-02
TraesCS5D02G230100	-1.087	0.4065	9.27E-03	4.72E-02
TraesCS1D02G289000	-1.2472	0.5131	9.30E-03	4.73E-02
TraesCSU02G246100	1.2895	0.6423	9.39E-03	4.77E-02
ENSRNA050019323	-1.3893	1.0504	9.43E-03	4.79E-02
TraesCS1A02G000300	-1.4404	0.1464	9.61E-03	4.86E-02
TraesCS1D02G265000	-1.2995	0.0514	9.67E-03	4.88E-02
TraesCS2A02G200800	1.0503	0.9838	9.69E-03	4.89E-02
TraesCS2B02G313800	-1.0968	0.7143	9.70E-03	4.89E-02
ENSRNA050019988	-1.1294	0.7379	9.76E-03	4.91E-02
TraesCS6D02G075800	-1.1348	0.25	9.80E-03	4.93E-02
TraesCS3B02G054000	-1.0009	0.7159	9.92E-03	4.98E-02

Supplementary Table S3 Comparison of foldchange between results of qRT-PCR and RNA-seq

	gene name	qRT-PCR		Foldchange (S1/S0)	RNA-seq		Foldchange	R2
		S0	S1		S0	S1		
For DEGs validation	TraesCS1D02G399000	5.78	8.15	1.40	3.68	9.35	2.540357	1
	TraesCS1A02G149300	27.18	15.64	0.57	37.77	18.74	0.496293	1
	TraesCS6B02G201600	14.57	21.88	1.50	11.87	24.34	2.051693	1
	TraesCS6B02G286200	0.49	0.25	0.51	0.72	0.34	0.47907	1
	TraesCS7A02G333900	102.03	64.12	0.62	87.25	56.88	0.651847	1
	TraesCS7D02G341500	86.74	50.93	0.58	75.12	45.87	0.610638	1
	TraesCS6A02G173700	57.29	44.12	0.77	67.74	42.65	0.629536	1
	TraesCS1D02G176400	21.11	17.00	0.80	31.33	13.90	0.443837	1
	TraesCS3A02G270100	6.60	5.56	0.84	11.65	7.24	0.621981	1
	TraesCS1B02G167200	30.22	18.46	0.61	25.28	11.65	0.460786	1
	TraesCS1A02G177800	13.51	8.35	0.61	11.35	9.53	0.840375	1
	TraesCS2D02G354300	5.43	8.69	1.60	11.46	15.96	1.392767	1
For MYB	TraesCS1B02G100600	7.79	3.34	0.42	11.41	5.42	0.475329	1
	TraesCS2B02G177900	19.47	8.90	0.45	23.24	11.40	0.490331	1
	TraesCS2D02G158200	13.55	7.99	0.58	17.57	10.35	0.588974	1
	TraesCS3B02G224500	4.59	1.24	0.26	8.68	3.74	0.430569	1
	TraesCS3B02G286500	5.88	3.10	0.52	9.86	5.60	0.567363	1
	TraesCS5A02G142500	1.87	0.68	0.36	5.36	3.18	0.593046	1
	TraesCS5B02G119300	1.65	0.66	0.39	5.55	3.16	0.569222	1
	TraesCS6A02G265300	3.02	0.79	0.25	7.35	3.29	0.446675	1
	TraesCS6D02G298200	1.53	0.68	0.44	5.33	3.18	0.595563	1
	TraesCS7A02G165700	2.11	0.77	0.36	6.44	3.27	0.508624	1

Supplementary Table S4 Differentially expressed genes under silicon treatment

Wheat ID	TF family	Blast2At	e-value	description
TraesCS2B02G268100	AP2	AT4G37750.1	1.00E-143	AP2 family protein
TraesCS2D02G256600	AP2	AT4G37750.1	1.00E-143	AP2 family protein
TraesCS5D02G150500	AP2	AT3G54320.3	6.00E-99	AP2 family protein
TraesCS1D02G367700	bHLH	AT3G21330.1	7.00E-40	bHLH family protein
TraesCS2A02G081300	bHLH	AT3G47640.2	3.00E-31	bHLH family protein
TraesCS2A02G275600	bHLH	AT3G07340.1	7.00E-67	bHLH family protein
TraesCS2A02G281200	bHLH	AT2G28160.1	4.00E-41	FER-like regulator of iron uptake
TraesCS2B02G095900	bHLH	AT3G47640.2	2.00E-35	bHLH family protein
TraesCS2B02G293300	bHLH	AT3G07340.1	5.00E-65	bHLH family protein
TraesCS2D02G079100	bHLH	AT3G47640.2	2.00E-31	bHLH family protein
TraesCS2D02G274600	bHLH	AT3G07340.1	1.00E-66	bHLH family protein
TraesCS2D02G280100	bHLH	AT2G28160.1	3.00E-41	FER-like regulator of iron uptake
TraesCS3D02G208500	bHLH	AT3G21330.1	9.00E-46	bHLH family protein
TraesCS4B02G056600	bHLH	AT4G37850.1	4.00E-58	bHLH family protein
TraesCS4B02G217400	bHLH	AT1G68810.1	1.00E-75	bHLH family protein
TraesCS4D02G051600	bHLH	AT3G26744.1	5.00E-90	bHLH family protein
TraesCS4D02G217700	bHLH	AT1G68810.1	4.00E-77	bHLH family protein
TraesCS5A02G237500	bHLH	AT5G67060.1	9.00E-45	bHLH family protein
TraesCS5B02G235200	bHLH	AT3G50330.1	6.00E-44	bHLH family protein
TraesCS5D02G244000	bHLH	AT5G67060.1	1.00E-44	bHLH family protein
TraesCS6B02G291200	bHLH	AT1G72210.1	8.00E-72	bHLH family protein
TraesCS6D02G250700	bHLH	AT1G72210.1	6.00E-72	bHLH family protein
TraesCS6D02G285300	bHLH	AT1G26945.1	2.00E-28	bHLH family protein
TraesCS6D02G341200	bHLH	AT5G58010.1	9.00E-75	LJRHL1-like 3
TraesCS7A02G185300	bHLH	AT1G26945.1	6.00E-26	bHLH family protein
TraesCS7B02G090500	bHLH	AT1G26945.1	8.00E-26	bHLH family protein
TraesCS7B02G152800	bHLH	AT3G07340.1	4.00E-62	bHLH family protein
TraesCS7D02G187000	bHLH	AT1G26945.1	8.00E-26	bHLH family protein
TraesCS3A02G131900	bZIP	AT3G58120.1	7.00E-74	bZIP family protein
TraesCS4B02G117400	bZIP	AT1G19490.1	8.00E-27	bZIP family protein
TraesCS4D02G115200	bZIP	AT1G19490.1	8.00E-19	bZIP family protein
TraesCS6B02G209600	bZIP	AT5G11260.1	7.00E-53	bZIP family protein
TraesCS3D02G350700	C2H2	AT3G46090.1	2.00E-24	C2H2 family protein
TraesCS4A02G089900	C2H2	AT2G29660.1	6.00E-70	C2H2 family protein
TraesCS4D02G141500	C2H2	AT2G37430.1	3.00E-24	C2H2 family protein
TraesCS5A02G233700	C2H2	AT1G68130.1	1.00E-127	indeterminate(ID)-domain 14
TraesCS5D02G491000	C2H2	AT5G59820.1	6.00E-21	C2H2 family protein
TraesCS5D02G491100	C2H2	AT3G46080.1	4.00E-23	C2H2 family protein
TraesCS6B02G154000	C2H2	AT1G03840.1	1.00E-102	C2H2 family protein
TraesCS7D02G242600	C2H2	AT3G57670.1	1.00E-120	C2H2 family protein
TraesCSU02G012300	C2H2	AT1G75710.1	3.00E-94	C2H2-like zinc finger protein
TraesCS3B02G261800	C3H	AT1G66810.1	6.00E-66	C3H family protein
TraesCS3D02G220700	C3H	AT1G66810.1	8.00E-68	C3H family protein
TraesCS1A02G275000	Dof	AT3G52440.1	7.00E-37	Dof family protein
TraesCS5D02G493000	Dof	AT3G55370.2	3.00E-47	OBF-binding protein 3

TraesCS1A02G220000	ERF	AT2G44940.1	4.00E-50	ERF family protein
TraesCS1A02G328800	ERF	AT3G23240.1	8.00E-51	ethylene response factor 1
TraesCS1D02G376600	ERF	AT4G34410.1	3.00E-36	redox responsive transcription factor 1
TraesCS2D02G397000	ERF	AT4G25470.1	2.00E-49	C-repeat/DRE binding factor 2
TraesCS3B02G266800	ERF	AT4G27950.1	4.00E-33	cytokinin response factor 4
TraesCS4D02G298400	ERF	AT1G72360.3	1.00E-33	ERF family protein
TraesCS4D02G298500	ERF	AT2G47520.1	2.00E-11	ERF family protein
TraesCS5A02G238400	ERF	AT4G34410.1	2.00E-32	redox responsive transcription factor 1
TraesCS5A02G311900	ERF	AT5G51990.1	7.00E-36	C-repeat-binding factor 4
TraesCS5A02G312000	ERF	AT5G51990.1	8.00E-36	C-repeat-binding factor 4
TraesCS5B02G312700	ERF	AT5G51990.1	2.00E-41	C-repeat-binding factor 4
TraesCS5B02G312800	ERF	AT4G25470.1	3.00E-36	C-repeat/DRE binding factor 2
TraesCS5B02G312900	ERF	AT5G51990.1	4.00E-37	C-repeat-binding factor 4
TraesCS5B02G313000	ERF	AT4G25470.1	1.00E-39	C-repeat/DRE binding factor 2
TraesCS5D02G129400	ERF	AT2G33710.1	5.00E-32	ERF family protein
TraesCS5D02G180900	ERF	AT2G47520.1	2.00E-11	ERF family protein
TraesCS5D02G318800	ERF	AT4G25470.1	7.00E-37	C-repeat/DRE binding factor 2
TraesCS5D02G318900	ERF	AT4G25470.1	3.00E-39	C-repeat/DRE binding factor 2
TraesCS6A02G131200	ERF	AT3G15210.1	6.00E-30	thylene responsive element binding factor 4
TraesCS6A02G181400	ERF	AT1G15360.1	9.00E-76	ERF family protein
TraesCS6A02G256900	ERF	AT4G25470.1	1.00E-60	C-repeat/DRE binding factor 2
TraesCS6B02G159600	ERF	AT3G15210.1	3.00E-29	thylene responsive element binding factor 4
TraesCS6B02G210300	ERF	AT1G15360.1	9.00E-79	ERF family protein
TraesCS6B02G268100	ERF	AT4G25470.1	1.00E-60	C-repeat/DRE binding factor 2
TraesCS6D02G121100	ERF	AT3G15210.1	3.00E-29	thylene responsive element binding factor 4
TraesCS6D02G238200	ERF	AT4G25470.1	1.00E-61	C-repeat/DRE binding factor 2
TraesCS6D02G309600	ERF	AT1G21910.1	5.00E-38	ERF family protein
TraesCS7A02G057700	ERF	AT4G25480.1	7.00E-47	dehydration response element B1A
TraesCS7B02G162100	ERF	AT5G61890.1	3.00E-32	ERF family protein
TraesCS7B02G277800	ERF	AT1G15360.1	2.00E-82	ERF family protein
TraesCS7D02G127600	ERF	AT2G23340.1	5.00E-46	DREB and EAR motif protein 3
TraesCS1A02G221600	GATA	AT2G45050.1	9.00E-62	GATA transcription factor 2
TraesCS3B02G308500	GATA	AT5G25830.1	3.00E-75	GATA transcription factor 12
TraesCS6D02G173000	GATA	AT5G26930.1	4.00E-21	GATA transcription factor 23
TraesCS5B02G326100	GRAS	AT1G50600.1	1.00E-148	scarecrow-like 5
TraesCS6A02G269600	GRF	AT3G13960.1	1.00E-45	growth-regulating factor 5
TraesCS6D02G315700	GRF	AT3G13960.1	1.00E-56	growth-regulating factor 5
TraesCS4D02G286400	HD-ZIP	AT1G69780.1	2.00E-87	HD-ZIP family protein
TraesCS4A02G016600	HD-ZIP	AT1G69780.1	2.00E-85	HD-ZIP family protein
TraesCS1A02G219200	HD-ZIP	AT3G60390.1	1.00E-84	homeobox-leucine zipper protein 3
TraesCS4D02G303500	HD-ZIP	AT4G40060.1	8.00E-44	homeobox protein 16
TraesCS7D02G078900	HD-ZIP	AT5G06710.1	2.00E-56	homeobox from Arabidopsis thaliana
TraesCS7D02G079000	HD-ZIP	AT4G17460.1	4.00E-61	Homeobox-leucine zipper protein 4
TraesCS7A02G083800	HD-ZIP	AT4G17460.1	1.00E-60	Homeobox-leucine zipper protein 4
TraesCS6A02G120600	HD-ZIP	AT4G16780.1	4.00E-40	homeobox protein 2
TraesCS6D02G304300	HD-ZIP	AT1G73360.1	0	homeodomain GLABROUS 11
TraesCS7B02G072700	HD-ZIP	AT1G73360.1	0	homeodomain GLABROUS 11
TraesCS4A02G027700	HSF	AT3G22830.1	9.00E-97	heat shock transcription factor A6B

TraesCS5A02G237600	HSF	AT1G46264.1	2.00E-90	heat shock transcription factor B4
TraesCS5B02G236100	HSF	AT1G46264.1	3.00E-90	heat shock transcription factor B4
TraesCS7A02G360400	HSF	AT2G26150.1	5.00E-71	heat shock transcription factor A2
TraesCS7B02G267300	HSF	AT2G26150.1	9.00E-75	heat shock transcription factor A2
TraesCS2A02G194500	LBD	AT5G67420.1	1.00E-66	LOB domain-containing protein 37
TraesCS2B02G212400	LBD	AT5G67420.1	8.00E-70	LOB domain-containing protein 37
TraesCS2D02G193400	LBD	AT5G67420.1	3.00E-32	LOB domain-containing protein 37
TraesCS3D02G397200	LBD	AT1G65620.3	1.00E-59	LBD family protein
TraesCS4B02G197100	LBD	AT1G07900.1	3.00E-62	LOB domain-containing protein 1
TraesCS4D02G197400	LBD	AT1G07900.1	1.00E-62	LOB domain-containing protein 1
TraesCS7D02G261900	-type_MAI	AT2G45660.1	6.00E-28	AGAMOUS-like 20
TraesCS1A02G083100	MYB	AT1G09540.1	8.00E-90	myb domain protein 61
TraesCS1B02G100600	MYB	AT1G09540.1	2.00E-90	myb domain protein 61
TraesCS1D02G084400	MYB	AT1G09540.1	1.00E-89	myb domain protein 61
TraesCS1D02G371700	MYB	AT5G26660.1	2.00E-71	myb domain protein 86
TraesCS1D02G371800	MYB	AT5G26660.1	1.00E-71	myb domain protein 86
TraesCS1D02G387100	MYB	AT2G36890.1	2.00E-69	MYB family protein
TraesCS2B02G177900	MYB	AT4G01680.3	1.00E-71	myb domain protein 55
TraesCS2D02G158200	MYB	AT1G57560.1	2.00E-73	myb domain protein 50
TraesCS3A02G044700	MYB	AT1G68320.1	2.00E-79	myb domain protein 62
TraesCS3A02G200300	MYB	AT5G26660.1	2.00E-71	myb domain protein 86
TraesCS3B02G224500	MYB	AT5G26660.1	4.00E-70	myb domain protein 86
TraesCS3B02G286500	MYB	AT5G26660.1	7.00E-75	myb domain protein 86
TraesCS3B02G298200	MYB	AT4G37780.1	8.00E-69	myb domain protein 87
TraesCS4A02G189700	MYB	AT5G62470.2	2.00E-85	myb domain protein 96
TraesCS4D02G309700	MYB	AT4G21440.1	4.00E-57	MYB-like 102
TraesCS5A02G101000	MYB	AT3G08500.1	5.00E-72	myb domain protein 83
TraesCS5A02G142500	MYB	AT1G08810.1	2.00E-88	myb domain protein 60
TraesCS5A02G205200	MYB	AT1G66230.1	4.00E-90	myb domain protein 20
TraesCS5B02G105900	MYB	AT3G08500.1	9.00E-69	myb domain protein 83
TraesCS5B02G119300	MYB	AT4G38620.1	5.00E-89	myb domain protein 4
TraesCS6A02G236900	MYB	AT3G61250.1	4.00E-91	myb domain protein 17
TraesCS6A02G265300	MYB	AT1G16490.1	4.00E-75	myb domain protein 58
TraesCS6D02G298100	MYB	AT1G34670.1	1.00E-105	myb domain protein 93
TraesCS6D02G298200	MYB	AT1G34670.1	1.00E-106	myb domain protein 93
TraesCS7A02G030600	MYB	AT5G16770.2	2.00E-87	myb domain protein 9
TraesCS7A02G165700	MYB	AT5G49330.1	3.00E-60	myb domain protein 111
TraesCS7D02G026800	MYB	AT5G16770.2	7.00E-87	myb domain protein 9
TraesCS7D02G340300	MYB	AT1G09540.1	3.00E-78	myb domain protein 61
TraesCS2B02G094500	IYB_relate	AT1G15720.1	5.00E-11	TRF-like 5
TraesCS5B02G277800	IYB_relate	AT4G39250.1	2.00E-23	RAD-like 1
TraesCS7A02G233300	IYB_relate	AT1G75250.2	2.00E-22	RAD-like 6
TraesCS7D02G233300	IYB_relate	AT4G39250.1	1.00E-21	RAD-like 1
TraesCS1B02G274300	NAC	AT1G01720.1	1.00E-117	NAC family protein
TraesCS2D02G100600	NAC	AT5G61430.1	1.00E-80	NAC domain containing protein 100
TraesCS2D02G324700	NAC	AT5G61430.1	6.00E-95	NAC domain containing protein 100
TraesCS4B02G072400	NAC	AT3G18400.1	1.00E-95	NAC domain containing protein 58
TraesCS4B02G242600	NAC	AT1G25580.1	1.00E-34	NAC family protein

TraesCS4D02G071200	NAC	AT3G18400.1	1.00E-94	NAC domain containing protein 58
TraesCS4D02G345300	NAC	AT1G65910.1	1.00E-131	NAC domain containing protein 28
TraesCS5D02G420800	NAC	AT2G43000.1	2.00E-85	NAC domain containing protein 42
TraesCS6B02G286200	NAC	AT1G12260.1	1.00E-113	NAC 007
TraesCS6D02G362900	NAC	AT4G28530.1	2.00E-78	NAC domain containing protein 74
TraesCS7A02G068300	NAC	AT2G46770.1	1.00E-100	NAC family protein
TraesCS7A02G317100	NAC	AT2G46770.1	1.00E-124	NAC family protein
TraesCS7A02G326000	NAC	AT1G71930.1	3.00E-84	vascular related NAC-domain protein 7
TraesCS7B02G226600	NAC	AT1G71930.1	1.00E-84	vascular related NAC-domain protein 7
TraesCS7D02G008500	NAC	AT1G12260.1	1.00E-118	NAC 007
TraesCS7D02G314000	NAC	AT2G46770.1	1.00E-125	NAC family protein
TraesCS7D02G322700	NAC	AT1G71930.1	6.00E-85	vascular related NAC-domain protein 7
TraesCS3D02G052400	RAV	AT1G68840.2	1.00E-106	related to ABI3/VP1 2
TraesCSU02G227200	RAV	AT1G68840.2	1.00E-106	related to ABI3/VP1 2
TraesCS5A02G286700	SBP	AT5G50670.1	3.00E-44	SBP family protein
TraesCS6D02G142100	SBP	AT1G02065.1	3.00E-55	squamosa promoter binding protein-like 8
TraesCS7A02G358300	TALE	AT4G32980.1	5.00E-58	homeobox gene 1
TraesCS7D02G364500	TALE	AT4G32980.1	5.00E-58	homeobox gene 1
TraesCS3B02G164500	TCP	AT3G15030.2	5.00E-87	TCP family protein
TraesCS4A02G222700	TCP	AT1G35560.1	3.00E-55	TCP family protein
TraesCS5A02G121800	TCP	AT1G58100.1	2.00E-63	TCP family protein
TraesCS6D02G285600	TCP	AT3G47620.1	7.00E-59	cycloidea and PCF (TCP) 14
TraesCS2D02G388500	Trihelix	AT3G24860.1	2.00E-17	Trihelix family protein
TraesCS3D02G271000	Trihelix	AT3G58630.1	2.00E-49	Trihelix family protein
TraesCS1A02G301100	WRKY	AT3G56400.1	8.00E-28	WRKY DNA-binding protein 70
TraesCS1A02G348600	WRKY	AT5G41570.1	4.00E-60	WRKY DNA-binding protein 24
TraesCS1D02G351600	WRKY	AT5G41570.1	4.00E-59	WRKY DNA-binding protein 24
TraesCS1D02G418000	WRKY	AT4G23810.1	1.00E-29	WRKY family protein
TraesCS1D02G418600	WRKY	AT4G23810.1	1.00E-29	WRKY family protein
TraesCS2A02G182700	WRKY	AT2G03340.1	9.00E-71	WRKY DNA-binding protein 3
TraesCS2D02G390200	WRKY	AT2G44745.1	7.00E-61	WRKY family protein
TraesCS3A02G280400	WRKY	AT5G41570.1	5.00E-56	WRKY DNA-binding protein 24
TraesCS3B02G130000	WRKY	AT5G15130.1	2.00E-77	WRKY DNA-binding protein 72
TraesCS3B02G277300	WRKY	AT5G64810.1	2.00E-40	WRKY DNA-binding protein 51
TraesCS3D02G113300	WRKY	AT5G15130.1	2.00E-77	WRKY DNA-binding protein 72
TraesCS3D02G280200	WRKY	AT5G41570.1	6.00E-57	WRKY DNA-binding protein 24
TraesCS3D02G337400	WRKY	AT3G56400.1	8.00E-18	WRKY DNA-binding protein 70
TraesCS5A02G225600	WRKY	AT1G80840.1	8.00E-51	WRKY DNA-binding protein 40
TraesCS5B02G224000	WRKY	AT1G80840.1	6.00E-54	WRKY DNA-binding protein 40
TraesCS5B02G224100	WRKY	AT1G80840.1	7.00E-52	WRKY DNA-binding protein 40
TraesCS5D02G232800	WRKY	AT1G80840.1	8.00E-55	WRKY DNA-binding protein 40
TraesCS5D02G232900	WRKY	AT4G31800.2	2.00E-44	WRKY DNA-binding protein 18
TraesCS6D02G136200	WRKY	AT1G80840.1	2.00E-61	WRKY DNA-binding protein 40
TraesCS4B02G245900	YABBY	AT1G69180.1	2.00E-52	YABBY family protein
TraesCS5B02G025100	YABBY	AT2G26580.1	2.00E-62	YABBY family protein
TraesCS6A02G237700	YABBY	AT2G45190.1	2.00E-73	YABBY family protein
TraesCS4B02G050800	ZF-HD	AT4G24660.1	9.00E-48	homeobox protein 22
TraesCS5B02G226000	ZF-HD	AT3G28917.1	2.00E-27	mini zinc finger 2

TraesCS5D02G234700	ZF-HD	AT3G28917.1	1.00E-27	mini zinc finger 2
TraesCS5D02G253100	ZF-HD	AT2G02540.1	9.00E-62	homeobox protein 21

Supplementary Table S5 Identification of MYB TFs in wheat genome

Gene name	Chr	EST validation
TraesCS4D02G142900	4D	100
TraesCS4A02G172200	4A	100
TraesCS1A02G406600	1A	35
TraesCS1D02G415000	1D	19
TraesCS4B02G344000	4B	80
TraesCS4D02G339100	4D	80
TraesCS7B02G139400	7B	7
TraesCS7A02G251700	7A	7
TraesCS1D02G307500	1D	24
TraesCS1A02G308100	1A	21
TraesCS6A02G137800	6A	11
TraesCS6D02G126900	6D	9
TraesCS7B02G443900	7B	3
TraesCS7D02G515100	7D	7
TraesCS1A02G096800	1A	0
TraesCS1B02G097500	1B	0
TraesCS1B02G122500	1B	0
TraesCS1A02G094300	1A	0
TraesCS1D02G106600	1D	0
TraesCS1B02G125900	1B	0
TraesCS3D02G361700	3D	7
TraesCS3B02G400500	3B	7
TraesCS1D02G379200	1D	0
TraesCS1B02G392600	1B	0
TraesCS3D02G468400	3D	1
TraesCS3B02G515900	3B	1
TraesCS1A02G021500	1A	26
TraesCS1D02G020900	1D	5
TraesCS1B02G005200	1B	0
TraesCS1D02G021700	1D	2
TraesCS4B02G323300	4B	17
TraesCS4D02G319700	4D	18
TraesCS5D02G552200	5D	24
TraesCS5B02G556300	5B	22
TraesCS4B02G366600	4B	2
TraesCS4D02G360600	4D	2
TraesCS3D02G265100	3D	18
TraesCS3B02g298200	3B	0
TraesCS2A02G447100	2A	53
TraesCS2D02G445900	2D	100
TraesCS2A02G447300	2A	93
TraesCS2D02G446100	2D	59
TraesCS7A02G030700	7A	73
TraesCS7D02G026900	7D	46
TraesCS4D02G359100	4D	83

Chr	gene number
4D	19
4A	17
1A	22
1D	21
4B	15
7B	16
7A	18
6A	18
6D	18
7D	18
1B	22
3D	21
3B	23
5D	16
5B	17
2A	26
2D	26
2B	23
6B	15
3A	21
U0	1
5A	18

TraesCS4A02G459200	4A	83
TraesCS7A02g030600	7A	0
TraesCS7D02g026800	7D	0
TraesCS7A02G030800	7A	75
TraesCS7D02G027000	7D	73
TraesCS2B02G466400	2B	44
TraesCS2D02G444200	2D	38
TraesCS6D02G210900	6D	104
TraesCS6A02G224300	6A	0
TraesCS6B02G257300	6B	100
TraesCS6D02G211400	6D	102
TraesCS2A02G206400	2A	52
TraesCS2D02G209600	2D	46
TraesCS2A02G552400	2A	101
TraesCS2B02G583800	2B	100
TraesCS3A02g200300	3A	0
TraesCS3B02g224500	3B	0
TraesCS2D02G158300	2D	16
TraesCS2A02G152900	2A	18
TraesCS1B02G383600	1B	84
TraesCS1D02G371500	1D	72
TraesCS1A02G365900	1A	82
TraesCS1D02G371400	1D	85
TraesCSU02G069000	U0	0
TraesCS2B02G387700	2B	0
TraesCS6A02G224200	6A	100
TraesCS6D02G210800	6D	100
TraesCS4A02G467100	4A	2
TraesCS7A02G021800	7A	8
TraesCS4A02G459100	4A	83
TraesCS2D02G446400	2D	96
TraesCS2A02G447500	2A	20
TraesCS5A02G534400	5A	0
TraesCS4A02G322200	4A	15
TraesCS5A02G495500	5A	14
TraesCS4D02G319800	4D	17
TraesCS4D02G224500	4D	11
TraesCS1B02G049700	1B	3
TraesCS1B02G023400	1B	73
TraesCS1B02G023500	1B	0
TraesCS1A02G244500	1A	0
TraesCS5B02G056600	5B	6
TraesCS4A02G047900	4A	1
TraesCS1A02G169400	1A	0
TraesCS5A02G512700	5A	80
TraesCS4D02g309700	4D	0
TraesCS4A02g411900	4A	1
TraesCS4B02g312100	4B	7

TraesCS1B02g383900	1B	43
TraesCS1A02g366300	1A	47
TraesCS1D02g371900	1D	0
TraesCS1A02g366200	1A	83
TraesCS1B02g383800	1B	70
TraesCS1D02g371800	1D	83
TraesCS1A02g366100	1A	69
TraesCS1B02g383700	1B	0
TraesCS1D02g371700	1D	46
TraesCS3D02g255400	3D	35
TraesCS3B02g286500	3B	45
TraesCS3A02g254500	3A	55
TraesCS2A02g152700	2A	34
TraesCS2B02g177900	2B	34
TraesCS2D02g158200	2D	35
TraesCS7D02g340300	7D	19
TraesCS7B02g244200	7B	0
TraesCS1A02g083100	1A	35
TraesCS1D02g084400	1D	35
TraesCS1B02g100600	1B	41
TraesCS3A02g251200	3A	27
TraesCS3B02g280700	3B	22
TraesCS3D02g251600	3D	26
TraesCS2B02g252000	2B	37
TraesCS2D02g234000	2D	37
TraesCS2A02g230400	2A	37
TraesCS7B02g204800	7B	99
TraesCS7A02g304500	7A	99
TraesCS7D02g300000	7D	68
TraesCS2A02g370700	2A	33
TraesCS2B02g387500	2B	26
TraesCS2D02g366900	2D	32
TraesCS2A02g370400	2A	30
TraesCS2B02g387800	2B	26
TraesCS2D02g367000	2D	26
TraesCS2D02g366800	2D	34
TraesCS2B02g387300	2B	33
TraesCS2A02g370300	2A	27
TraesCS6A02g236700	6A	45
TraesCS6D02g219300	6D	45
TraesCS6B02g265200	6B	0
TraesCS6D02g043600	6D	23
TraesCS6B02g053100	6B	24
TraesCS6A02g037700	6A	24
TraesCS3B02g109800	3B	78
TraesCS3D02g094500	3D	86
TraesCS3A02g094300	3A	27
TraesCS5B02g105900	5B	89

TraesCS5D02g113300	5D	100
TraesCS5A02g101000	5A	89
TraesCS6D02g298100	6D	33
TraesCS6A02g318900	6A	33
TraesCS6B02g349100	6B	33
TraesCS6A02g319000	6A	18
TraesCS6B02g349200	6B	30
TraesCS6D02g298200	6D	24
TraesCS7B02g085100	7B	28
TraesCS7A02g179900	7A	28
TraesCS7D02g181400	7D	27
TraesCS2D02g324800	2D	100
TraesCS2A02g338200	2A	100
TraesCS2B02g343800	2B	101
TraesCS6B02g265400	6B	87
TraesCS6D02g219500	6D	87
TraesCS6A02g236900	6A	87
TraesCS2A02g370000	2A	61
TraesCS2D02g366400	2D	61
TraesCS2B02g387000	2B	0
TraesCS2A02g381900	2A	100
TraesCS2B02g399200	2B	125
TraesCS2D02g378400	2D	100
TraesCS6B02g257100	6B	100
TraesCS6B02g257200	6B	100
TraesCS6A02g224100	6A	102
TraesCS6D02g211200	6D	103
TraesCS6A02g224000	6A	101
TraesCS6D02g211100	6D	100
TraesCS6A02g224400	6A	100
TraesCS6D02g210500	6D	100
TraesCS6B02g257000	6B	78
TraesCS1D02g154000	1D	0
TraesCS1B02g172500	1B	0
TraesCS1A02g155300	1A	0
TraesCS2A02g445500	2A	59
TraesCS2B02g466500	2B	45
TraesCS2D02g444300	2D	44
TraesCS6A02g265300	6A	101
TraesCS6D02g249200	6D	100
TraesCS6B02g292500	6B	100
TraesCS6D02g274900	6D	21
TraesCS6A02g294000	6A	12
TraesCS6B02g324600	6B	18
TraesCS7D02g208000	7D	24
TraesCS7B02g112400	7B	29
TraesCS7A02g205100	7A	18
TraesCS5D02g210900	5D	11

TraesCS5B02g203100	5B	12
TraesCS5A02g205200	5A	12
TraesCS5D02g326600	5D	72
TraesCS5B02g320800	5B	72
TraesCS5A02g320500	5A	68
TraesCS3D02g377300	3D	96
TraesCS3B02g416400	3B	94
TraesCS3A02g384400	3A	101
TraesCS1B02g279400	1B	56
TraesCS1A02g268800	1A	100
TraesCS1D02g268900	1D	90
TraesCS5D02g335700	5D	101
TraesCS5B02g330100	5B	101
TraesCS5A02g329900	5A	0
TraesCS7B02g169900	7B	101
TraesCS7D02g272400	7D	101
TraesCS7A02g272100	7A	101
TraesCS4B02g096500	4B	116
TraesCS4D02g092900	4D	107
TraesCS4A02g217800	4A	116
TraesCS5B02g119300	5B	101
TraesCS5D02g128200	5D	101
TraesCS5A02g111300	5A	101
TraesCS2D02g446300	2D	47
TraesCS2B02g467900	2B	68
TraesCS2A02g447400	2A	93
TraesCS2A02g447600	2A	94
TraesCS2D02g446500	2D	94
TraesCS2B02g467800	2B	76
TraesCS2B02g467700	2B	100
TraesCS2D02g446000	2D	68
TraesCS2A02g447200	2A	51
TraesCS7D02g407500	7D	7
TraesCS7B02g314200	7B	7
TraesCS7A02g414300	7A	7
TraesCS2D02g068000	2D	24
TraesCS2A02g069300	2A	24
TraesCS2B02g082400	2B	18
TraesCS5A02g405800	5A	21
TraesCS5D02g415800	5D	21
TraesCS5B02g410500	5B	21
TraesCS1B02g265400	1B	11
TraesCS1A02g252600	1A	17
TraesCS6D02g327300	6D	28
TraesCS6B02g377400	6B	23
TraesCS6A02g344700	6A	28
TraesCS5D02g225800	5D	19
TraesCS5B02g216900	5B	24

TraesCS5A02g217900	5A	24
TraesCS1B02g401700	1B	22
TraesCS1A02g379700	1A	23
TraesCS1D02g387100	1D	22
TraesCS3B02g126800	3B	14
TraesCS3D02g109800	3D	6
TraesCS3A02g108000	3A	8
TraesCS3D02g286300	3D	0
TraesCS3A02g286600	3A	2
TraesCS3B02g320800	3B	4
TraesCS2B02g351000	2B	5
TraesCS2D02g331700	2D	4
TraesCS2A02g331200	2A	6
TraesCS4A02g113000	4A	10
TraesCS4D02g192400	4D	15
TraesCS4B02g191200	4B	14
TraesCS2B02g183100	2B	24
TraesCS2A02g157600	2A	23
TraesCS2D02g163700	2D	23
TraesCS5D02g234800	5D	18
TraesCS5A02g227400	5A	16
TraesCS5B02g226100	5B	15
TraesCS4A02g189700	4A	24
TraesCS4B02g129100	4B	24
TraesCS4D02g123300	4D	24
TraesCS5D02g149600	5D	23
TraesCS5A02g142500	5A	23
TraesCS5B02g141200	5B	21
TraesCS7D02g166500	7D	26
TraesCS7A02g165700	7A	26
TraesCS7B02g070400	7B	26
TraesCS4A02g118400	4A	14
TraesCS4B02g186000	4B	17
TraesCS4D02g187100	4D	22
TraesCS3B02g290600	3B	39
TraesCS3A02g257800	3A	41
TraesCS3D02g257800	3D	77
TraesCS6D02g179100	6D	35
TraesCS6B02g228500	6B	35
TraesCS6A02g198000	6A	32
TraesCS7D02g120400	7D	0
TraesCS7B02g020700	7B	0
TraesCS7A02g121900	7A	0
TraesCS3D02g133000	3D	0
TraesCS3A02g307700	3A	0
TraesCS3B02g150900	3B	0
TraesCS3A02g368700	3A	9
TraesCS3D02g361600	3D	9

TraesCS3B02g400200	3B	13
TraesCS5B02g307500	5B	157
TraesCS5D02g314000	5D	128
TraesCS5A02g307100	5A	151
TraesCS5B02g459200	5B	91
TraesCS5A02g450800	5A	108
TraesCS5D02g460800	5D	112
TraesCS2B02g184500	2B	16
TraesCS2D02g165900	2D	14
TraesCS2A02g158900	2A	16
TraesCS4B02g052400	4B	7
TraesCS4D02g052600	4D	9
TraesCS4A02g262500	4A	9
TraesCS4B02g105200	4B	7
TraesCS4D02g102100	4D	13
TraesCS4A02g211100	4A	7
TraesCS3B02g473600	3B	8
TraesCS3A02g439500	3A	5
TraesCS3A02g187800	3A	7
TraesCS3B02g217100	3B	7
TraesCS3D02g191400	3D	7
TraesCS6A02g330400	6A	28
TraesCS1D02g428400	1D	43
TraesCS1A02g420500	1A	28
TraesCS3B02g399300	3B	39
TraesCS3A02g367600	3A	42
TraesCS3D02g360500	3D	42
TraesCS1A02g278900	1A	42
TraesCS1B02g287700	1B	42
TraesCS1D02g278200	1D	51
TraesCS3D02g368000	3D	47
TraesCS3A02g375500	3A	47
TraesCS3B02g407700	3B	47
TraesCS2D02g560100	2D	31
TraesCS2A02g554200	2A	36
TraesCS2B02g589700	2B	36
TraesCS3A02G109300	3A	35
TraesCS5A02g159600	5A	47
TraesCS5D02g164600	5D	44
TraesCS5B02g157300	5B	46
TraesCS6B02g201700	6B	38
TraesCS6D02g162900	6D	22
TraesCS6A02g173800	6A	35
TraesCS7A02g526500	7A	10
TraesCS7B02g443800	7B	10
TraesCS7D02g514800	7D	10
TraesCS7D02g515000	7D	10
TraesCS7B02g444000	7B	7

TraesCS7A02g526600	7A	7
TraesCS3D02g358400	3D	15
TraesCS3B02g397100	3B	14
TraesCS3A02g365200	3A	15
TraesCS1A02g223600	1A	12
TraesCS1D02g225200	1D	12
TraesCS1B02g236900	1B	12
TraesCS4D02g297900	4D	20
TraesCS4A02g006100	4A	17
TraesCS4B02g299000	4B	21
TraesCS4A02g075400	4A	9
TraesCS4D02g232800	4D	1
TraesCS4B02g231300	4B	1
TraesCS4A02g075200	4A	11
TraesCS4D02g232600	4D	1
TraesCS4B02g231200	4B	11
TraesCS5A02g079100	5A	22
TraesCS5D02g093100	5D	18
TraesCS5B02g086900	5B	18
TraesCS1B02g215500	1B	9
TraesCS1A02g201100	1A	9
TraesCS1D02g204500	1D	5
TraesCS7A02g130700	7A	100
TraesCS7D02g130100	7D	73
TraesCS7B02g031000	7B	48
TraesCS7D02g446700	7D	22
TraesCS7B02g357900	7B	21
TraesCS7A02g458700	7A	22
TraesCS7A02g377100	7A	10
TraesCS7D02g373400	7D	10
TraesCS7B02g278400	7B	15
TraesCS3D02g329400	3D	58
TraesCS3A02g336500	3A	31
TraesCS3B02g367500	3B	27
TraesCS2A02g415400	2A	1
TraesCS2D02g412500	2D	1
TraesCS2B02g434400	2B	0
TraesCS2B02g381800	2B	100
TraesCS2A02g363800	2A	100
TraesCS2D02g361600	2D	100
TraesCS6B02g254200	6B	100
TraesCS6D02g207300	6D	100
TraesCS6A02g227700	6A	46
TraesCS4D02g142300	4D	35
TraesCS4B02g143700	4B	35
TraesCS5D02g095600	5D	47
TraesCS5A02g083100	5A	49
TraesCS5B02g089200	5B	0

TraesCS3B02g612200	3B	180
TraesCS3D02g540600	3D	147
TraesCS3A02g535100	3A	146
TraesCS1A02g042700	1A	147
TraesCS1D02g043300	1D	148
TraesCS1B02g055200	1B	146
TraesCS3D02g223700	3D	6
TraesCS3B02g258800	3B	6
TraesCS3A02g229500	3A	5
TraesCS1B02g094500	1B	70
TraesCS1D02g078600	1D	76
TraesCS1A02g076200	1A	76
TraesCS3B02g453300	3B	19
TraesCS3D02g413700	3D	60
TraesCS3A02g418100	3A	93
TraesCS4D02g181100	4D	100
TraesCS4A02g124900	4A	100
TraesCS4B02g179500	4B	100
TraesCS2B02g112600	2B	100
TraesCS2A02g097200	2A	100
TraesCS2D02g096000	2D	100
TraesCS3A02g044700	3A	100
TraesCS3B02g039100	3B	100
TraesCS3D02g036200	3D	100
TraesCS5B02g093000	5B	20
TraesCS5D02g099100	5D	20
TraesCS5A02g087100	5A	20
TraesCS7B02g127800	7B	64
TraesCS7A02g219600	7A	81
TraesCS7D02g222600	7D	68

Table S6 Primer used in this study

	gene name	Forward primer	Reverse primer	product length	melt temperatures	GC%	primer efficiency（%）
For DEGs validation	TraesCS1D02G399000	GTGTACCACCTCCTCTCTTATGGC	AGACAGCAATCTTCCGTACACTTG	72	78.1	43.1	101.0083
	TraesCS1A02G149300	AACTTGTCGGACAGGCTTCACC	ACTCATGAAGATCTCGACGGAACC	66	84	59.1	96.84194
	TraesCS6B02G201600	CCTGAAGACGTGGTGTTGAAGG	CGTTCTTAACCTGGTGGACGTCTG	70	81.3	51.4	105.1267
	TraesCS6B02G286200	TCGCTCCATCCTTACCTGTATGC	TCCTTCCTCCTGAAGGATCAAACC	75	79.3	45.3	100.9692
	TraesCS7A02G333900	TCCATCATCATCACCAAGAAGCTG	TCGAAGTTGATGCCCTTGATGG	143	88	65.7	107.6343
	TraesCS7D02G341500	ATGATCATGCTCGCGCACAAC	GCAAATGCGTTGGCGTAGATGTAG	122	88.5	61.5	99.59795
	TraesCS6A02G173700	ACAGGCCCTGAAGATGTGGTGTTG	GTCGTTCTTGACCTGGTGGATGTC	78	82.5	52.6	101.9201
	TraesCS1D02G176400	GCGTGACAACATCAAGGCCATC	GCTGTGCATCATCTCTGCCATC	100	86.1	58	96.36745
	TraesCS3A02G270100	AGGCTTCCATTTGATGGAGATGTG	AGCTTGGTGCCTTGATGTTCTACC	65	77.3	43.1	96.41407
	TraesCS1B02G167200	GCTACAAGCGCCACAAAGGTTG	TCGTCTTCTAAGTCGTCCATGTCG	76	85	59.2	100.2077
	TraesCS1A02G177800	AGCCATCGGAGCTTGACATGAG	TGCCTGCCGTCGACATTAAC	127	89.6	63.8	104.8726
	TraesCS2D02G354300	ACGGTTCAGCACGTATGCAG	CACAATACAGCTTGGTGGGTTGC	98	85.7	57.1	96.1118
For MYB	TraesCS1B02G100600	TGCCGGTGTATGACAAGAAGCAC	TTTGCTGGCACCTTAGGTCGTG	64	81.3	53.1	96.07317
	TraesCS2B02G177900	AGATTGCAGGCCTTGAGAGGTG	ACTTGTTGCCGAGGATGGAATG	141	88	58.9	104.4213
	TraesCS2D02G158200	AGATTGCAGGCCTTGAGAGATGC	AGGTCCGGCCTCAGGTAGTTTATC	70	83.6	57.1	101.2814
	TraesCS3B02G224500	ACCACCCACGAATCGCTCAATG	TGTGGCGCTAGGAAATCGTCAC	131	89.2	62.6	101.4185
	TraesCS3B02G286500	TCGACCCCAGCACCCACAAGC	TGCCCGCCCCATCGAAGCC	171	87.5	58.4	100.1994
	TraesCS5A02G142500	AGTCCCTCCTTGGCAACAGATG	TTCTTGATGTCGTTGTCCGTTCTC	73	83.5	56.2	100.942

	TraesCS5B02G119300	ATCAACTACCTGAGGCCGGAC	ATCAGCGACCACTTGTTTCCGA	104	84.6	53.8	105.7418
	TraesCS6A02G265300	CGATGCAACAGTGCCATGTACG	CCGAGTTCCTTCTCCAAATCCG	87	86.1	59.8	100.5061
	TraesCS6D02G298200	AGAATAGCACCCAGAAACCG	CTGTTGCCCTCAGCGTGACA	121	86.6	57	104.1127
	TraesCS7A02G165700	AACTACCTCCGGCCGAACATCAAG	TGATGGACCACCTATTGCCAAGC	100	87	60	100.3
KC775782	TaACTIN	CACACTGGTGTTATGGTAGG	AGAAGGTGTGATGCCAAAT	154	84.1	48.7	100.3319
EF592180.1	GAPDH	TTCAACATCATTCCAAGCAGCA	CGTAACCCAAAATGCCCTTG	220	85.2	49.1	100.6817
U76745.1	β-Tubulin	TGTGGCAACCAGATCGGTGC	CATAAGGCCCAGTGCGGACAC	211	86.6	63	98.85
M90077.2	EF-1α	TTTCACTCTTGGAGTGAAGCAGAT	GACCTCCTTGACAATTTCTTCATAA	103	84.1	58	97.23
AK453841.1	eIF-4a	AGGTTCCCAGTTTGACGCTA	CGATTCCCCTTTGCTGGATG	197	83.9	59	109.17
AY049041.1	25SrRNA	GGGTTTAGACCGTCGTGAGA	TTCAGTCATAATCCGGCACA	163	82.8	60	95.29
AY297059.2	UBQ	CATCGACAATGTGAAGGCGA	CTTACCAGCGAAGATCAGGC	79	85.6	59	92.34
AF475127.1	18SrRNA	TTGATGTATACTCGCAATGC	AAATTGCAGATAGCACATTTG	173	84.5	60	95.67

Table S7 Stability values and ranking order of housekeeping genes tested for silicon treatment conditions, based on results from BestKeeper, NormFinder and Delta Ct

Ranking	BestKeeper		NormFinder		Delta Ct	
	Gene	std dev [±CP]	Gene	Stability value	Gene	Average of st dev
1	ACT	0.334	GAPDH	0.005	GAPDH	0.466
2	GAPDH	0.353	ACT	0.016	ACT	0.471
3	β-Tubulin	0.448	β-Tubulin	0.020	β-Tubulin	0.557
4	25SrRNA	0.556	25SrRNA	0.030	25SrRNA	0.685
5	UBQ	0.747	EF-1α	0.048	EF-1α	0.964
6	EF-1α	0.833	UBQ	0.049	UBQ	0.971
7	eIF-4a	1.011	eIF-4a	0.066	eIF-4a	1.338
8	18SrRNA	1.847	18SrRNA	0.092	18SrRNA	2.029
